# 2D Multimodal Image Collection for Fluorescence Prediction from Transmitted Light Microscopy

**DOI:** 10.1038/s41597-026-07004-w

**Published:** 2026-03-24

**Authors:** Dorian Kauffmann, Guillaume Gay, Julio Mateos-Langerak, Oriane Pourcelot, Virginie Georget, Maëlle Carraz, Juliette Van Dijk, Stéphanie Bosch, Elsa Castellani, Qiyan Mao, Laura Ruiz, Valentin Asei-Ceschino, Tudor Manoliu, Louis Ruel, Julia Bonnet-Gelebart, Youssef Elhabouz, Marc Tramier, Jacques Pécréaux, Jean-Bernard Fiche, David Lleres, Christine Doucet, Erwan Gandon, Yves Lutz, Bertrand Vernay, Daniel Stockholm, Abbass Jaber, Aude Moret, Romain Morichon, Mónica Fernández-Monreal, Edouard Bertrand, Emmanuel Faure

**Affiliations:** 1 Laboratoire d’informatique, de robotique et de microélectronique de Montpellier, LIRMM, University of Montpellier CNRS, Montpellier, France; 2 FBI-Core, University of Montpellier, CNRS, UAR2057 Paris, France; 3 Institut de Génétique Humaine, IGH, University of Montpellier, CNRS, Montpellier, France; 4 Montpellier Ressources Imagerie, BioCampus, University of Montpellier, CNRS, INSERM, Montpellier, France; 5 Centre de Recherche en Biologie cellulaire de Montpellier, CRBM, University of Montpellier, CNRS, Montpellier, France; 6 Molecular Cellular and Developmental Biology Unit (MCD), Centre de Biologie Integrative (CBI), University of Toulouse, CNRS, Toulouse, France; 7 UMR 152 Pharma Dev, Université de Toulouse, IRD, 31062 Toulouse, France; 8 Centre de Biologie Intégrative, CBI, Toulouse Réseau Imagerie, TRI - Light imaging Toulouse, LITC, CNRS, University of Toulouse, Toulouse, France; 9 Institut de Biologie du Développement de Marseille, IBDM, CNRS, Marseille, France; 10 Imaging and Cytometry Platform, PFIC, Gustave Roussy Cancer Campus, Villejuif, France; 11 Institut de Génétique et de Développement de Rennes, IGDR, CNRS, University of Rennes, Rennes, France; 12 Centre de Biologie Structurale, CBS, University of Montpellier, CNRS, INSERM, Montpellier, France; 13 Light Microscopy Facility, Institut de Génétique et de Biologie Moléculaire et Cellulaire, IGBMC, INSERM, CNRS, University of Strasbourg, Illkirch, France; 14 Paris Sciences & Lettres Research University, PSL, École Pratique des Hautes Études, EPHE, Paris, France; 15 Genethon’s Imaging Cytometry Platform, ImCy, Genethon, Évry, France; 16 Genethon, University of Paris-Saclay, University of Évry, INSERM, Évry-Courcouronnes, Paris, France; 17 Centre de Recherche Saint-Antoine, CRSA, INSERM, Paris, France; 18 Cytométrie Imagerie Saint-Antoine, Sorbonne University, CRSA, Paris, France; 19 Bordeaux Imaging Center, BIC, University of Bordeaux, CNRS, INSERM, Bordeaux, France; 20 Institut de Génétique Moléculaire de Montpellier, IGMM, University of Montpellier CNRS, Montpellier, France; 21 Institut de Génétique Humaine, IGH, University of Montpellier CNRS, Montpellier, France

**Keywords:** Data acquisition, Data publication and archiving, Databases

## Abstract

We present the Light My Cells Database, a large-scale open-access collection comprising 2,574 acquisition sets and 56,984 microscopy 2D images designed to support the development of machine learning models for fluorescence prediction from transmitted light images. The dataset aggregates data from 30 independent studies conducted across 8 national imaging centers and captures a wide diversity of biological samples, imaging modalities, and acquisition systems. Each transmitted light image - recorded in bright-field, phase contrast, or differential interference contrast -is paired with at least one fluorescence image labeling key subcellular structures: nucleus, mitochondria, tubulin, or actin. All images are standardized in OME-TIFF format and annotated with rich metadata following REMBI guidelines. A dedicated preprocessing pipeline ensures dimensional harmonization, best-focus plane selection, and consistent file naming. The database reflects the variability encountered in real-life microscopy experiments, making it suited for training and benchmarking generalizable deep learning models. It is accessible via the BioImage Archive and supports a range of downstream applications, including in silico labeling, segmentation, and cell profiling from label-free imaging.

## Background & Summary

Fluorescence microscopy has become a cornerstone of cell biology^1^, enabling the visualization and quantification of specific subcellular structures using targeted fluorescent probes. However, this technique remains constrained by several limitations^2^: sample preparation can be labor-intensive and costly, photobleaching reduces signal quality over time, and phototoxicity can alter or damage living cells during imaging. These issues are particularly problematic in long-term or high-throughput experiments, where minimizing light exposure and reducing the number of fluorescent channels is critical^3^.

In contrast, transmitted light microscopy techniques—such as bright-field (BF), phase contrast (PC), and differential interference contrast (DIC)—are non-invasive, label-free, and do not induce photodamage^4^. While these modalities preserve cell viability, they lack the molecular specificity of fluorescence imaging. Bridging this gap has become a central goal in computational microscopy.

**Fig. 1:**
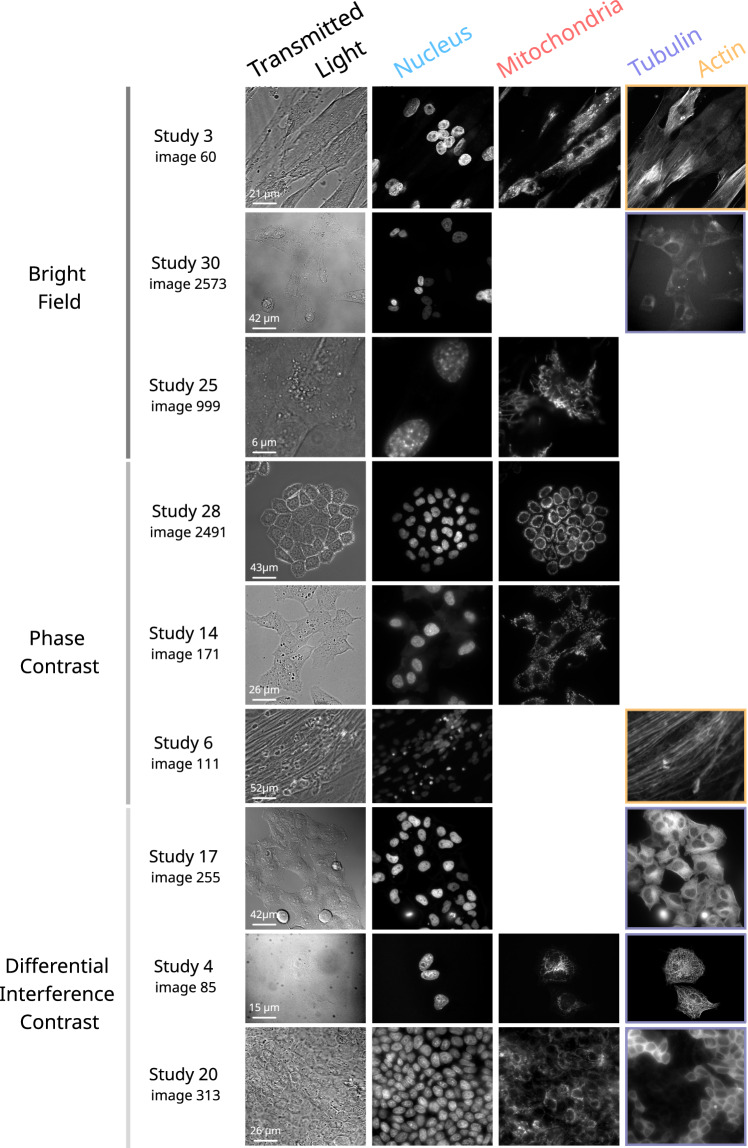
Overview of the Light My Cells database. Each row represents a unique biological sample imaged in transmitted light and in one or more fluorescence channels. The first column shows the transmitted light modality used—either Bright Field (BF), Phase Contrast (PC), or Differential Interference Contrast (DIC)—while the subsequent columns display the corresponding fluorescence images for up to four labeled organelles: nucleus, mitochondria, tubulin, and actin. Rows are grouped according to the transmitted light modality employed. All images are drawn from the published dataset; the corresponding study identifiers are indicated to reference their origin within the database. Images references: image_60_BF_z0.ome.tiff, image_60_Nucleus.ome.tiff, image_60_Mitochondria.ome.tiff, image_60_Actin.ome.tiff, image_2573_BF_z13.ome.tiff, image_2573_Nucleus.ome.tiff, image_2573_Mitochondria.ome.tiff, image_2573_Tubulin.ome.tiff, image_999_BF_z9.ome.tiff, image_2573_Nucleus.ome.tiff, image_999_Mitochondria.ome.tiff, image_2491_PC_z10.ome.tiff, image_2491_Nucleus.ome.tiff, image_2491_Mitochondria.ome.tiff, image_171_PC_z18.ome.tiff, image_171_Nucleus.ome.tiff, image_171_Mitochondria.ome.tiff, image_111_PC_z3.ome.tiff, image_111_Nucleus.ome.tiff, mage_111_Actin.ome.tiff, image_255_DIC_z0.ome.tiff, image_255_Nucleus.ome.tiff, image_255_Tubulin.ome.tiff, image_85_DIC_z0.ome.tiff, image_85_Nucleus.ome.tiff, image_85_Mitochondria.ome.tiff, image_85_Tubulin.ome.tiff, image_313_DIC_z6.ome.tiff, image_313_Nucleus.ome.tiff, image_313_Mitochondria.ome.tiff, image_313_Tubulin.ome.tiff.

To tackle this issue, the Light My Cells database enables the development of deep learning models that predict organelle-specific fluorescence images from transmitted light inputs. To support this objective, we assembled a large, diverse, and standardized dataset of 56,984 microscopy images collected across 30 independent experimental studies (Fig. 1). Each image in transmitted light is paired with at least one fluorescence image targeting a cellular structure—nucleus, mitochondria, tubulin, or actin—thereby supporting direct supervised learning for cross-modality prediction tasks.

The database was created through a nationwide collaborative effort within the France-BioImaging (FBI) infrastructure, involving eight imaging nodes and their associated core facilities. To reflect the heterogeneity of real-world microscopy, data were acquired using a variety of microscopes, cell lines, optical setups, and sample preparations. This diversity is a distinguishing feature of the dataset and supports the development of models that generalize across imaging systems, sample types, and acquisition protocols.

Data collection followed a two-phase protocol. An initial survey with an exploratory acquisition phase was carried out to capture user needs and technical constraints across facilities. This informed the design of a standardized acquisition protocol, which was then implemented across imaging sites. All data contributors provided detailed experimental metadata using a REMBI^5^-compliant template, and image files were converted to the OME-TIFF^6^ format to ensure compatibility with major open-source software and FAIR^7^ principles.

A dedicated preprocessing pipeline harmonized image dimensionality, extracted best-focus^8^ planes from z-stacks, and implemented a consistent file-naming convention. Transmitted light z-stacks were preserved in full, while fluorescence channels were reduced to their best-focus plane to match the intended prediction task.

Several approaches have explored fluorescence prediction from transmitted light microscopy, including DeepHCS^9^, Label-Free Prediction of Cell Painting^10^, and In Silico Labeling^11^. While the latter incorporates multiple transmitted light modalities—similar to those in the Light My Cells dataset—the underlying databases in these studies remain relatively constrained. DeepHCS relies on fixed image sizes and predefined fluorescent dyes, while In Silico Labeling employs non-standard data formats that hinder reuse. The Cell Painting approach improves upon this but still lacks diversity in imaging conditions and biological models. Although the JUMP-CP^12^ database offers a more extensive cell painting resource, no open-access dataset to date has combined the level of heterogeneity, paired imaging modalities, and standardized metadata provided by the Light My Cells Database.

The complete dataset is openly accessible through the BioImage Archive (See Data Records section), a curated public repository for biological imaging that ensures long-term accessibility and promotes broad data reuse across imaging modalities, particularly in connection with peer-reviewed studies and reference challenges.

### Community-driven design and exploratory phase

The Light My Cells project was initiated through a collaborative, bottom-up process coordinated by the France-BioImaging (FBI) national infrastructure. The primary objective was to create a biologically relevant and technically feasible image collection for developing and evaluating deep learning models capable of generating fluorescence images from transmitted light microscopy. To ensure that the resulting dataset would address real-world use cases and reflect current imaging practices, the project began with a broad community consultation across the FBI network.

A comprehensive online survey was designed and distributed to imaging facility staff, image analysts, and researchers affiliated with FBI. This survey aimed to collect structured information on common imaging workflows, types of microscopes and modalities used, biological models typically studied, fluorescence labeling strategies, and common data formats. The questionnaire also included open-ended sections to capture unmet needs and technically ambitious but desirable imaging scenarios. In total, 45 responses were received, primarily from facility engineers and technical staff, with additional input from postdoctoral researchers and doctoral students.

**Fig. 2:**
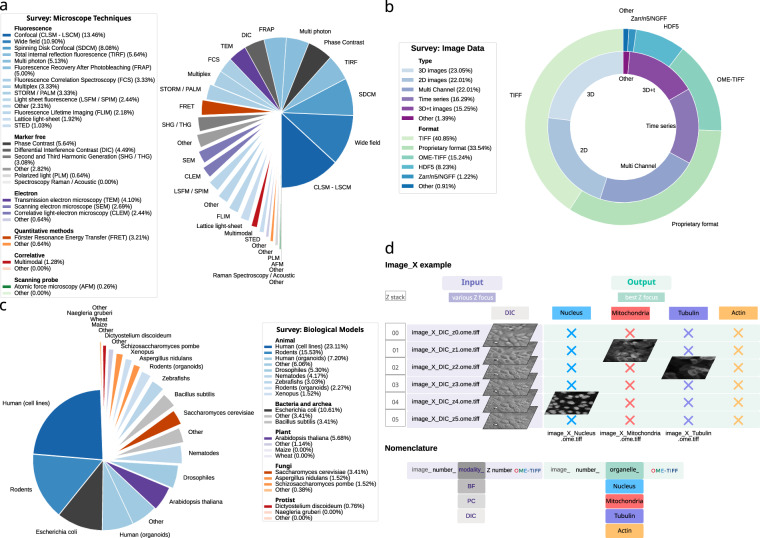
Community survey results and database organization. (**a**) Summary of responses to the France-BioImaging survey regarding the types of microscopes routinely used, grouped by major microscopy categories: fluorescence, label-free, electron, quantitative, correlative, and scanning probe microscopy. (**b**) Types (inner chart) and file formats (outer ring) of images most commonly used by respondents. (**c**) Reported biological models studied by participants, across major taxonomic groups: animals, bacteria and archaea, plants, fungi, and protists. (**d**) Top: schematic illustration of data organization in the Light My Cells database. For each imaged sample, the fluorescence image (at least one channel) is provided at its best focal plane and is paired with a full z-stack acquired in transmitted light. Bottom: standardized file naming scheme for transmitted light (input) and fluorescence (output) images. Each transmitted light image includes a modality label (BF, PC, or DIC) and the z-plane index. Each corresponding fluorescence image is labeled by the target organelle—nucleus, mitochondria, tubulin, or actin—and represents the selected best-focus plane associated with the same sample and study. Stylized example for illustrative purposes, adapted from image 399 (source path: https://ftp.ebi.ac.uk/biostudies/fire/S-BIAD/047/S-BIAD1047/Files/Images/Study_24/ image_399_DIC_z[0,…,5].ome.tiff, image_399_Nucleus.ome.tiff, image_399_Mitochondria.ome.tiff, and image_399_Tubulin.ome.tiff.

The survey revealed substantial heterogeneity (see Fig. 2a–c) in imaging conditions across France-BioImaging platforms, both in terms of biological models and technical setups. A wide range of cell types, transmitted light modalities—including bright-field (BF), phase contrast (PC), and differential interference contrast (DIC)—and labeling strategies were reported. Despite this diversity, the responses converged on a shared set of technically feasible and scientifically useful configurations. In particular, live mammalian monolayer cultures imaged simultaneously in transmitted light and fluorescence were identified as a robust and achievable starting point. Although the imaging of 3D systems such as organoids was frequently identified as a desirable objective, it was considered too technically heterogeneous to be incorporated in the initial version of the dataset.

Based on this consultation, an initial exploratory acquisition protocol was developed and implemented across a subset of FBI imaging nodes. The aim of this phase was to assess the reproducibility of paired imaging strategies under routine conditions and to evaluate the practical aspects of harmonizing data formats and metadata documentation across sites. Contributors were invited to acquire paired transmitted light and fluorescence images of live-cell samples using their existing imaging infrastructure and experimental protocols. Fluorescence channels were selected based on their prevalence and interpretability, with nuclear staining being the most common, followed by mitochondrial, tubulin, and actin labeling. Contributors retained flexibility in the choice of fluorophores and microscopes, provided that minimum documentation was supplied on image acquisition settings and sample preparation.

The exploratory datasets confirmed the feasibility of multi-site data collection with sufficient standardization for integration, while preserving a realistic diversity of conditions. They also highlighted the importance of clear metadata standards, particularly for acquisition parameters such as objective magnification, pixel size, numerical aperture, and labeling method. This phase led to the refinement of the metadata template used in subsequent phases of the project, which was structured in accordance with the REMBI^5^ recommendations for biological imaging. Overall, the exploratory phase provided essential validation of the project’s scope and implementation strategy, establishing a solid foundation for the standardized acquisition phase that followed.

## Methods

### Standardized acquisition protocol

Building on the insights gathered during the exploratory phase, a standardized acquisition protocol (see Supp. Methods. 1) was established to ensure consistency across datasets while accommodating the technical diversity of imaging platforms within the France-BioImaging network. This protocol enabled the collection of high-quality, reproducible image pairs suitable for cross-modality learning tasks, without imposing constraints that would exclude representative biological or instrumental variability.

The protocol was disseminated through an open call for data contributions launched at the end of August 2023 and remained open until December of the same year. It targeted all FBI imaging nodes and their affiliated research teams. Contributors were allowed to use their existing microscopes and workflows, provided that minimal criteria were met in terms of imaging conditions, fluorescence labeling, and metadata documentation.

Data acquisition focused on live mammalian cells images according to the standardized protocol. Transmitted light microscopy included at least one modality among bright-field, phase contrast, or differential interference contrast. Fluorescence **i**maging was performed on the same field of view, labeling at least the nucleus, with optional inclusion of other structures such as mitochondria, tubulin, or actin. While time-lapse acquisition was no longer mandatory, it remained encouraged when feasible. Imaging was restricted to widefield modalities, with structured illumination, and spinning-disk systems excluded to maintain homogeneity in optical sectioning.

Contributors were encouraged to acquire multiple fields per sample and to image several biological replicates under each condition. Although the protocol recommended using water immersion objectives with magnifications between 40× and 100×, a range of magnifications and pixel sizes was accepted, provided that subcellular resolution was preserved. Contributors were allowed to use different microscopes and objectives across acquisition sets within a given dataset, reflecting the operational diversity of imaging facilities, while all transmitted-light and fluorescence image pairs within an image set were acquired using the same microscope and objective. For both transmitted-light and fluorescence acquisition set, full z-stacks were acquired at the time of image acquisition—typically 10 to 20 planes. While transmitted-light z-stack images were systematically retained to support focal plane variability, each channel of the fluorescence Z stack images was selected independently as a single optimal focal plane (see Supp. Methods 2.4). Then, the best-focus plane may differ between the fluorescent channels (see Fig. 2d). Each z-index—for both the focus or the best focus for a selected image—is explicitly recorded in the metadata, ensuring traceability back to the original Z-stack. The number of acquired channels was minimized to reduce phototoxicity and to align with the downstream modeling objective of predicting a small set of specific organelle labels.

All submitted image files had to be in lossless formats compatible with open-source software, with a strong recommendation to use OME-TIFF^6^ when possible. In addition, contributors were required to complete a structured metadata template detailing both technical and biological parameters of the experiment. The template followed the REMBI^5^ guidelines and captured information such as instrument configuration, imaging modality, optical settings, sample taxonomy, and labeling strategy. This standardization effort was critical to ensuring the findability, interoperability, and reusability of the dataset, in alignment with FAIR^7^ data principles.

**Fig. 3:**
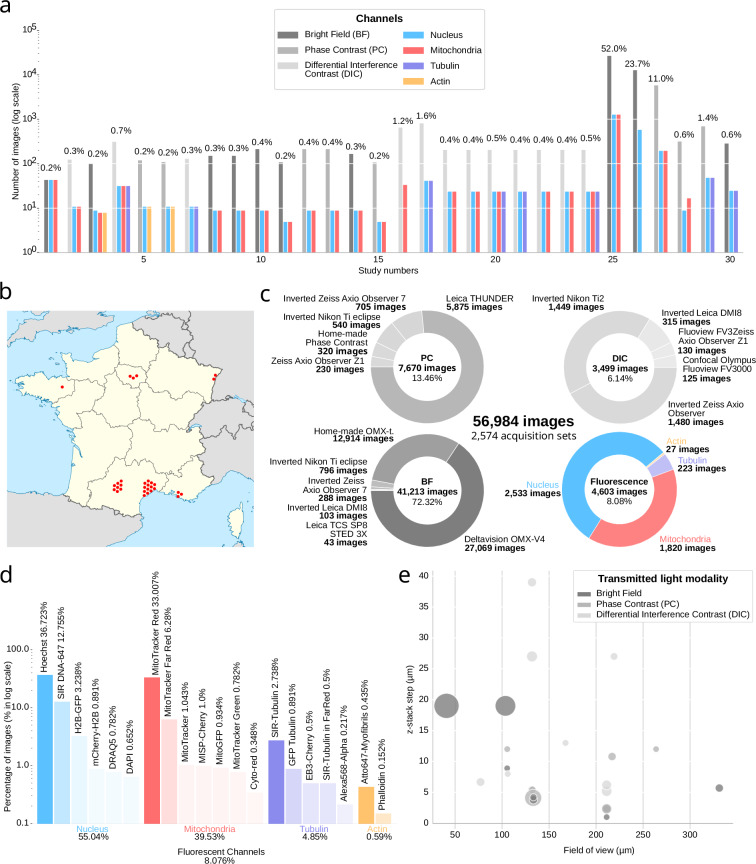
Biological and technical heterogeneity across the Light My Cells database. (**a**) Distribution of the number of images and associated fluorescence channels per study, shown on a logarithmic scale. (**b**) Geographical distribution of acquisition sites across France, illustrating the national coverage of the France-BioImaging network. Each point on the map corresponds to the site of acquisition for one of the 30 studies included in the database. (**c**) Left: proportions of transmitted light modalities (BF, PC, DIC) and their associated fluorescence channels across the full dataset. Right: diversity of instruments used for transmitted light acquisition, and corresponding fluorescence imaging setups. (**d**) Diversity of fluorescent labeling strategies used across the database. For each fluorescence channel, the distribution of fluorophores or dyes is shown, ranked by frequency (logarithmic scale). (**e**) Physical image dimensions for each transmitted light modality, illustrating variability in lateral field of view and axial depth. Lateral dimensions are computed by combining image resolution with physical pixel size, while axial depth corresponds to the physical z-stack extent derived from the number of acquired z-planes and axial sampling.

By the end of the submission period, the project had received contributions from 8 out of the 10 FBI imaging nodes (Fig. 3b). In total, 30 independent studies (Fig. 3a) were collected, comprising 2,574 acquisition sets (see Data Records section). These datasets formed the core of the Light My Cells Database and served as the input for the preprocessing pipeline described in the following section.

### Preprocessing and standardization pipeline

Due to the distributed nature of the data collection process and the technical heterogeneity across contributing imaging platforms, a robust preprocessing pipeline was developed to ensure consistency, interoperability, and ease of reuse. This pipeline addressed key aspects of data standardization, including file format unification, metadata integration, image dimensionality normalization, best-focus plane selection, and file naming harmonization (see Supp. Notes 1 for a complete overview of the pipeline).

Raw image files were acquired in a wide range of native microscopy formats, including.czi (Zeiss),.lif (Leica),.nd2 (Nikon),.oir (Olympus),.dv (DeltaVision), and proprietary or semi-standardized TIFF variants. To ensure long-term accessibility and compatibility with open-source software tools, all image files were converted to the OME-TIFF^6^ format, which combines multi-dimensional image data with embedded XML-based metadata structured according to the OME data model (see Supp. Figure 1). This format was chosen for its interoperability with major visualization and analysis platforms such as ImageJ^13^, Napari^14^, and Python libraries including Tifffile^15^, AICSImageIO^16^, and OpenSlide^17^.

Metadata integration was a central step in the standardization pipeline. Each contributing site provided detailed experimental metadata using the REMBI^5^-based template defined during the acquisition phase. These metadata included both technical descriptors, such as objective magnification, numerical aperture, and pixel size, and biological descriptors, such as sample model, cell type, labeling strategy, and targeted organelles. Custom Python routines were developed to parse and map the metadata into the OME-XML^18^ schema. Attributes not natively supported by the OME model, such as sample-level taxonomy or labeling dye, were embedded in the MapAnnotation section of each file to ensure full integration without compromising software compatibility.

Original images varied in dimensionality, with some datasets including multiple z-planes, time series, and multiple fluorescence channels. To harmonize the data structure for downstream computational workflows, all images were converted to a 2D-equivalent representation, preserving XY resolution while collapsing Z, and C axes into singleton dimensions when applicable (see Supp. Figure 2). Prior to this reduction, a modality-specific best-focus selection was performed on z-stacks^8^. For transmitted light images, the best focal plane was determined using the Normalized Variance metric, which emphasizes sharpness based on local intensity changes. For fluorescence images, an Autocorrelation-based metric was used, optimized for high-contrast signals from labeled structures. Both methods were benchmarked and reimplemented in Python to ensure reproducibility and compatibility. Best-focus planes were extracted per modality and per channel independently, and the corresponding z-plane indices were recorded to maintain traceability.

Fluorescence images were retained only as single-plane best-focus images, in line with the fluorescence prediction task In contrast, the full transmitted light z-stacks were preserved, with the best-focus plane explicitly indexed in the metadata. This dual approach supported both minimal input configurations and more advanced modeling strategies that incorporated axial information from transmitted light modalities.

To support human readability and automatic parsing, all image files were renamed using a standardized convention encoding (see Supp. Methods. 2.5) key metadata elements, including study ID, transmitted light modality, fluorescence channel, and z-plane index when relevant (Fig. 2d bottom). This naming scheme ensured that corresponding transmitted light and fluorescence image pairs can be reliably matched without external lookup tables, and that users can easily explore and manipulate the dataset with simple scripts or graphical tools.

To ensure full interoperability with public repositories and compliance with FAIR^7^ data principles, the dataset was structured according to the study-centric model used by the BioImage Archive^19^. The final dataset comprises 56,984 microscopy images grouped into 30 independent studies, which together include a total of 2,574 acquisition sets, each corresponding to a specific experimental acquisition carried out during the standardized acquisition phase. Each study aggregates a coherent set of images acquired under defined biological and technical conditions, and is annotated with complete experimental metadata. Images are indexed with unique ascending identifiers across all studies, enabling unambiguous file referencing and reproducible data retrieval. The combined use of standardized formats, rich metadata, and consistent organization ensured that the Light My Cells dataset was findable, accessible, interoperable, and reusable, and can be readily integrated into automated analysis pipelines, machine learning workflows, and long-term data repositories.

## Data Records

The Light My Cells database is publicly available at the BioImage Archive20 under BioStudies accession S-BIAD104721^20^. The BioImage Archive is served through the BioStudies infrastructure; access, download, and programmatic retrieval via the BioStudies API are documented in the official BioStudies user guide (https://www.ebi.ac.uk/biostudies/help).

In the BioImage Archive, each dataset unit is represented as a Study Component. These Study Components correspond one-to-one with the studies defined in this paper following the REMBI12 specification, such that each REMBI study is implemented as a single BioImage Archive Study Component. Each Study Component is available as a separate archive via BioStudies and each associated FileList is available in both public archive and Supplementary Notes 1.

The archive contains 56,984 image files, corresponding to 2,574 acquisition sets indexed from 0 to 2573. An acquisition set represents a single biological field of view and groups all files acquired for that scene, including multiple transmitted-light modalities, multiple fluorescence channels, and multiple axial (Z) positions when available. Thus, each acquisition set contains all observations of the same cellular scene across modalities, fluorescence targets (organelles) and focus planes.

Within each Study Component, images are organized by their associated FileList (ex FileList/Study_2.json), which lists all files belonging to that study and provides their identifiers and file paths. Each Study Component is further associated with the Biosample, Specimen, and Image Acquisition sections in the BioImage repository, which provide details about the organism, biological preparation, imaging instrument, and acquisition parameters.

All images include full standardized metadata, providing consistent descriptions of imaging modality, Z position, pixel size, acquisition settings and experimental context. In addition, images follow a consistent file-naming convention (see Supplementary Methods 2.5) that encodes the image-set identifier, transmitted-light modality, axial offset and available fluorescence channels, enabling programmatic grouping of files belonging to the same acquisition set. All data are distributed under the Creative Commons Attribution 4.0 International (CC BY 4.0) license, permitting unrestricted use, redistribution, and reproduction of the data in any medium, provided that appropriate credit is given to the original creators and the source.

## Data Overview

The released dataset comprises a total of 56,984 previously unpublished two-dimensional microscopy images in OME-TIFF6 format, each accompanied by consistent machine-readable structured metadata.

Among these 56,984 images, which correspond to 2,574 acquisition sets (see Data Records), 52,382 correspond to transmitted light acquisitions: 41,213 were captured using bright-field (BF), 7,670 using phase contrast (PC), and 3,499 using differential interference contrast (DIC). Each transmitted light image is paired with at least one fluorescence image from the same sample and field of view. The fluorescence subset contains 4,602 images in total, including 2,533 labeled for the nucleus, 1,819 for mitochondria, 223 for tubulin, and 27 for actin (see Fig. 3a). These fluorescence images correspond to the best-focus planes selected from limited z-stacks, as described in the preprocessing section (see Supp. Table 1).

### Study-level overview and data heterogeneity

The Light My Cells Database was assembled from thirty independent imaging studies, each representing a distinct experimental acquisition conducted under specific biological and technical conditions. To ensure reproducibility and facilitate reuse, a complete and detailed description is provided for each study. These descriptions, available in the Supplementary Notes 1, include structured metadata and standardized annotations that capture the diversity of the dataset.

Each study is documented using a consistent six-part format. It begins with contributor information, including individual roles and institutional affiliations, highlighting the broad engagement of imaging core facilities across the France-BioImaging (FBI) network (Fig. 3b). Representative examples of transmitted light and fluorescence images are included to visually illustrate the type of content available in each study. This is followed by a quantitative summary of the image data published in the BioImage Archive^21^, detailing the number of files, their dimensional characteristics, and modality-specific information. These metrics reveal substantial variation in the number of images contributed across studies (Fig. 3c), as well as variability in image size, resolution, and stack depth (Fig. 3e). This heterogeneity reflects the diversity of real-world microscopy configurations and reinforces the need for standardized metadata to enable consistent interpretation, filtering, and reuse of the data in downstream computational and machine learning workflows. In addition, file-level metadata such as z-stack depth, imaging modality, image format, and study identifier are systematically recorded to support accessibility and downstream integration.

Each study description also includes a section dedicated to experimental design, capturing the biological context of the data. This includes the type of cellular model used, the corresponding NCBI taxonomy ID, sample preparation and culture protocols, and details of the fluorescent labeling strategy. The metadata reflect a wide range of labeling approaches across the dataset, covering multiple fluorophores and targeting different subcellular structures (Fig. 3d). Technical imaging parameters are likewise described in detail, including the make and model of microscopes and detectors, objective characteristics (such as immersion type, numerical aperture, magnification, working distance, and refractive index), and additional descriptors such as the number of z-planes acquired, pixel dimensions, and file format. Despite the use of only three transmitted light modalities (BF, PC, and DIC), the dataset encompasses a broad diversity of imaging systems and acquisition configurations, as illustrated in (Fig. 3c). To ensure traceability and allow for advanced analyses, the raw acquisition parameters were preserved for all original images. This enables further technical validation, supports evaluation of the standardization process across studies, and provides a foundation for future meta-analyses.

Altogether, these study-level records capture the biological and technical heterogeneity that defines the richness of the Light My Cells database. They provide essential context for interpreting the images, enable transparent reuse, and support the development of robust and generalizable computational models. The standardized documentation serves both as a practical guide for end users and as a reproducibility reference, linking each image to its full experimental provenance.

## Technical Validation

To ensure the integrity, reliability, and reusability of the Light My Cells Database, a multi-level technical validation protocol was implemented. This process involved the verification of image file quality, the consistency and completeness of metadata, the correctness of best-focus plane extraction, and the compatibility of the dataset with commonly used open-source image analysis tools.

All submitted image files were automatically screened for corruption, unreadability, or invalid content (e.g., entirely black or empty images). Files failing these checks were excluded. Each valid file was verified for proper OME-TIFF^6^ structure, including the presence of readable OME-XML^18^ metadata and adherence to the naming convention.

Metadata completeness and REMBI^5^ compliance were evaluated across all studies. Required fields describing sample type, labeling strategy, instrument settings, and acquisition parameters were checked. When necessary, contributors were contacted to correct or complete missing information. Finalized metadata were mapped to the OME data model and validated using schema checks.

The accuracy of best-focus plane selection was assessed by expert review. One transmitted light stack and one fluorescence stack per study were randomly sampled and reviewed independently by two experts. In case of disagreement, a third reviewer arbitrated. Most selected planes were confirmed to be optimal; all outlier cases were systematically examined, and images were excluded when imaging artifacts or focus selection issues were identified.

Compatibility testing was conducted using ImageJ^13^, Napari^14^, and Python libraries such as Tifffile^15^, AICSImageIO^16^, and OpenSlide^17^. Custom scripts were developed and provided with the dataset to verify file readability, metadata structure, and image pairing.

This validation process ensures that the database is technically robust, machine-actionable, and ready for reuse in computational workflows, in full alignment with FAIR^7^ principles, with publicly available custom scripts and Jupyter notebooks enabling dataset exploration and image selection by modality or fluorescence staining.

## Usage Notes

The Light My Cells Database is designed to support a wide range of computational tasks involving cross-modality image translation, with a focus on the prediction of fluorescence signals from transmitted light microscopy. To facilitate access and integration into image analysis workflows, we provide a companion Git repository that includes tools for browsing, downloading, and processing the dataset directly from the BioImage Archive^21^.

The repository offers Python scripts for listing and filtering image files by modality, organelle, study, or metadata attributes. It also includes readers compatible with the OME-TIFF^6^ format, based on commonly used libraries such as Tifffile^15^, AICSImageIO^16^, and OpenSlide^17^. These tools enable direct access to image arrays and metadata structures, supporting both interactive exploration and automated processing pipelines.

In its initial release, the database was used in the context of the Light My Cells deep learning challenge, where participants developed models to infer best-focus fluorescence images from transmitted light inputs. The dataset remains suitable for this task, but also supports a broader range of applications, including segmentation, structure-specific detection, label-free cell profiling, and weakly supervised learning. The availability of z-stacks in transmitted light further opens opportunities for depth-aware modeling and focus estimation.

Users are encouraged to refer to the structured metadata provided with each study, which enables reproducible filtering by biological context, imaging conditions, or experimental design. The diversity of modalities, labeling strategies, and acquisition settings included in the dataset makes it particularly suitable for benchmarking model generalization across varied imaging scenarios.

Future updates may include additional tooling or expanded metadata to support new use cases. Contributions or feedback from the community are welcome and can be submitted through the associated repository.

## Limitations

While the Light My Cells database was designed to reflect the diversity of real-world microscopy experiments, this heterogeneity also introduces several limitations that should be considered when reusing the data. First, the dataset is restricted to two-dimensional representations and does not include fully annotated three-dimensional fluorescence volumes, as fluorescence z-stacks were reduced to their optimal focus planes

In addition, for a small subset of studies relying on transient transfection or expression systems, not all cells visible in the fluorescence images may be labeled. Therefore, some fluorescence images can contain a mixture of labeled and unlabeled cells, potentially limiting their direct use for training models that assume exhaustive cell-level fluorescence annotation. Nevertheless, these data remain suitable for alternative workflows, such as instance-level or patch-based training strategies, where individual fluorescent cells can be selected prior to model training.

Finally, the database exhibits significant imbalance in fluorescence labeling strategies and channel representation: the number of images available for actin and tubulin is substantially lower than for nucleus and mitochondria. This imbalance may affect the training and evaluation of machine learning models, especially those sensitive to class distribution or requiring comparable sample sizes across targets. Users may therefore need to apply channel-specific sampling strategies, data augmentation, or task-specific training schemes depending on their intended application.

## Supplementary information

Supplementary Informations

Supplementary Table 1

## Data Availability

All data described in this Data Descriptor are publicly available. The Light My Cells dataset is deposited in the BioImage Archive under the accession number S-BIAD1047 and can be accessed at https://www.ebi.ac.uk/biostudies/BioImages/studies/S-BIAD1047. The repository provides access to all microscopy image files in OME-TIFF format, associated standardized metadata, and documentation describing the dataset structure, file organization, and variable definitions. No restrictions apply to the access or reuse of the data.

## Code availability

All code used for data conversion, metadata harmonization, best-focus plane extraction, dataset validation, and interoperability checks is openly available through the Light My Cells public repository hosted on Codeberg: https://codeberg.org/FranceBioImaging/LightMyCellsDatabase. The repository provides Python scripts supporting the complete dataset preparation workflow, including file organization, OME-XML metadata parsing and standardization, focus metric computation, OME-TIFF format validation, and utilities for downloading and filtering the dataset directly from the BioImage Archive. These scripts were used during dataset construction and verification and are provided as a reproducibility companion to the published data. The code is released under the MIT License to ensure transparency, reproducibility, and reusability.
