# Supplementary material for: 2D Multimodal Image Collection for Fluorescence Prediction from Transmitted Light Microscopy
Source: Sci Data. 2026 Mar 24;13:743. doi: 10.1038/s41597-026-07004-w (PMC13184337; doi:10.1038/s41597-026-07004-w)
Supplement: Supplementary file 1 — Supplementary Informations [file 41597_2026_7004_MOESM1_ESM.pdf]

# Supplementary Informations

**Supplementary Methods 1:** Standardized Acquisition Protocol

**Supplementary Methods 2:** Preprocessing and Harmonization Pipeline

**Supplementary Figure 1 :** Architecture of the image standardized metadata.

**Supplementary Figure 2 :** Illustration of the Image shape normalization process.

**Supplementary Notes 1:** Comprehensive Study-Level Metadata and Imaging Descriptions

**Supplementary Table 1:** Overview of the Light My Cells database collection.

# **Supplementary Methods 1:** Standardized Acquisition Protocol

FRANCE-BIOIMAGING

## Data acquisition framework

### Light My Cells: Bright-field to fluorescence imaging challenge

- Chosen model: **Mammalian cells** (human, rodent, etc.)
- Observation of different **subcellular structures** on a **cellular scale**
- Experimental conditions: **LIVE cell culture** (no fast phenomena)
- **Wide field microscope**
- **Number of sample preparation by person : ~ 5**
- **Number of images / sample : > 10** (capturing several views from the same plate)
- **Objective**: free from 40x to 100x
- Type of imaging :
  - ➢ **2D with few z slides with different focus** (Δz free)
  - ➢ **1 stack with 2 (or more) channels** (around 10 to 20 planes):

    Channel :

    1. **Bright Field, Phase Contrast and/or DIC**
    2. **Fluorescence** imaging of **Nucleus marker**
    3. (encouraged) **Fluorescence** imaging of structures (in order of preference):
       - **Mitochondria** (ex: mitotracker or fluorescent protein)
       - Tubulin (ex: SIR-tubulin or fluorescent protein)
       - Actin (ex: SIR-actin or fluorescent protein)

  - ➢ Multiple microscopes are encouraged (even for the same plate)
  - ➢ Multiple objectives and magnifications are also welcome
  - ➢ Warning : **Minimal movement shift between fluorescent and phase contrast**
  - ➢ **No time-lapse required**

- No SPECIFIC **wavelength** (to be kept in the metadata)
- **Format**: FREE (but can be opened in ImageJ &Napari)
- **Metadata** to **be kept**:
  - ➢ Instrument metadata (objective magnification and **NA**, physical pixel size …)
  - ➢ Sample metadata (species, lineage, staining / contrast method, etc.)
  - ➢ **REMBI template** to complete (1 column per experiment) HERE

- **EXPORT**: File Sender https://filesender.renater.fr/ with your institutional account, contact us if you have any issues with the transfer.

**Contact :** dorian.kauffmann@france-bioimaging.org

# Examples:

## 1. From Virginie Georget, MRI-CRBM Imaging facility -Montpellier

a) DIC

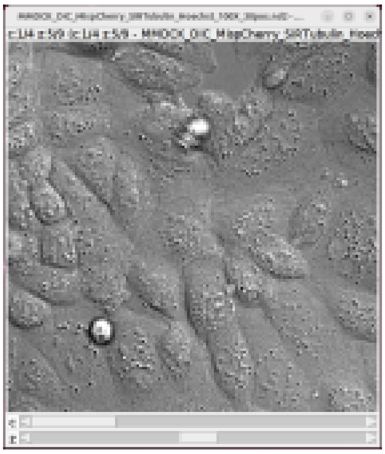

b) Hoechst

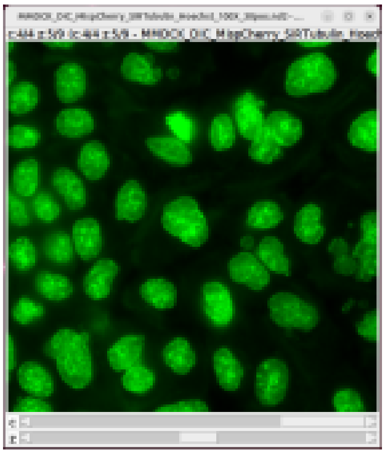

c) MispCherry

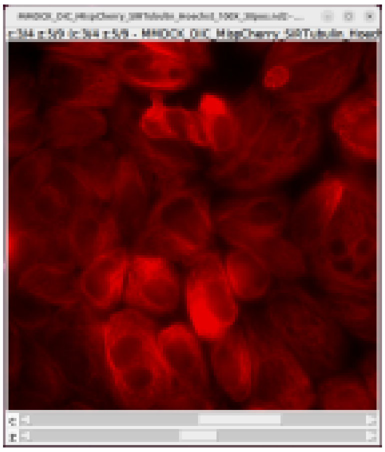

d) SIRTubulin

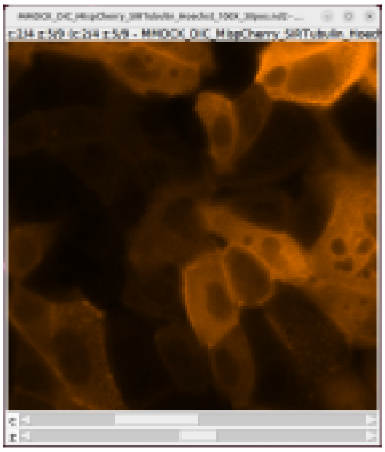

## 2. From Stéphanie Bosch and Maelle Carraz, CBI Toulouse (TRI-LITC & CBI-CNRS)

a) Bright Field

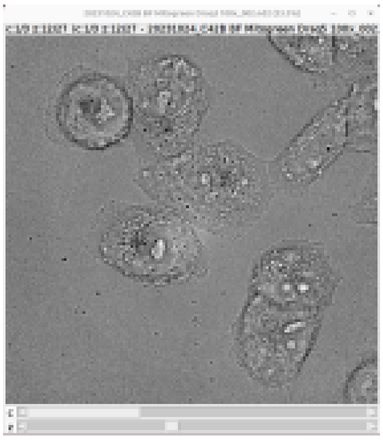

b) Draq5

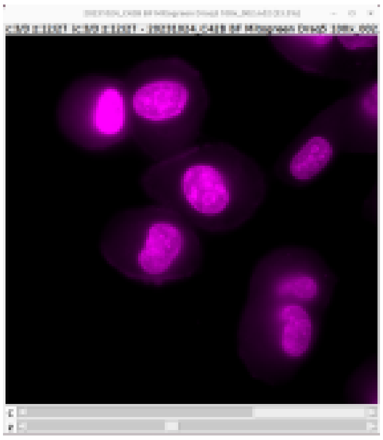

c) MitoGreen

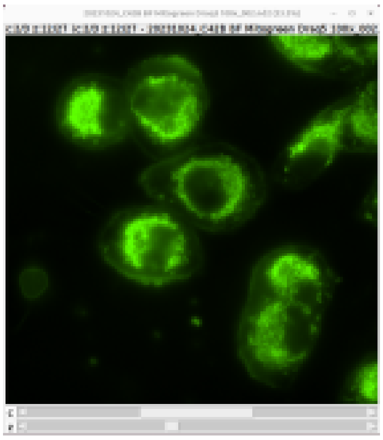

# **Supplementary Methods 2:** Preprocessing and Harmonization Pipeline

To address the heterogeneity of the raw microscopy data contributed by different imaging core facilities, we developed a comprehensive preprocessing and harmonization pipeline. This pipeline ensures that all images in the Light My Cells Database are consistent in structure, accessible to a broad range of users, and compatible with downstream computational workflows. The technical steps are detailed below.

## 1. File Format Unification

The submitted datasets spanned seven distinct file formats: .oir (Olympus), .lif (Leica), .czi (Zeiss), .nd2 (Nikon), .dv (DeltaVision), .tiff, and .ome.tiff. Each format encodes image data and metadata differently and may require proprietary software to access. To ensure accessibility and interoperability, all files were converted into the open and standardized OME-TIFF format developed by the Open Microscopy Environment (OME). OME-TIFF embeds structured OME-XML metadata within a multi-page TIFF container, allowing for both flexible data storage and preservation of rich acquisition metadata. This conversion was performed individually for each dataset to ensure uniform structure and metadata integrity. The resulting files are compatible with widely used image analysis tools such as ImageJ, Napari, Tifffile, OpenSlide, and AICSImageIO.

## 2. Metadata Structuring and FAIR Compliance

Contributors completed a metadata template based on the REMBI guidelines, capturing instrument configurations (e.g., objective magnification, numerical aperture, pixel size), acquisition settings (e.g., channel count, z-stack depth), and biological descriptors (e.g., species, cell type, staining method, organelles labeled). These metadata were mapped into the OME-XML data model using the OME Types Python library. While most fields were natively supported, additional biological descriptors—such as sample taxonomy—were integrated through the MapAnnotation section of the metadata. This approach ensured full machine readability, traceability, and alignment with FAIR (Findable, Accessible, Interoperable, and Reusable) data principles.

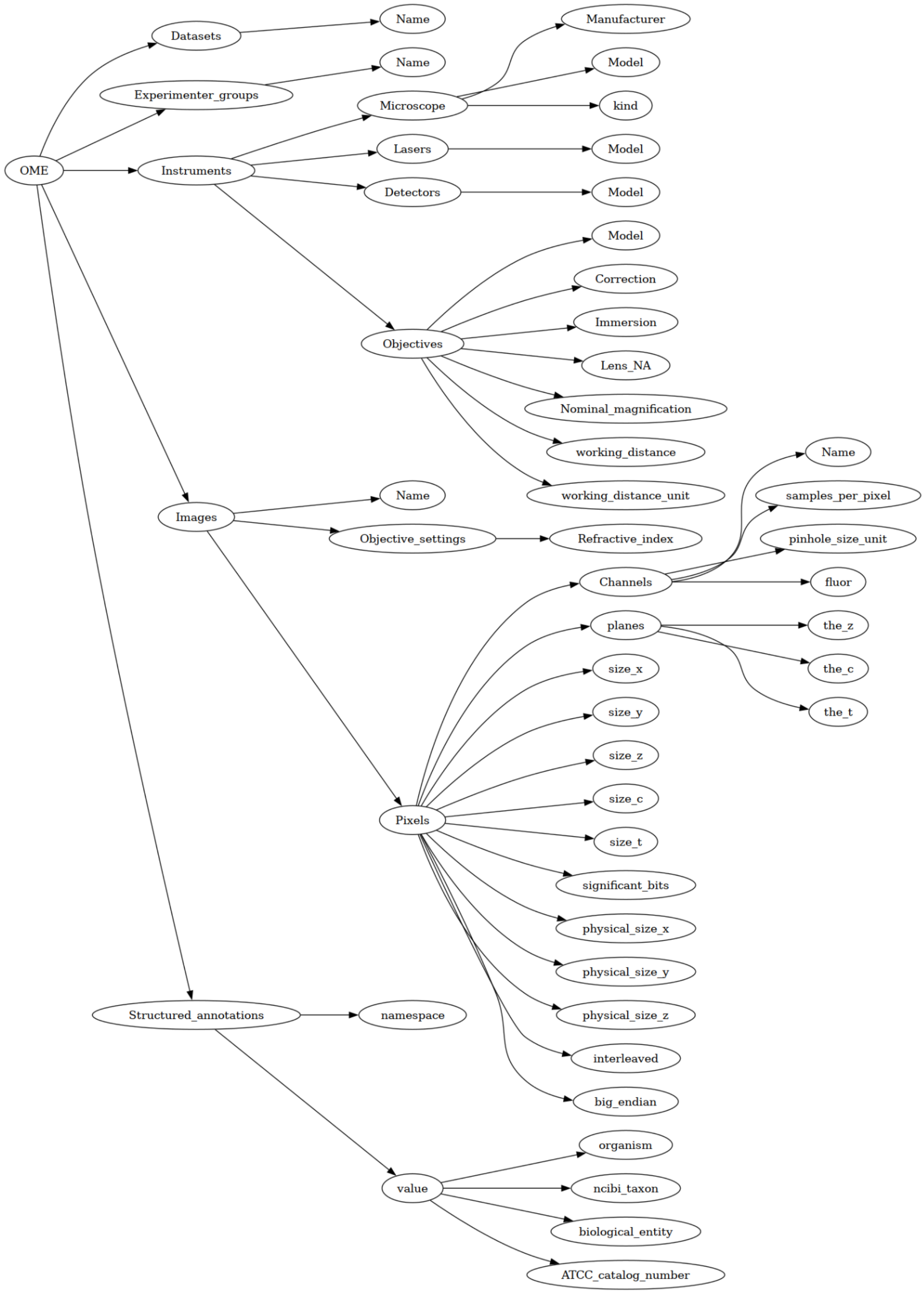


**Supplementary Figure 1 | Architecture of the image standardized metadata.**

*Tree map of how each image metadata are structured and accessible in the OME-XML. They are grouped into five general sections: Datasets with the name of the image study, Experimenter groups section with the name of the FBI node, Instruments section containing all the imaging equipment information for image acquisition, Images section grouping all image data information and Structured annotations section with the biosample information.*

## 3. Dimensionality Normalization

Original datasets included images with up to six dimensions (X, Y, Z, T, C, and S), depending on acquisition modality and instrument. To facilitate model training and standardized access, all images were reshaped into a 2D-equivalent format with singleton dimensions preserved for Z, C, and T. This dimensional flattening was implemented with custom routines that detected and adjusted axis ordering, which can vary widely across formats (e.g., XYZCT vs. TCZYX vs. XYCZT). XY spatial resolution was preserved, and dimensional reduction was performed only after best-focus plane selection to avoid loss of relevant structural information.

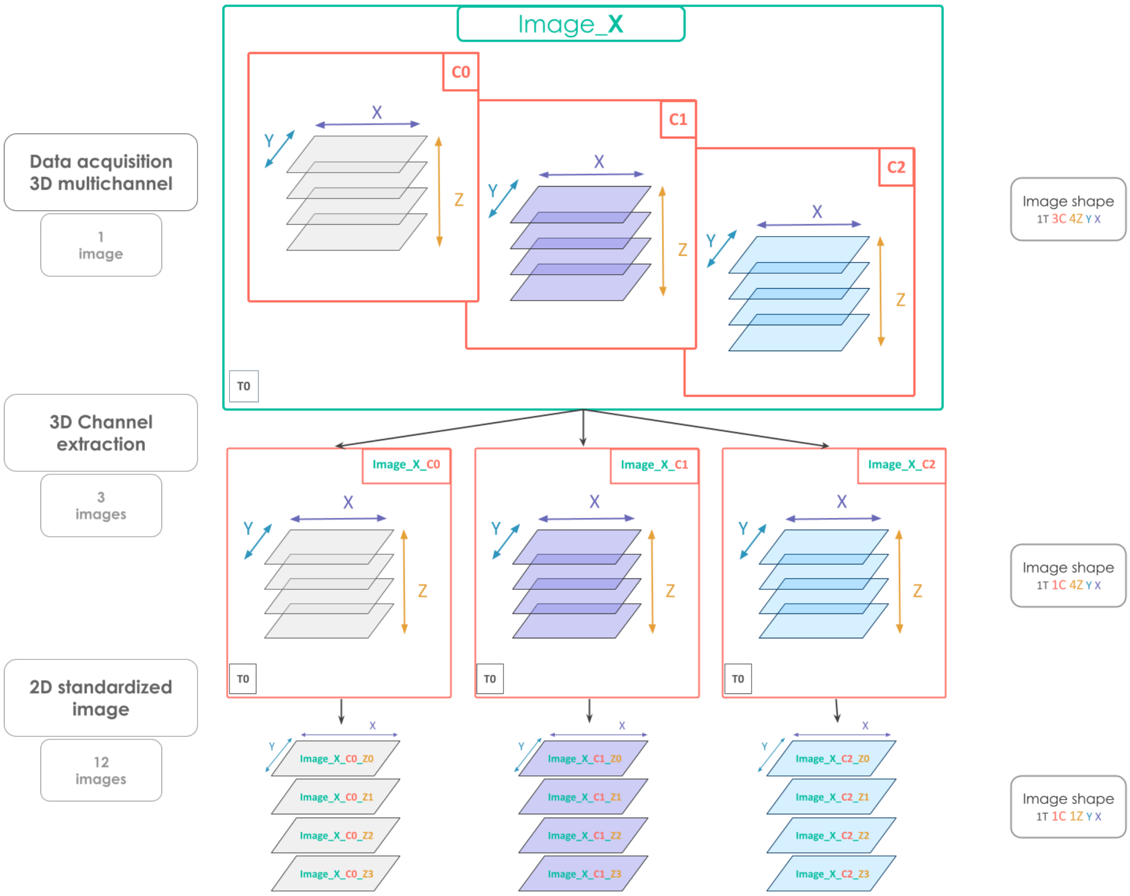


**Supplementary Figure 2 | Illustration of the Image shape normalization process.**
This scheme visually illustrates the shape normalization process applied to an example image (‘image_X’), transforming a single raw 5D image into twelve standardized images prepared for best-focus extraction prior to publication. The process involves decomposing multi-dimensional (4D or 5D), single- or multi-timepoint, multi-channel Z-Y-X images into 3D single-channel Z-Y-X volumes, which are then further split into 2D Y-X slices.

## 4. Automated Best Focal Plane Selection

As described in the *Standardized Acquisition Protocol* section, imaging was performed in 2D with limited z-stacks (typically 10–20 planes) to ensure subcellular structural resolution while controlling data volume. The outermost planes of z-stacks are frequently blurred due to optical

limitations, making reliable focus selection essential for downstream analysis.

To reflect the Light My Cells goal of generating high-quality fluorescence images from transmitted light inputs—even when the latter is blurred—we independently extracted the best-focus plane for each fluorescence-labeled organelle and for the corresponding transmitted light image. While both images show the same sample, focus optimization was performed separately per modality, allowing each to best highlight its structural content.

Because transmitted light and fluorescence microscopy differ markedly in signal characteristics (e.g., intensity range, contrast, and noise distribution), we selected focus metrics tailored to each modality. Following the large-scale benchmarking by Sun et al. (Microsc. Res. Tech. 2004)[4], which evaluated 18 algorithms across 139,000 images, we adopted Normalized Variance for transmitted light—ranked best overall for brightfield, phase contrast, and DIC imaging due to its robustness and accuracy—and Autocorrelation for fluorescence, where it was found to outperform other methods in capturing optimal focus.Both algorithms were re-implemented in Python and systematically applied to each z-stack (code available https://codeberg.org/FranceBioImaging/LightMyCellsDatabase).

The computed best-focus indices were stored for all modalities and organelles to ensure traceability. Fluorescence datasets were reduced to single best-focus planes per organelle, while full transmitted light z-stacks were retained as 2D slices with the optimal plane indexed—supporting both minimal input scenarios and depth-aware model training [Fig.3.a].

In rare cases, certain z-planes were excluded prior to analysis due to acquisition artifacts—e.g., sudden intensity shifts inconsistent with biological structure—ensuring more robust focus detection. Finally, to guarantee the biological relevance and technical quality of the selected planes, all automated focus decisions were manually validated across datasets (*see Technical Validation* section).

## 5. File Naming Harmonization

A standardized naming convention was established to encode key image attributes in the filename. Each file name includes a unique identifier corresponding to the original multi-dimensional acquisition. For transmitted light images, the modality (BF, PC, or DIC) and the z-plane index are encoded. Fluorescence filenames specify the labeled organelle (nucleus, mitochondria, tubulin, or actin), with the best-focus plane implied and not explicitly included. This scheme allows users to directly associate transmitted light inputs with their corresponding fluorescence outputs without requiring external mapping files. The naming structure was designed to be both human-readable and machine-actionable, facilitating exploration and automated use of the database.

## 6. Study-Level Organization

In line with the REMBI-compliant structure of the BioImage Archive, the full database was divided into 30 independent studies, each corresponding to a separate experimental acquisition campaign. Each study represents a unique combination of biological sample, imaging modality, and instrument configuration. Within each study, files are indexed using a continuous ascending identifier, enabling unambiguous referencing across the dataset. This organization supports traceability, reproducibility, and programmatic access, and allows the dataset to be searched or filtered by study, organelle, modality, or sample type.

## **Supplementary Notes 1:** Comprehensive Study-Level Metadata and Imaging Descriptions

The Light My Cells Database compiles 30 imaging studies, each thoroughly documented with standardized metadata and annotations to ensure reproducibility and reuse. These records include contributor details, experimental context, image examples, and technical acquisition parameters, highlighting the biological and instrumental diversity of the dataset. This structured documentation provides essential context for interpretation, supports meta-analyses, and fosters the development of robust computational models.

# Study 1

## Authors


- **Julia BONNET-GELEBART**, Centre National de la Recherche Scientifique (CNRS), Univ Rennes, IGDR - UMR 6290, F-35043 Rennes, France
julia.bonnet-gelebart@univ-rennes.fr
- **Youssef EL HABOUZ**, CNRS, Univ Rennes, IGDR - UMR 6290, F-35043 Rennes, France
youssef.elhabouz@univ-rennes.fr
- **Marc TRAMIER**, CNRS, Univ Rennes, IGDR - UMR 6290, F-35043 Rennes, France
marc.tramier@univ-rennes1.fr
- **Jacques PECREAUX**, CNRS, Univ Rennes, IGDR - UMR 6290, F-35043 Rennes, France
jacques.pecreaux@univ-rennes1.fr
- **Louis RUEL**, CNRS, Univ Rennes, IGDR - UMR 6290, F-35043 Rennes, France
louis.ruel@univ-rennes.fr


- **Role :** JBG and LR prepared the sample, JBG and LR made the acquisition, YEH prepared the images, MT and JP supervised the project.

- **France Bio-Imaging node :** Bretagne-Loire
- **France Bio-Imaging platform :** Institut Génétique & Développement de Rennes (IGDR)

## Description

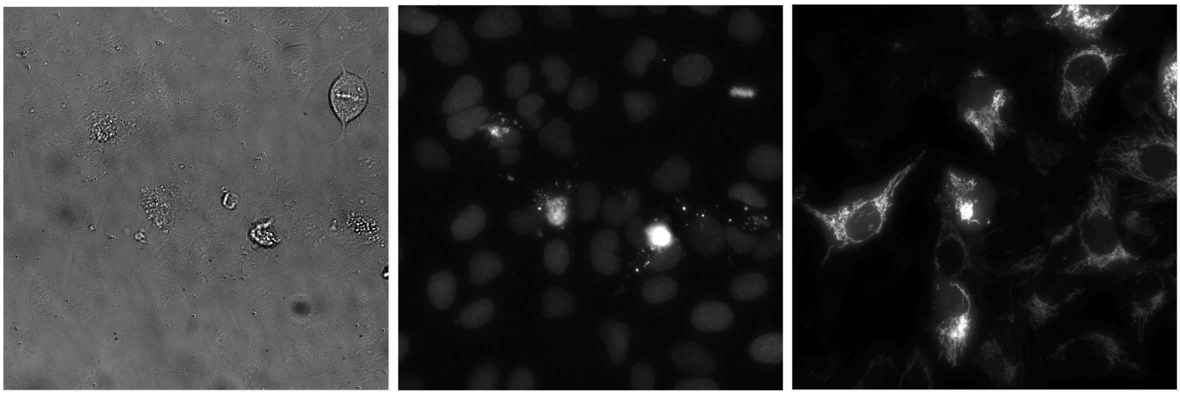

**a)** *image_1_BF_z0* **b)** *image_1_Nucleus* **c)** *image_1_Mitochondria*

**Fig.study 1:** *Example of images from Study 1.* ***a)*** *Bright-field microscopy image of human U2OS cells.* ***b)*** *Bright-field image equivalent but in fluorescence microscopy of Hoechst-labeled human U2OS nuclei.* ***c)*** *Bright-field image equivalent but in fluorescence microscopy of MitoGFP-labeled human U2OS mitochondria.*

## Study Component

- **name**: U2OS
- **file list access** : json_file tsv_file

## Biosample

- **model** : human / homo sapiens
- **NCBI** : txid9606
- **biological entity** : U2OS
- **ATCC catalog number** : HTB-96
- **description**: U-2 OS is a cell line with epithelial morphology that was derived in 1964 from a moderately differentiated sarcoma of the tibia of a 15-year-old, White, female osteosarcoma patient.

## Specimen

- **sample preparation** : cells were plated in an imaging chamber. Nucleus were labeled with Hoechst marker 15 min before acquisition. MitoGFP plasmides were transfected 48H before transfection. Plated and transfected living cells.
- **growth protocol** : cell culture medium DMEM + 5% svf

## Image Data

- **number of images** : 129 with :
    - 43 BF : image_<*img_num*>_BF_Z0.ome.tiff (where *img_num* from 0 to 42)
    - 43 Nucleus : image_<*img_num*>_Nucleus.ome.tiff (where *img_num* from 0 to 42)
    - 43 Mitochondria : image_<*img_num*>_Mitochondria.ome.tiff (where *img_num* from 0 to 42)
- **z planes :**
    - number : 1
    - BF z focus : 0
    - Nucleus z focus : 0
    - Mitochondria z focus : 0
- **channels** :
    - nucleus :
        - marker : Hoechst
        - excitation light : 390/22 nm
        - emission light : 445/20 nm
    - mitochondria :
        - marker : mitoGFP
        - excitation light : 475/34 nm
        - emission light : 536/40 nm

- **dimension order** : TCZYX
- **images size** : 1, 1, 1, 2048, 2048
- **physical size x** : 0.10317460318938645 µm
- **physical size y** : 0.10317460318938645 µm
- **physical size z** : 1.0 µm
- **type** : uint16
- **format** : ome.tiff

## Image Acquisition

- **imaging method** : Bright Field (BF), Fluorescence
- **imaging instrument** : Inverted Leica DMi8 stand, equipped with environmental control (37°C and $CO_2$) controlled by Inscoper software.
- **pixel size** : 100 nm
- **light source** : Lamp
- **detector model** : Hamamatsu Orca Flash 4.0
- **objective** :
    - **model** : Leica
    - **immersion** : dry (air)
    - **lens numerical aperture (NA)** : 0.8
    - **nominal magnification** : 63x
    - **working distance** : 0.26 mm
    - **refractive index** : 1.0002

## Raw Image Notes

- **samples per pixel** : 1
- **z planes number** : 1
- **dimension order** : TCZYX
- **images size** : 1, 3, 1, 2048, 2048
- **channel order** : Single channel, order n.a.
- **type** : uint16
- **format** : ome.tif

# Study 2

## Authors


- **Aude MORET**, INSERM UMRS 938, CRSA, Paris, France
  aude.moret@inserm.fr
- **Romain MORICHON**, Sorbonne Université, CRSA, Cytométrie Imagerie Saint-Antoine, Paris, France
  romain.morichon@sorbonne-universite.fr


- **Role :** AM prepared the sample, RM made the acquisition.

- **France Bio-Imaging node :** Paris Centre
- **France Bio-Imaging platform :** Centre de Recherche Saint-Antoine (CRSA)

## Description

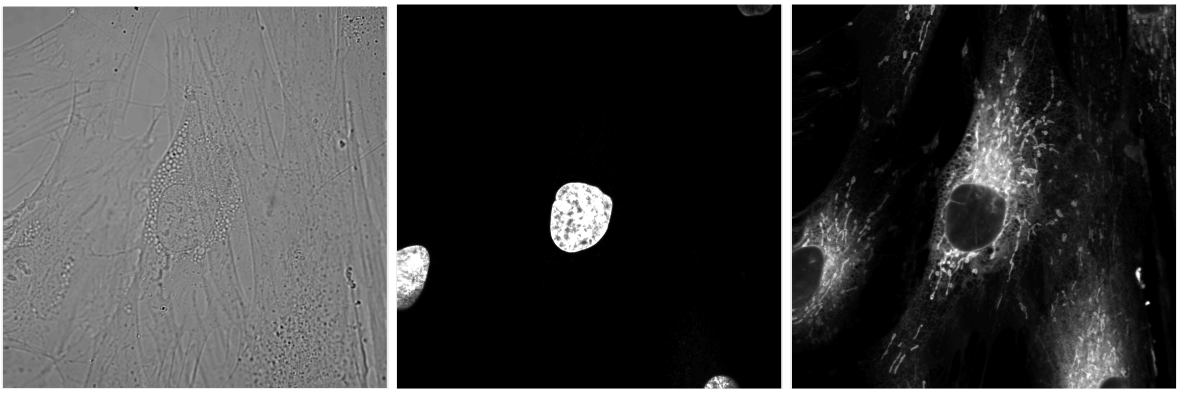

**a)** *image_43_DIC_z0* **b)** *image_43_Nucleus* **c)** *image_43_Mitochondria*

**Fig.study 2:** *Example of images from Study 2.* ***a)*** *Differential interference contrast (DIC) microscopy image of human* Fibroblast. ***b)*** *DIC image equivalent but in fluorescence microscopy of Hoechst-labeled human Fibroblast nuclei.* ***c)*** *DIC image equivalent but in fluorescence microscopy of MitoTracker-labeled human Fibroblast mitochondria.*

## Study Component

- **name**: Fibroblast CRSA
- **file list access** : json_file tsv_file

## Biosample

- **model** : human / homo sapiens
- **NCBI** : txid9606
- **biological entity** : Fibroblast from Dermis
- **ATCC catalog number** : NHDF-d ref : C-12352 PROMOCELL : normal fibroblasts.
- **description**: Contains normal dermal adult fibroblasts and dermal fibroblasts derived from primary cells of 2 Shwachman-Diamond patients.

## Specimen

- **sample preparation** : Cells were plated in clear plate and incubated with probes after adhesion. Cells were plated in µ-Plate 24 Well Black ID 14mm ibiTreat. Mitotracker (Invitrogen M7514, concentration finale = 500nM) was added with hoechst 33342 (final Concentration = 1 µg/mL).
- **growth protocol** : DMEM 1G/L Glucose +Pyruvate 10% SVF 1%PS 1%L-Glu

## Image Data

- **number of images** : 145 with :
  - 125 DIC : image_<*n_img*>_DIC_z<*n_z*>.ome.tiff (where *n_img* from 43 to 52 and *n_z from 0 to 17)*
  - 10 Nucleus : image_<*n_img*>_Nucleus.ome.tiff (where *n_img* from 43 to 52)

    - 10 Mitochondria : image_<*n_img*>_Mitochondria.ome.tiff (where *n_img* from 43 to 52)
- **z planes :**
    - number : 1
    - DIC z focus : 0, 1, 6, 7, 8, 9 or 11 depending on the image
    - Nucleus z focus : 2, 3, 4, 5 or 6 depending on the image
    - Mitochondria z focus : 5, 6, 7, 8 or 9 depending on the image
- **channels** :
    - nucleus :
        - marker : Hoechst
        - excitation light : 430.0 nm
        - emission light : 470.0 nm
    - mitochondria :
        - marker : MitoTracker
        - excitation light :  650.0 nm
        - emission light :  750.0 nm

- **dimension order** : TCZYX
- **images size** : 1, 1, 1, 1024, 1024
- **physical size x** : 0.103580094900373 µm
- **physical size y** : 0.103580094900373 µm
- **physical size z** : 0.47 µm
- **type** : uint16
- **format** : ome.tiff

## Image Acquisition

- **imaging method** : Differential interference contrast (DIC), Fluorescence
- **imaging instrument** : Confocal Olympus Fluoview FV3000, Plan Apo 60X/1,35 oil, 1024*1024 pixels with zoom 2
- **pixel size** : 103 nm
- **light source** : Diode OBIS 405nm 50mW (wavelength: 405.0 nm), Diode OBIS 640nm 40mW (wavelength: 640.0 nm)
- **detector model :** High sensitivity spectral detector (HSD)
- **objective** :
    - **model** : UPLXAPO 60XO
    - **immersion** : oil
    - **lens numerical aperture (NA)** : 1.42
    - **nominal magnification** : 60x
    - **working distance** : 0.15 mm
    - **refractive_index** : 1.518

## Raw Image Notes

- **samples per pixel** : 1
- **z planes number** : from 9 to 17
- **dimension order** : TCZYX
- **images size** : 1, 3, 9 to 17, 1024, 1024
- **channel order** : Hoechst, MitoTracker, DIC
- **type** : uint16
- **format** : .oir

# Study 3

## Authors


- **Daniel STOCKHOLM**, PSL Research University, EPHE, 75014 Paris, France. Genethon, Plateforme ImCy, Evry, France
  stockho@genethon.fr
- **Abbass JABER**, Genethon, Université Paris-Saclay, Univ Evry, Inserm, Integrare research unit UMR_S951, 91000, Évry-Courcouronnes, France
  ajaber@genethon.fr


- **Role :** AJ prepared the sample, AJ and DS made the acquisition.

- **France Bio-Imaging node :** Ile de France Sud
- **France Bio-Imaging platform :** Genethon's Imaging Cytometry platform (ImCy)

## Description

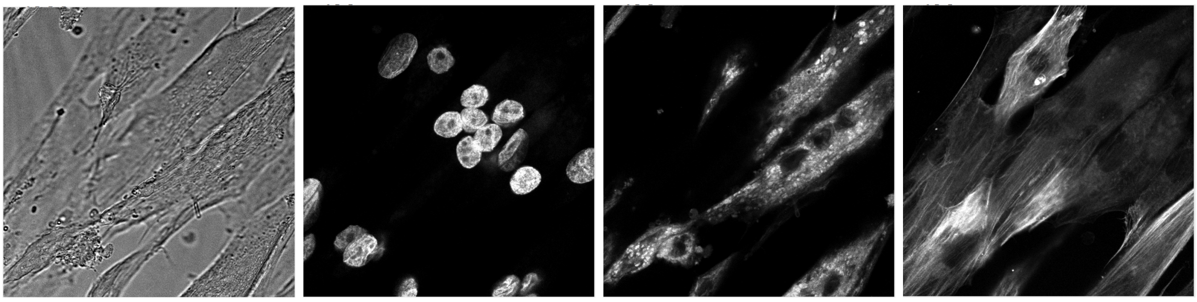

**a)** *image_60_BF_z0* **b)** *image_60_Nucleus* **c)** *image_60_Mitochondria* **d)** *image_60_Actin*

**Fig.study 3:** *Example of images from Study 3.* ***a)*** *BF microscopy image of human Myoblast.* ***b)*** *BF image equivalent but in fluorescence microscopy of Hoechst-labeled human Myoblast nuclei.* ***c)*** *BF image equivalent but in fluorescence microscopy of MitoTracker-labeled human Myoblast mitochondria.* ***d)*** *BF image equivalent but in fluorescence microscopy of Phalloidin-labeled human Myoblast actin.*

## Study Component

- **name**: Myoblast Genethon
- **file list access** : json_file tsv_file
- download study 3 images : S-BIAD1047#Study3

## Biosample

- **model** : human / homo sapiens
- **NCBI** : txid9606
- **biological entity** : Immortalized Myoblast - C25 cell line
- **ATCC catalog number** : Not referenced
- **description**: Myoblasts are isolated from a healthy patient and immortalized at the Myoline platform at the Institute of Myology (Paris)

## Specimen

- **sample preparation** : Cells were plated in a 35mm glass bottom dish (Ibidi, 81156). Mitochondria were stained with MitoTracker (Invitrogen M7514, final concentration = 500nM) diluted in HBSS buffer (with Calcium and Magnesium) for 30 min at 37°C and the DNA was stained with Hoechst 33342 (final concentration 1µg/ml) for 30 min at 37°C. Phalloïdine (Rhodamin) was added (dilution 1/500 in HBSS) and let for 5mn. Fixation in 4% PFA.
- **growth protocol** : myoblasts were cultured in skeletal muscle growth medium (Promocell, C-23060) and kept at less 60% confluency.

## Image Data

- **number of images** : 125 with :
  - 103 BF : image_<*n_img*>_BF_z<*n_z*>.ome.tiff (where *n_img* from 53 to 60 and *n_z from 0 to 20*)

- 8 nucleus : image_<*n_img*>_Nucleus.ome.tiff (where *n_img* from 53 to 60)
    - 7 mitochondria :  image_<*n_img*>_Mitochondria.ome.tiff (where *n_img* from 53 to 60)
    - 7 actin :  image_<*n_img*>_Actin.ome.tiff (where *n_img* from 53 to 60)
- **z planes :**
    - number : 1
    - BF z focus : 0, 4, 8 or 19 depending on the image
    - Nucleus z focus : 0, 4, 7, 12 or 19 depending on the image
    - Mitochondria z focus : 0, 13, 15, 17 or 19 depending on the image
    - Actin z focus : 0, 5, 9, 17 or 19 depending on the image
- **channels** :
    - nucleus :
        - marker : Hoechst
        - excitation light : 430.0 nm
        - emission light : 470.0 nm
    - mitochondria :
        - marker : MitoTracker
        - excitation light : 650.0 nm
        - emission light : 750.0 nm
    - actin:
        - marker : Phalloidin
        - excitation light : 650.0 nm
        - emission light : 750.0 nm

- **dimension order** : TCZYX
- **images size** : 1, 1, 1, 2048, 2048
- **physical size x** : 0.05151060087933561 µm
- **physical size y** : 0.05151060087933561 µm
- **physical size z** : 0.445185 µm
- **type** : uint16
- **format** : ome.tiff

## Image Acquisition

- **imaging method** : Bright Field (BF), Fluorescence
- **imaging instrument** : Leica - TCS SP8 STED 3X
- **pixel size** : 52 nm
- **light source** :  Diode (wavelength: 405.0 nm), OPSL (wavelength: 488.0 nm), OPSL (wavelength: 532.0 nm), OPSL 552 (wavelength: 552.0 nm), OPSL 635 (wavelength: 635.0 nm), STED (wavelength: 775.0 nm)
- **detector model** : Leica HyD
- **objective** :
    - **model** : HC PL APO CS2 63x
    - **immersion** : oil
    - **lens numerical aperture (NA)** : 1.4
    - **nominal magnification** : 63x
    - **working distance** : 0.15 mm
    - **refractive index** :  1.518

## Raw Image Notes

- **samples per pixel** : 1
- **z planes number** : 20
- **dimension order** : TCZYX
- **images size** : 1, 4, 20, 2048, 2048
- **channel order** : Hoechst, DIC, Phalloidine, MitoTracker
- **type** : uint16
- **format** : .lif

# Study 4

## Authors

- **Tudor MANOLIU**, Gustave Roussy Cancer Campus, PFIC, UAR AMMICa, 94805 Villejuif, France
tudor.manoliu@gustaveroussy.fr

- **Role :** TM prepared the sample and made the acquisition.

- **France Bio-Imaging node :** Ile de France Sud
- **France Bio-Imaging platform :** Plateforme d'imagerie et cytometrie (PFIC)

## Description

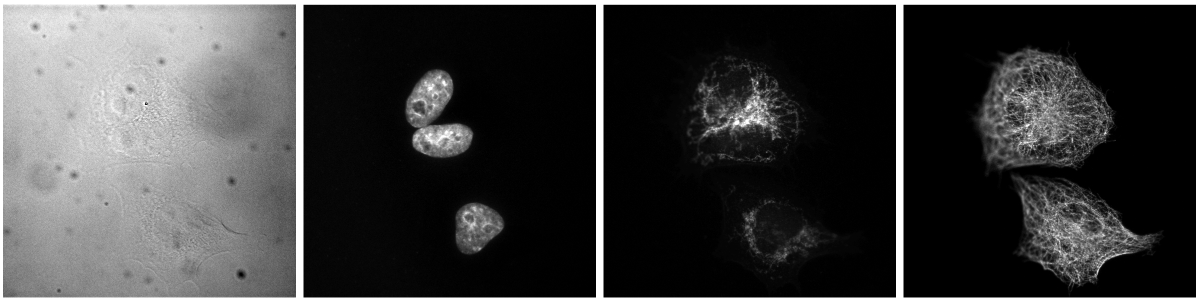

**a)** *image_85_DIC_z0* **b)** *image_85_Nucleus* **c)** *image_85_Mitochondria* **d)** *image_85_Tubulin*

**Fig.study 4:** *Example of images from Study 4.* ***a)*** *DIC microscopy image of human HELA cells.* ***b)*** *DIC image equivalent but in fluorescence microscopy of Hoechst-labeled human HELA cells nuclei.* ***c)*** *DIC image equivalent but in fluorescence microscopy of MitoTracker-labeled human HELA cells mitochondria.* ***d)*** *DIC image equivalent but in fluorescence microscopy of SIR-Tubulin-labeled human HELA cells tubulin.*

## Study Component

- **name**: HeLa Roussy
- **file list access** : json_file tsv_file

## Biosample

- **model** : human / homo sapiens
- **NCBI** : txid9606
- **biological entity** : HELA cells
- **ATCC catalog number** : CCL-2
- **description**: HeLa cells were stained to analyze the nuclear DNA, mitochondria and the cytoskeleton using Hoechst, MitoTracker and SIR-Tubulin. The cells were cultured on 35 mm diameter glass bottom petri-dishes (ibidi) until they reached 80% confluence. Staining Procedure: Hoechst dye was added directly to the culture medium at a final concentration of 1ug/ml. The cells were incubated at 37°C for 10 minutes. MitoTracker was added to the medium at a concentration of 152nm. Cells were incubated 1h at 37°C. SIR-Tubulin was added at a final concentration of 100nm for 1H at 37°C. The cells were rinsed gently with warm 35°C PBS to remove excess dye and were then imaged immediately using a Leica Videomicroscope DMI6000.Imaging was acquired with a Leica HC PL APO OIL 63X NA 1.40, WD 140 um objective.

## Specimen

- **sample preparation** : Cells were plated in Ibidi 35mm diam. glass bottom dish #1,5 in DMEM.
- **growth protocol** : Cell culture medium DMEM + 10% FBS + 1% pen/strep

## Image Data

- **number of images** : 408 with :
    - 315 DIC : image_<*n_img*>_DIC_z<*n_z*>.ome.tiff (where *n_img* from 61 to 91 and *n_z from 0 to 30*)
    - 31 Nucleus : image_<*n_img*>_Nucleus.ome.tiff (where *n_img* from 61 to 91)

    - 31 Mitochondria : image_<*n_img*>_Mitochondria.ome.tiff  (where *n_img* from 61 to 91)
    - 31 Tubulin : image_<*n_img*>_Tubulin.ome.tiff (where *n_img* from 61 to 91)
- **z planes :**
    - number : 1
    - DIC z focus : 0, 1, 2, 3, 5, 6, 7, 8, 9, 16, 17 or 27 depending on the image
    - Nucleus z focus : 0, 6, 7, 8, 9, 10, 11, 12, 18, 19 or 27 depending on the image
    - Mitochondria z focus : 0, 7, 8, 9, or 27 depending on the image
    - Tubulin z focus :  0, 7, 8, 10, or 18 depending on the image
- **channels** :
    - nucleus :
        - marker : Hoechst (1ug/ml)
        - excitation light : 350/50 nm
        - emission light : 460/50 nm
    - mitochondria :
        - marker : MitoTracker (Invitrogen, Incubation 1h, 37°C, 150nM)_Ax488
        - excitation light : 480/40 nm
        - emission light : 527/30 nm
    - tubulin :
        - marker : SIR-Tubulin (Invitrogen, Incubation 1h,37°C, 100nM)_Ax555
        - excitation light : 546/10 nm
        - emission light : 585/40 nm

- **dimension order** : TCZYX
- **images size** :  1, 1, 1, 966, 1296
- **physical size x** : 0.05952381 µm
- **physical size y** : 0.05952381 µm
- **physical size z** : 0.21532 µm
- **type** : uint16
- **format** : ome.tiff

## Image Acquisition

- **imaging method** : DIC, Fluorescence
- **imaging instrument** : Inverted Leica DMI8 video-microscope temperature and gas controlled.
- **pixel size** : 60 nm
- **light source** : Mercury HXP lamp (OSRAM) 120W 85V/45C VIS
- **detector model** : DFC 3000G - Leica CCD
- **objective** :
    - **model** :  Leica HC PL APO
    - **immersion** : Oil
    - **lens numerical aperture (NA)** : 1.4
    - **nominal magnification** : 63X
    - **working distance** :  0.140 mm
    - **refractive index** : 1.518

## Raw Image Notes

- **samples per pixel** : 1
- **z planes number** : from 1 to 31
- **dimension order** : TCZYX
- **images size** : 1, 4, 1 to 31, 966, 1296
- **channel order** : SIR-Tubulin, MitoTracker, Hoechst,  DIC
- **type** : uint16
- **format** : .lif

# Study 5

## Authors


- **Elsa CASTELLANI**, Light Microscopy Imaging facility Marseille, IBDM, CNRS UMR 7288, France.
  elsa.castellani@univ-amu.fr
- **Qiyan MAO**, team Schnorrer, IBDM, CNRS UMR7288, France
  qiyan.mao@univ-amu.fr


- **Role :** Qiyan Mao prepared the sample and Elsa Castellani made the acquisition.

- **France Bio-Imaging node :** Marseille
- **France Bio-Imaging platform :** Institut de Biologie du Développement de Marseille (IBDM)

## Description

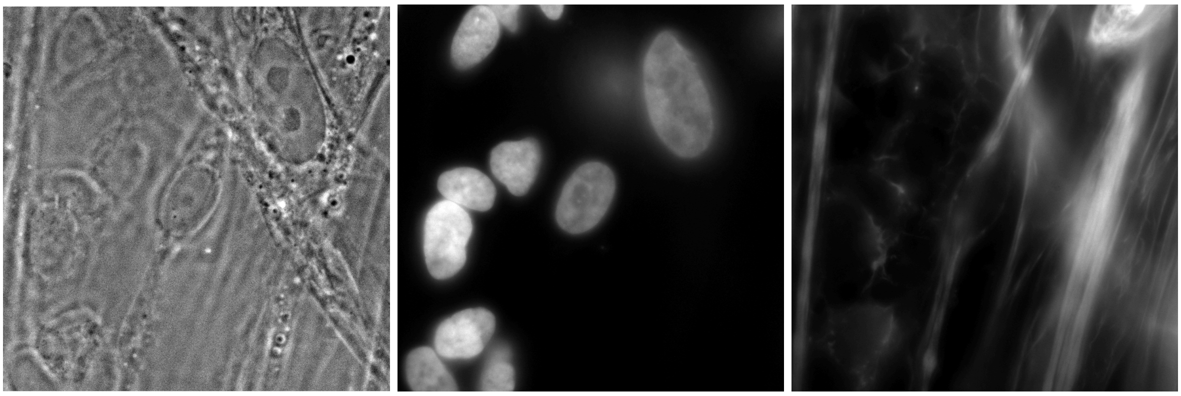

**a)** *image_100_PC_z10* **b)** *image_100_Nucleus* **c)** *image_100_Actin*

**Fig.study 5:** *Example of images from Study 5.* ***a)*** *Phase contrast microscopy image of human muscle fiber.* ***b)*** *Phase contrast image equivalent but in fluorescence microscopy of DAPI-labeled human muscle fiber nuclei.* ***c)*** *Phase contrast image equivalent but in fluorescence microscopy of Atto647-Myofibrils-labeled human muscle fiber mitochondria.*

## Study Component

- **name** : Human muscle AMU 2
- **file list access** : json_file tsv_file

## Biosample

- **model** : human / homo sapiens
- **NCBI** : txid9606
- **biological entity** : muscle fiber
- **ATCC catalog number** : Not referenced
- **description**: n.a.

## Specimen

- **sample preparation** : Cells in 35mm petri dish, with glass bottom #1,5, 1e antibody: 4oC, overnight, mouse-anti-YAP: 1:150, rabbit-anti-Titin: 1:150, 2nd antibody: room temperature, 2hrs, Alexa488-anti-mouse: 1:500, Alexa568-anti-rabbit: 1:500, Atto647-phalloidin: 1:500, DAPI is in the vectashield mounting media. Fixation with 4% PFA.
- **growth protocol** : Skeletal muscle progenitors are expanded two days in SKGM-2 growth media on Matrigel coated culture dishes, then differentiated into myofibers in KCTiP media for one to two weeks.

## Image Data

- **number of images** : 140 with :

    - 120 PC : image_<*n_img*>_PC_z<*n_z*>.ome.tiff (where *n_img* from 92 to 101 and *n_z from 0 to 12*)
    - 10 Nucleus : image_<*n_img*>_Nucleus.ome.tiff (where *n_img* from 92 to 101)
    - 10 Actin : image_<*n_img*>_Actin.ome.tiff (where *n_img* from 92 to 101)
- **z planes :**
    - number : 1
    - PC z focus : 2, 3, 4 or 6 depending on the image
    - Nucleus z focus : 1, 3 or 5 depending on the image
    - Actin z focus : 2, 3, 4 or 6 depending on the image
- **channels** :
    - nucleus :
        - marker : DAPI
        - excitation light : 375 nm
        - emission light : 450-500 nm
    - actin :
        - marker : Atto647-Myofibrils
        - excitation light : 620-660 nm
        - emission light : 672-712nm

- **dimension order** : TCZYX
- **images size** : 1, 1, 1, 1300, 1624
- **physical size x** : 0.065 µm
- **physical size y** : 0.065 µm
- **physical size z** : 1.0 µm
- **type** : uint16
- **format** : ome.tiff

## Image Acquisition

- **imaging method** : Phase Contrast (PC), Fluorescence
- **imaging instrument** : Zeiss AxioObserverZ1, EC Plan Neofluar Ph3
- **pixel size** : 65 nm
- **light source** : Mercure Lamp HBO 100W
- **detector model** : Hamamatsu HDCamC11440-42U
- **objective** :
    - **model** : Hamamatsu OrcaFlash4LT
    - **immersion** : Oil
    - **lens numerical aperture (NA)** : 1,3
    - **nominal magnification** : 100x
    - **working distance** : 0.2 mm
    - **refractive index** : 1.518

## Raw Image Notes

- **samples per pixel** : 1
- **z planes number** : 12
- **dimension order** : TCZYX
- **images size** : 1, 3, 12, 1300, 1624
- **channel order** : DAPI, Atto647-Myofibrils, PC
- **type** : uint16
- **format** : ome.tif

## Study 6

### Authors


- **Elsa CASTELLANI**, Light Microscopy Imaging facility Marseille, IBDM CNRS UMR 7288, France.
- elsa.castellani@univ-amu.fr
- **Qiyan MAO**, team Schnorrer, IBDM, CNRS UMR7288, France
  qiyan.mao@univ-amu.fr


- **Role :** QM prepared the sample and EC made the acquisition.

- **France Bio-Imaging node :** Marseille
- **France Bio-Imaging platform :** Institut de Biologie du Développement de Marseille (IBDM)

### Description

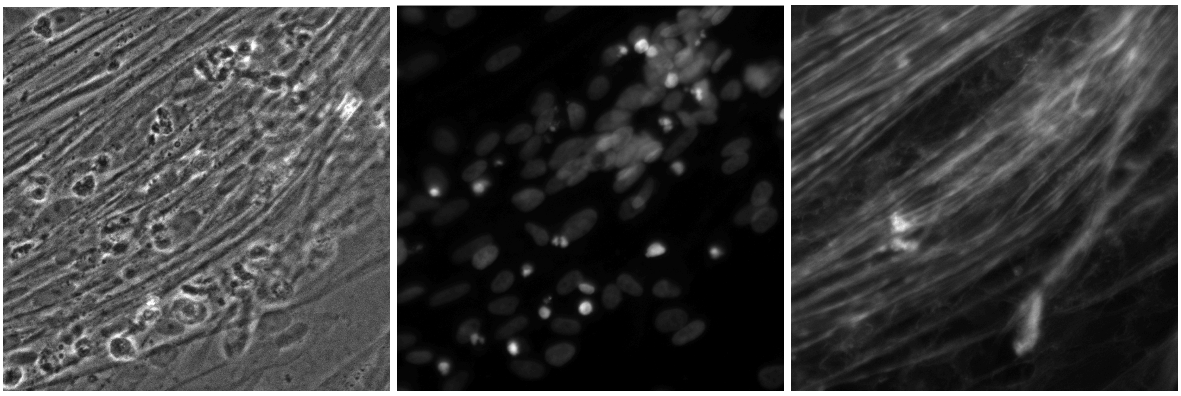

**a)** *image_111_PC_z3* **b)** *image_111_Nucleus* **c)** *image_111_Actin*

**Fig.study 6:** *Example of images from Study 6.* ***a)*** *Phase contrast microscopy image of human muscle fiber.* ***b)*** *Phase contrast image equivalent but in fluorescence microscopy of DAPI-labeled human muscle fiber nuclei.* ***c)*** *Phase contrast image equivalent but in fluorescence microscopy of Atto647-Myofibrils-labeled human muscle fiber mitochondria.*

### Study Component

- **name**: Human muscle AMU
- **file list access** : json_file tsv_file

### Biosample

- **model** : human / homo sapiens
- **NCBI** : txid9606
- **biological entity** : muscle fiber
- **ATCC catalog number** : Not referenced
- **description**: n.a.

### Specimen

- **sample preparation** : Cells in 35mm petri dish, with glass bottom #1,5, 1e antibody: 4oC, overnight, mouse-anti-YAP: 1:150, rabbit-anti-Titin: 1:150, 2nd antibody: room temperature, 2hrs, Alexa488-anti-mouse: 1:500, Alexa568-anti-rabbit: 1:500, Atto647-phalloidin: 1:500, DAPI is in the vectashield mounting media. Fixation with 4% PFA.
- **growth protocol** : Skeletal muscle progenitors are expanded two days in SKGM-2 growth media on Matrigel coated culture dishes, then differentiated into myofibers in KCTiP media for one to two weeks.

### Image Data

- **number of images** : 130 with :

    - 110 PC : image_<*n_img*>_PC_z<*n_z*>.ome.tiff (where *n_img* from 102 to 111 and *n_z from 0 to 12*)
    - 10 Nucleus : image_<*n_img*>_Nucleus.ome.tiff  (where *n_img* from 102 to 111)
    - 10 Actin : image_<*n_img*>_Actin.ome.tiff  (where *n_img* from 102 to 111)
- **z planes :**
    - number : 1
    - PC z focus : 4 or 5 depending on the image
    - Nucleus z focus :  3, 4 or 5 depending on the image
    - Actin z focus : 3, 4 or 5 depending on the image
- **channels** :
    - nucleus :
        - marker : DAPI
        - excitation light : 350/50 nm
        - emission light : 460/50 nm
    - actin :
        - marker : Atto647-Myofibrils
        - excitation light : 620-660 nm
        - emission light : 672-712 nm

- **dimension order** : TCZYX
- **images size** : 1, 1, 1, 1300, 1624
- **physical size x** : 0.1625 µm
- **physical size y** : 0.1625 µm
- **physical size z** : 1.0 µm
- **type** : uint16
- **format** : ome.tiff

## Image Acquisition

- **imaging method** : PC, Fluorescence
- **imaging instrument** : Zeiss AxioObserverZ1, LD PlnN, 6,5µm
- **pixel size** : 162 nm
- **light source** : Mercure Lamp HBO 100W
- **detector model** : Hamamatsu HDCamC11440-42U
- **objective** :
    - **model** : Hamamatsu OrcaFlash4LT
    - **immersion** : Air
    - **lens numerical aperture (NA)** : 0.6
    - **nominal magnification** : 40xLD
    - **working distance** : 2.970 mm
    - **refractive index** :  1 .0

## Raw Image Notes

- **samples per pixel** : 1
- **z planes number** : 12
- **dimension order** : TCZYX
- **images size** : 1, 3, 12, 1300, 1624
- **channel order** : DAPI, Atto647-Myofibrils, PC
- **type** : uint16
- **format** : .czi

# Study 7

## Authors


- **Elsa CASTELLANI**, Light Microscopy Imaging facility Marseille, IBDM CNRS UMR 7288, France.
  elsa.castellani@univ-amu.fr
- **Laura Ruiz**, eqp Delacour, IBDM CNRS UMR7288, France
  laura.ruiz@univ-amu.fr


- **Role :** LR prepared the sample and EC made the acquisition.

- **France Bio-Imaging node :** Marseille
- **France Bio-Imaging platform :** Institut de Biologie du Développement de Marseille (IBDM)

## Description

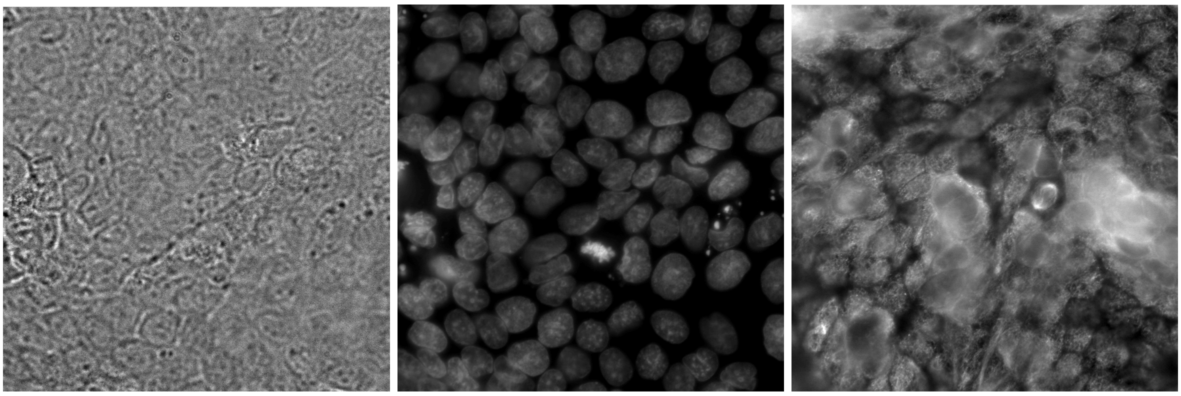

**a)** *image_112_DIC_z12* **b)** *image_112_Nucleus* **c)** *image_112_Tubulin*

**Fig.study 7:** *Example of images from Study 7.* ***a)*** *DIC microscopy image of human cancer cell from colon.* ***b)*** *DIC image equivalent but in fluorescence microscopy of DAPI-labeled human nuclei.* ***c)*** *DIC image equivalent but in fluorescence microscopy of alpha-tubulin in human cancer cell.*

## Study Component

- **name** : Human intestine AMU
- **file list access** : json_file tsv_file

## Biosample

- **model** : human / homo sapiens
- **NCBI** : txid9606
- **biological entity** : colon cells
- **ATCC catalog number** : *HTB-37™*
- **description**: Caco2 are epithelial cells isolated from colon tissue.

## Specimen

- **sample preparation** : Cells were plated in glass bottom (#1,5), Human intestine cell DAPI for nuclei, 1/1000 diluted in PBS for 10 min, Alexa488:NuMa, Ac1er PBD++2%Dk serum NuMa anti rb 1/200 then a488 1:200 for 2h, Alexa568:Alpha Tubulin, Ac1er PBD++2%Dk serum alpha tubulin anti mouse 1/200 then a568 1:200 for 2h.
- **growth protocol** : Cells where plate on glass cover slip for two weeks until the maturation of the cells.

## Image Data

- **number of images** : 150 with :
  - 130 DIC : image_<*n_img*>_DIC_z<*n_z*>.ome.tiff (where *n_img* from 112 to 121 and *n_z from 0 to 13*)
  - 10 Nucleus : image_<*n_img*>_Nucleus.ome.tiff (where *n_img* from 112 to 121)

    - 10 Tubulin : image_<*n_img*>_Actin.ome.tiff (where *n_img* from 112 to 121)
- **z planes :**
    - number : 1
    - DIC z focus : 8, 9, 10 or 11 depending on the image
    - Nucleus z focus : 1, 2, 3, 4 or 5 depending on the image
    - Tubulin z focus : 4 or 5 depending on the image
- **channels** :
    - nucleus :
        - marker : DAPI
        - excitation light : 350/50 nm
        - emission light : 460/50 nm
    - tubulin :
        - marker : Alexa568-Alpha tubulin Abcam (ref ab18251)
        - excitation light : 546/10 nm
        - emission light : 585/40 nm

- **dimension order** : TCZYX
- **images size** : 1, 1, 1, 1300, 1624
- **physical size x** : 0.10317460317460317 µm
- **physical size y** : 0.10317460317460317 µm
- **physical size z** : 1.0 µm
- **type** : uint16
- **format** : ome.tiff

## Image Acquisition

- **imaging method** : DIC, Fluorescence
- **imaging instrument** : Zeiss AxioObserverZ1, Plan Apochromat, 6,5µm
- **pixel size** : 103 nm
- **light source** : Mercure Lamp HBO 100W
- **detector model** : Hamamatsu HDCamC11440-42U
- **objective** :
    - **model** : Hamamatsu OrcaFlash4LT
    - **immersion** : Oil
    - **lens numerical aperture (NA)** : 1.4
    - **nominal magnification** : 63x
    - **working distance** : 1.93 mm
    - **refractive index** : 1.518

## Raw Image Notes

- **samples per pixel** : 1
- **z planes number** : 12
- **dimension order** : TCZYX
- **images size** : 1, 3, 12, 1300, 1624
- **channel order** : DAPI, Alexa568-Alpha, PC
- **type** : uint16
- **format** : .czi

# Study 8

## Authors


- **Stéphanie BOSCH**, CNRS, Univ Toulouse 3, TRI-LITC, CBI- FR 3743, F-31062 Toulouse cedex 9, France
stephanie.bosch@univ-tlse3.fr
- **Maëlle CARRAZ**, IRD-CNRS, Univ Toulouse 3, PharmaDev UMR 152 and MCD UMR 5077, F-31062 Toulouse cedex 9, France
maelle.carraz@ird.fr


- **Role :** MC prepared samples (cells and labeling), SB made the microscopy acquisitions.

- **France BioImaging node :** Toulouse
- **France BioImaging platform :** Centre de Biologie Intégrative (CBI)

## Description

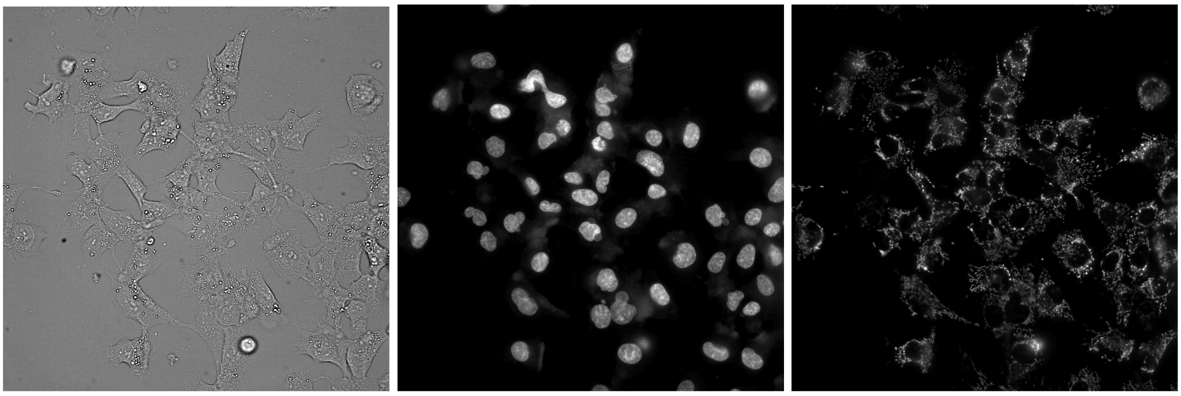

**a)** *image_122_BF_z2* **b)** *image_122_Nucleus* **c)** *image_122_Mitochondria*

**Fig.study 8:** *Example of images from Study 8.* ***a)*** *BF microscopy image of Hep3B human liver cancer cells.* ***b)*** *BF image equivalent but in fluorescence microscopy of Hoechst- labeled human liver cells nuclei.* ***c)*** *BF image equivalent but in fluorescence microscopy of Mito Red- labeled human liver cells mitochondria.*

## Study Component

- **name** : Human Liver Carcinoma
- **file list access** : json_file tsv_file

## Biosample

- **model** : human / homo sapiens
- **NCBI** : txid9606
- **biological entity** : Hep3B human liver carcinoma
- **ATCC catalog number** : Hep 3B2.1-7, HB-8064
- **description**: the cell line exhibits an epithelial morphology that was isolated from liver tissue derived from an 8-year-old, Black youth with liver cancer. This cell line contains an integrated hepatitis B virus genome.

## Specimen

- **sample preparation** : Cells were plated in glass bottom micro-dish (35 mm) ibidi® in DMEM complete medium at 250,000 cells per plate, for 24h. Medium was then replaced by opti-MEM (without serum nor phenol red). Nuclei were labeled with 0.5µL/mL Hoechst 33342, 10 min. Mito Red was then added at a final concentration of 10 nM during 30 min at 37°C to stain mitochondria.
- **growth protocol** : Hep3B cells are adherent human cells cultivated in complete DMEM medium containing 10% FBS, 1% L-glutamine, sodium pyruvate and non-essential amino acids. They are trypsinized with trypsin-EDTA 0.05% every 3 days for passages.

## Image Data

- **number of images** : 168 with :
    - 152 BF : image_<*img_num*>_BF_z<*z_num*>.ome.tiff (where *num_img* from 122 to 129 and *z_num from 0 to 18*)
    - 8 Nucleus : image_<*img_num*>_Nucleus.ome.tiff (where *img_num* from 122 to 129)
    - 8 Mitochondria : image_<*img_num*>_Mitochondria.ome.tiff (where *img_num* from 122 to 129)
- **z planes :**
    - number : 1
    - BF z focus : 11, 14, 15, 17 or 18 depending on the image
    - Nucleus z focus : 7 or 10 depending on the image
    - Mitochondria z focus : 5 or 8 depending on the image
- **channels** :
    - nucleus :
        - marker : Hoechst 33342
        - excitation light : 390/22 nm
        - emission light : 445/20 nm
    - mitochondria :
        - marker : Mito Red
        - excitation light : 575/33 nm
        - emission light : 641/75 nm

- **dimension order** : TCZYX
- **images size** : 1, 1, 1, 2044, 2048
- **physical size x** : 0.161700570874998 µm
- **physical size y** : 0.161700570874998 µm
- **physical size z** : 0.3 µm
- **type** : uint16
- **format** : ome.tiff

## Image Acquisition

- **imaging method** : BF, Fluorescence
- **imaging instrument** : Inverted Nikon Ti eclipse with NIS software and environmental control
- **pixel size** : 162 nm
- **light source** : Lumencor Spectra , LED
- **detector model** : Hamamatsu Orca Flash4 C11440-22C SN:000242
- **objective** :
    - **model** : Nikon Plan Fluor
    - **immersion** : Oil
    - **lens numerical aperture (NA)** : 1.3
    - **nominal magnification** : 40x
    - **working distance** : 0.24 mm
    - **refractive index** : 1.518

## Raw Image Notes

- **samples per pixel** : 1
- **z planes number** : 19
- **dimension order** : TCZYX
- **images size** : BF, Hoechst, Mito Red
- **channel order** : 1, 3, 19, 2044, 2048
- **type** : uint16
- **format** : .nd2
- **comments** (any help to reproduce the experiment / acquisition) **:** The labeling is reproducible in these settings and also stable for several hours.

# Study 9

## Authors


- **Stéphanie BOSCH**, CNRS, Univ Toulouse 3, TRI-LITC, CBI- FR 3743, F-31062 Toulouse cedex 9, France
  stephanie.bosch@univ-tlse3.fr
- **Maëlle CARRAZ**, IRD-CNRS, Univ Toulouse 3, PharmaDev UMR 152 and MCD UMR 5077, F-31062 Toulouse cedex 9, France
  maelle.carraz@ird.fr


- **Role :** MC prepared samples (cells and labeling), SB made the microscopy acquisitions.

- **France BioImaging node :** Toulouse
- **France BioImaging platform :** Centre de Biologie Intégrative (CBI)

## Description

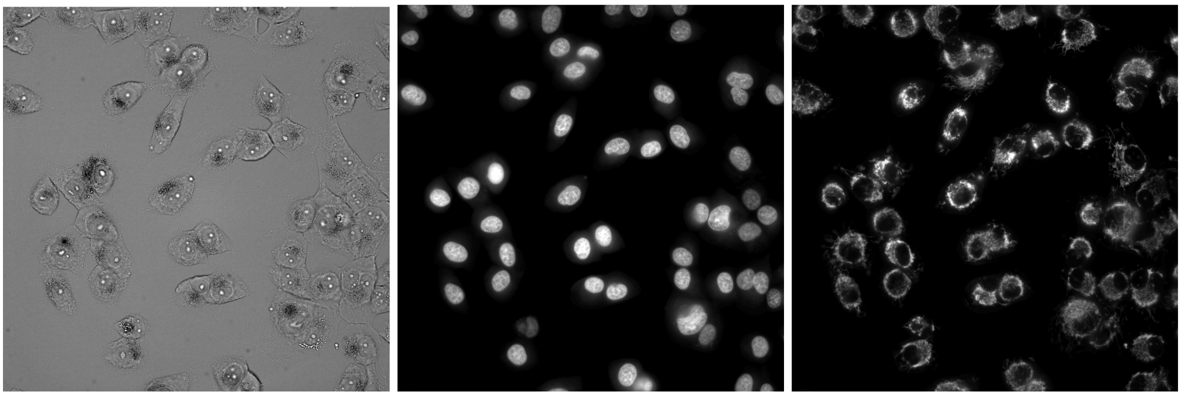

**a)** *image_130_BF_z5* **b)** *image_130_Nucleus* **c)** *image_130_Mitochondria*

**Fig.study 9:** *Example of images from Study 9.* ***a)*** *BF microscopy image of T24 human bladder cancer cells.* ***b)*** *BF image equivalent but in fluorescence microscopy of DRAQ5™- labeled human bladder cells nuclei.* ***c)*** *BF image equivalent but in fluorescence microscopy of MitoTracker™ green FM-labeled human bladder cells mitochondria.*

## Study Component

- **name** : Urinary bladder transitional cell carcinoma 40x
- **file list access** : json_file tsv_file

## Biosample

- **model** : human / homo sapiens
- **NCBI** : txid9606
- **biological entity** : T24 urinary bladder transitional cell carcinoma
- **ATCC catalog number** : HTB-4
- **description**: The T24 cell line was established from a transitional cell carcinoma of the bladder in a 82 years old female patient in 1973.

## Specimen

- **sample preparation** : Cells were plated in glass bottom micro-dish (35 mm) ibidi® in DMEM complete medium at 150,000 cells per plate, for 24h. Medium was then replaced by opti-MEM (without serum nor phenol red). Nuclei were labeled with DRAQ5 0.1% for 30 min. MitoTracker Green 0.1% was then added for 20 min at 37°C to stain mitochondria.
- **growth protocol** : T24 cells are adherent human cells cultivated in complete DMEM medium containing 10% FBS, 1% L-glutamine, sodium pyruvate and non-essential amino acids. They are trypsinized with trypsin-EDTA 0.05% every 3 days for passages.

## Image Data

- **number of images** : 168 with :

    - 152 BF : image_<*img_num*>_BF_z<*z_num*>.ome.tiff (where *num_img* from 130 to 137 and *z_num from 0 to 18*)
    - 8 Nucleus : image_<*img_num*>_Nucleus.ome.tiff (where *img_num* from 130 to 137)
    - 8 Mitochondria : image_<*img_num*>_Mitochondria.ome.tiff (where *img_num* from 130 to 137)
- **z planes :**
    - number : 1
    - BF z focus : 18
    - Nucleus z focus : 9 or 16 depending on the image
    - Mitochondria z focus : 4 or 11 depending on the image
- **channels** :
    - nucleus :
        - marker : DRAQ5
        - excitation light : 628/40 nm
        - emission light : 676/29 nm
    - mitochondria :
        - marker : MitoTracker Green
        - excitation light : 475/34 nm
        - emission light : 536/40 nm

- **dimension order** : TCZYX
- **images size** : 1, 1, 1, 2044, 2048
- **physical size x** :0.161700570874998 µm
- **physical size y** : 0.161700570874998 µm
- **physical size z** : 0.3 µm
- **type** : uint16
- **format** : ome.tiff

## Image Acquisition

- **imaging method** : BF, Fluorescence
- **imaging instrument** : Inverted Nikon Ti eclipse with NIS software and environmental control. z acquisition of 5µm with 0,3µm step size.
- **pixel size** : 162 nm
- **light source** : Lumencor Spectra , LED
- **detector model** : Hamamatsu OrcaFlash4 C11440-22C SN:000242
- **objective** :
    - **model** : Nikon Plan Fluor
    - **immersion** : Oil
    - **lens numerical aperture (NA)** : 1.3
    - **nominal magnification** : 40x
    - **working distance** : 0.24 mm
    - **refractive index** : 1.518

## Raw Image Notes

- **samples per pixel** : 1
- **z planes number** : 19
- **dimension order** : TCZYX
- **images size** : 1, 3, 19, 2044, 2048
- **channel order** : BF, MitoTracker Green, DRAQ5
- **type** : uint16
- **format** : ome.tif

# Study 10

## Authors


- **Stéphanie BOSCH**, CNRS, Univ Toulouse 3, TRI-LITC, CBI- FR 3743, F-31062 Toulouse cedex 9, France
  stephanie.bosch@univ-tlse3.fr
- **Maëlle CARRAZ**, IRD-CNRS, Univ Toulouse 3, PharmaDev UMR 152 and MCD UMR 5077, F-31062 Toulouse cedex 9, France
  maelle.carraz@ird.fr


- **Role :** MC prepared samples (cells and labeling), SB made the microscopy acquisitions.

- **France BioImaging node :** Toulouse
- **France BioImaging platform :** Centre de Biologie Intégrative (CBI)

## Description

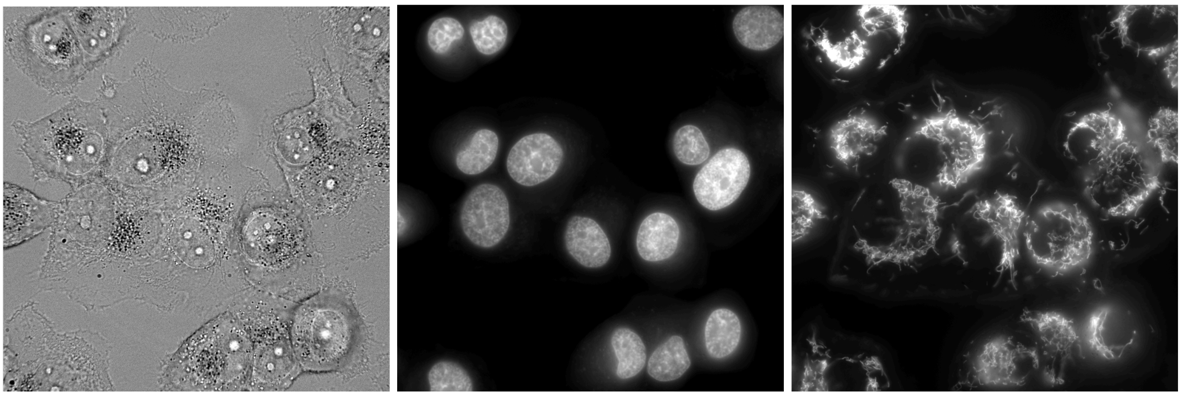

**a)** *image_130_BF_z5* **b)** *image_130_Nucleus* **c)** *image_130_Mitochondria*

**Fig.study 10:** *Example of images from Study 10.* ***a)*** *BF microscopy image of T24 human bladder cancer cells.* ***b)*** *BF image equivalent but in fluorescence microscopy of DRAQ5™-labeled human bladder cells nuclei.* ***c)*** *BF image equivalent but in fluorescence microscopy of MitoTracker™ Green FM- labeled human bladder cells mitochondria.*

## Study Component

- **name** : Urinary bladder transitional cell carcinoma (BF)
- **file list access** : json_file tsv_file

## Biosample

- **model** : human / homo sapiens
- **NCBI** : txid9606
- **biological entity** : T24 urinary bladder transitional cell carcinoma
- **ATCC catalog number** : HTB-4
- **description**: The T24 cell line was established from a transitional cell carcinoma of the bladder in a 82 years old female patient in 1973.

## Specimen

- **sample preparation** : Cells were plated in glass bottom micro-dish (35 mm) ibidi® in DMEM complete medium at 150,000 cells per plate, for 24h. Medium was then replaced by opti-MEM (without serum nor phenol red). Nuclei were labeled with DRAQ5 0.1% for 30 min. MitoTracker Green 0.1% was then added for 20 min at 37°C to stain mitochondria.
- **growth protocol** : T24 cells are adherent human cells cultivated in complete DMEM medium containing 10% FBS, 1% L-glutamine, sodium pyruvate and non-essential amino acids. They are trypsinized with trypsin-EDTA 0.05% every 3 days for passages.

## Image Data

- **number of images** : 232 with :

    - 216 BF : image_<*img_num*>_PC_z<*z_num*>.ome.tiff (where *num_img* from 138 to 145 and *z_num from 0 to 26*)
    - 8 Nucleus : image_<*img_num*>_Nucleus.ome.tiff (where *img_num* from 138 to 145)
    - 8 Mitochondria : image_<*img_num*>_Mitochondria.ome.tiff (where *img_num* from 138 to 145)
- **z planes :**
    - number : 1
    - BF z focus : 16, 19, 21, 23, 25 or 26 depending on the image
    - Nucleus z focus : 14, 24 or 26 depending on the image
    - Mitochondria z focus : 10, 11 or 13 depending on the image
- **channels** :
    - nucleus :
        - marker : DRAQ5
        - excitation light : 628/40 nm
        - emission light : 676/29 nm
    - mitochondria :
        - marker : MitoTracker Green
        - excitation light : 475/34 nm
        - emission light : 536/40 nm

- **dimension order** : TCZYX
- **images size** : 1, 1, 1, 2044, 2048
- **physical size x** : 0.065144126664423 µm
- **physical size y** : 0.065144126664423 µm
- **physical size z** : 0.2 µm
- **type** : uint16
- **format** : ome.tiff

## Image Acquisition

- **imaging method** : BF, Fluorescence
- **imaging instrument** : Inverted Nikon Ti eclipse with NIS software and environmental control. z acquisition of 5µm with 0,2µm step size.
- **pixel size** : 65 nm
- **light source** : Lumencor Spectra , LED
- **detector model** : Hamamatsu OrcaFlash4 C11440-22C SN:000242
- **objective** :
    - **model** : Nikon Plan Apo
    - **immersion** : Oil
    - **lens numerical aperture (NA)** : 1.4
    - **nominal magnification** : 100x
    - **working distance** : 0.130 mm
    - **refractive index** : 1.518

## Raw Image Notes

- **samples per pixel** : 1
- **z planes number** : 27
- **dimension order** : TCZYX
- **images size** : 1, 3, 27, 2044, 2048
- **channel order** : BF, MitoTracker Green, DRAQ5
- **type** : uint16
- **format** : .nd2
- **comments** (any help to reproduce the experiment / acquisition) **:** The staining is intense and stable for several hours.

# Study 11

## Authors


- **Stéphanie BOSCH**, CNRS, Univ Toulouse 3, TRI-LITC, CBI- FR 3743, F-31062 Toulouse cedex 9, France
stephanie.bosch@univ-tlse3.fr
- **Maëlle CARRAZ**, IRD-CNRS, Univ Toulouse 3, PharmaDev UMR 152 and MCD UMR 5077, F-31062 Toulouse cedex 9, France
maelle.carraz@ird.fr


- **Role :** MC prepared samples (cells and labeling), SB made the microscopy acquisitions.

- **France BioImaging node :** Toulouse
- **France BioImaging platform :** Centre de Biologie Intégrative (CBI)

## Description

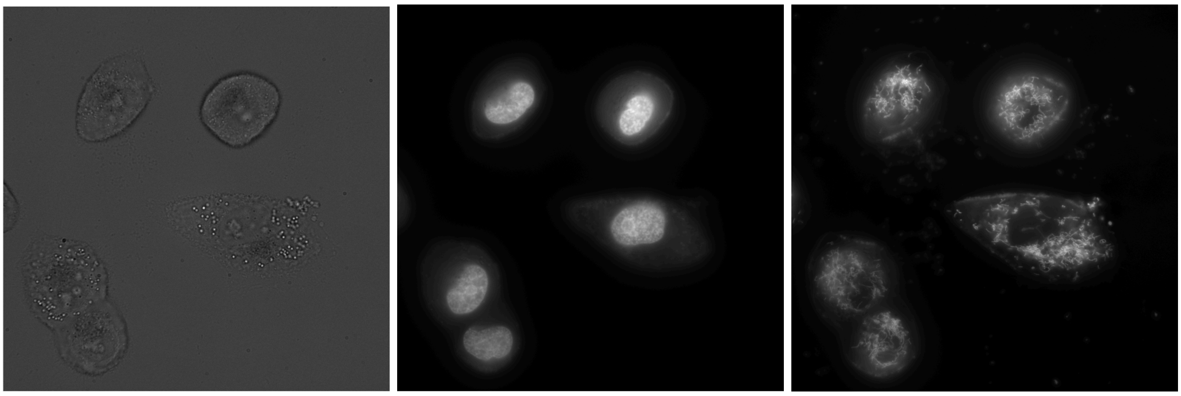

**a)** *image_147_BF_z5* **b)** *image_147_Nucleus* **c)** *image_147_Mitochondria*

**Fig.study 11:** *Example of images from Study 11.* ***a)*** *BF microscopy image of C4-2B human prostate cancer cells.* ***b)*** *BF image equivalent but in fluorescence microscopy of DRAQ5™- labeled human prostate cells nuclei.* ***c)*** *BF image equivalent but in fluorescence microscopy of MitoTracker Green-labeled human prostate cells mitochondria.*

## Study Component

- **name** : Human prostate cancer (BF)
- **file list access** : json_file tsv_file

## Biosample

- **model** : human / homo sapiens
- **NCBI** : txid9606
- **biological entity** : C4-2B human prostate cancer LNCaP-derived cells
- **ATCC catalog number** : CRL-3315
- **description**: C4-2B cells derived from the LNCaP cells in 1993. LNCaP cells were isolated from the prostate of a White male with prostate cancer.

## Specimen

- **sample preparation** : Cells were plated in glass bottom micro-dish (35 mm) ibidi® in DMEM/F12 complete medium at 300,000 cells per plate, for 24h. Medium was then replaced by opti-MEM (without serum nor phenol red). Nuclei were labeled with DRAQ5 0.1% for 30 min. MitoTracker Green 0.1% was then added for 20 min at 37°C to stain mitochondria.
- **growth protocol** : C4-2B cells are adherent human cells cultivated in complete DMEM/F12 medium containing 10% FBS, 1% L-glutamine, sodium pyruvate and non-essential amino acids. They are trypsinized with trypsin-EDTA 0.05% every 3 days for passages.

## Image Data

- **number of images** : 116 with :

    - 108 BF : image_<*img_num*>_BF_z<*z_num*>.ome.tiff (where *num_img* from 146 to 149 and *z_num from 0 to 30*)
    - 4 Nucleus : image_<*img_num*>_Nucleus.ome.tiff (where *img_num* from 146 to 149)
    - 4 Mitochondria : image_<*img_num*>_Mitochondria.ome.tiff (where *img_num* from 146 to 149)
- **z planes :**
    - number : 1
    - BF z focus : 18 or 26 depending on the image
    - Nucleus z focus : 26
    - Mitochondria z focus : 0 to 3
- **channels** :
    - nucleus :
        - marker : DRAQ5
        - excitation light : 628/40 nm
        - emission light : 676/29 nm
    - mitochondria :
        - marker : MitoTracker Green
        - excitation light : 475/34 nm
        - emission light : 536/40 nm

- **dimension order** : TCZYX
- **images size** : 1, 1, 1, 2044, 2048
- **physical size x** : 0.065144126664423 µm
- **physical size y** : 0.065144126664423 µm
- **physical size z** : 0.2 µm
- **type** : uint16
- **format** : ome.tiff

## Image Acquisition

- **imaging method** : BF, Fluorescence
- **imaging instrument** : Inverted Nikon Ti eclipse with NIS software and environmental control. z acquisition of 5µm with 0,2µm step size.
- **pixel size** : 65 nm
- **light source** : Lumencor Spectra , LED
- **detector model** : Hamamatsu OrcaFlash4 C11440-22C SN:000242
- **objective** :
    - **model** : Nikon Plan Apo
    - **immersion** : Oil
    - **lens numerical aperture (NA)** : 1.4
    - **nominal magnification** : 100x
    - **working distance** : 0.130 mm
    - **refractive index** : 1.518

## Raw Image Notes

- **samples per pixel** : 1
- **z planes number** : 27
- **dimension order** : TCZYX
- **images size** : 1, 3, 27, 2044, 2048
- **channel order** : BF, MitoTracker Green, DRAQ5
- **type** : uint16
- **format** : .nd2
- **comments** (any help to reproduce the experiment / acquisition) **:** The staining is intense and stable for several hours.

# Study 12

## Authors


- **Stéphanie BOSCH**, CNRS, Univ Toulouse 3, TRI-LITC, CBI- FR 3743, F-31062 Toulouse cedex 9, France
  stephanie.bosch@univ-tlse3.fr
- **Maëlle CARRAZ**, IRD-CNRS, Univ Toulouse 3, PharmaDev UMR 152 and MCD UMR 5077, F-31062 Toulouse cedex 9, France
  maelle.carraz@ird.fr


- **Role :** MC prepared samples (cells and labeling), SB made the microscopy acquisitions.

- **France BioImaging node :** Toulouse
- **France BioImaging platform :** Centre de Biologie Intégrative (CBI)

## Description

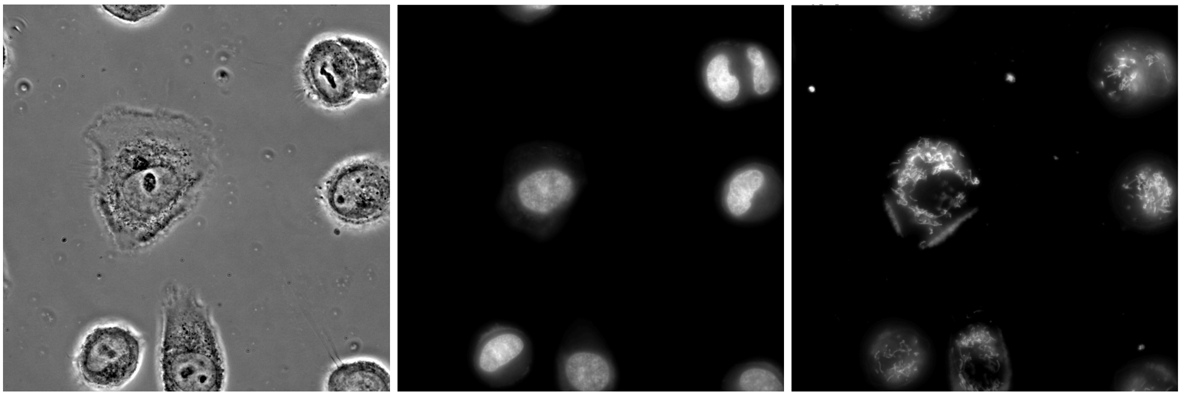

**a)** *image_155_PC_z23* **b)** *image_155_Nucleus* **c)** *image_155_Mitochondria*

**Fig.study 12:** *Example of images from Study 12.* ***a)*** *PC microscopy image of C4-2B human cancer prostate cells.* ***b)*** *PC image equivalent but in fluorescence microscopy of DRAQ5™-labeled C4-2B human prostate cells nuclei.* ***c)*** *PC image equivalent but in fluorescence microscopy of MitoTracker™ Green-labeled C4-2B human prostate cells mitochondria.*

## Study Component

- **name** : Human Prostate Cancer (PC)
- **file list access** : [json_file](#) [tsv_file](#)

## Biosample

- **model** : human / homo sapiens
- **NCBI** : txid9606
- **biological entity** : C4-2B human prostate cancer
- **ATCC catalog number** : CRL-3315
- **description**: C4-2B is a cell line with epithelial-like morphology that was derived from human prostate cancer LNCaP-derived C4-2 cells in 1993. LNCaP cells were isolated from the prostate of a White male with prostate cancer.

## Specimen

- **sample preparation** : Cells were plated in glass bottom micro-dish (35 mm) ibidi® in DMEM/F12 complete medium at 300,000 cells per plate, for 24h. Medium was then replaced by opti-MEM (without serum nor phenol red). Nuclei were labeled with DRAQ5 0.1% for 30 min. MitoTracker Green 0.1% was then added for 20 min at 37°C to stain mitochondria.
- **growth protocol** : C4-2B cells are adherent human cells cultivated in complete DMEM/F12 medium containing 10% FBS, 1% L-glutamine, sodium pyruvate and non-essential amino acids. They are trypsinized with trypsin-EDTA 0.05% every 3 days for passages.

## Image Data

- **number of images** : 232 with :

    - 216 PC : image_<*img_num*>_BF_z<*z_num*>.ome.tiff (where *num_img* from 150 to 157 and *z_num from 0 to 26*)
    - 8 Nucleus : image_<*img_num*>_Nucleus.ome.tiff (where *img_num* from 150 to 157)
    - 8 Mitochondria : image_<*img_num*>_Mitochondria.ome.tiff (where *img_num* from 150 to 157)
- **z planes :**
    - number : 1
    - PC z focus : 7, 11, 12, 19 or 26 depending on the image
    - Nucleus z focus : 26
    - Mitochondria z focus : 1 or 4
- **channels** :
        - marker : DRAQ5
        - excitation light : 628/40 nm
        - emission light : 676/29 nm
    - mitochondria :
        - marker : MitoTracker Green
        - excitation light : 475/34 nm
        - emission light : 536/40 nm

- **dimension order** : TCZYX
- **images size** : 1, 1, 1, 2044, 2048
- **physical size x** : 0.06458829759855 µm
- **physical size y** : 0.06458829759855 µm
- **physical size z** : 0.2 µm
- **type** : uint16
- **format** : ome.tiff

## Image Acquisition

- **imaging method** : PC, Fluorescence
- **imaging instrument** : Inverted Nikon Ti eclipse with NIS software and environmental control. z acquisition of 5µm with 0,2µm step size.
- **pixel size** : 65 nm
- **light source** : Lumencor Spectra , LED
- **detector model** : Hamamatsu OrcaFlash 4 C11440-22C SN:000242
- **objective** :
    - **model** : Nikon Plan Fluor Ph3 DLL
    - **immersion** : Oil
    - **lens numerical aperture (NA)** : 1.3
    - **nominal magnification** : 100x
    - **working distance** : 0.2 mm
    - **refractive index** : 1.518

## Raw Image Notes

- **samples per pixel** : 1
- **z planes number** : 27
- **dimension order** : TCZYX
- **images size** : 1, 3, 31, 2044, 2048
- **channel order** : PC, Mito Tracker Green, DRAQ5
- **type** : uint16
- **format** : .nd2
- **comments** (any help to reproduce the experiment / acquisition) **:**The staining is intense and stable for several hours.

# Study 13

## Authors


- **Stéphanie BOSCH**, CNRS, Univ Toulouse 3, TRI-LITC, CBI- FR 3743, F-31062 Toulouse cedex 9, France
  stephanie.bosch@univ-tlse3.fr
- **Maëlle CARRAZ**, IRD-CNRS, Univ Toulouse 3, PharmaDev UMR 152 and MCD UMR 5077, F-31062 Toulouse cedex 9, France
  maelle.carraz@ird.fr


- **Role :** MC prepared samples (cells and labeling), SB made the microscopy acquisitions.

- **France BioImaging node :** Toulouse
- **France BioImaging platform :** Centre de Biologie Intégrative (CBI)

## Description

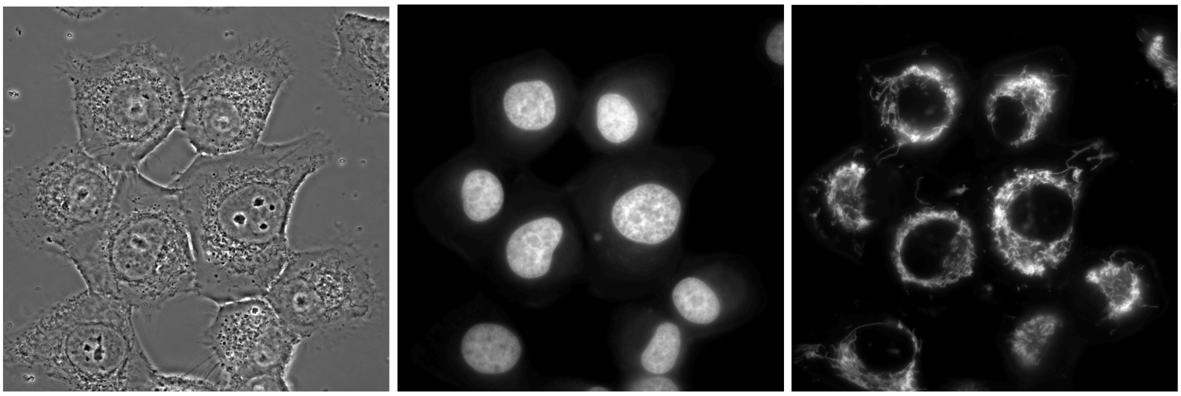

**a)** *image_160_PC_z16* **b)** *image_160_Nucleus* **c)** *image_160_Mitochondria*

**Fig.study 13:** *Example of images from Study 13.* ***a)*** *PC microscopy image of T24 human bladder cells.* ***b)*** *PC image equivalent but in fluorescence microscopy of DRAQ5™- labeled human bladder cells nuclei.* ***c)*** *PC image equivalent but in fluorescence microscopy of MitoTracker™ Green- labeled human bladder cells mitochondria.*

## Study Component

- **name** : Urinary bladder transitional cell carcinoma (PC)
- **file list access** : json_file tsv_file

## Biosample

- **model** : human / homo sapiens
- **NCBI** : txid9606
- **biological entity** : T24 urinary bladder transitional cell carcinoma
- **ATCC catalog number** : HTB-4
- **description**: The T24 cell line was established from a transitional cell carcinoma of the bladder in a 82 years old female patient in 1973.

## Specimen

- **sample preparation** : Cells were plated in glass bottom micro-dish (35 mm) ibidi® in DMEM complete medium at 150,000 cells per plate, for 24h. Medium was then replaced by opti-MEM (without serum nor phenol red). Nuclei were labeled with DRAQ5 0.1% for 30 min. MitoTracker Green 0.1% was then added for 20 min at 37°C to stain mitochondria.
- **growth protocol** : T24 cells are adherent human cells cultivated in complete DMEM medium containing 10% FBS, 1% L-glutamine, sodium pyruvate and non-essential amino acids. They are trypsinized with trypsin-EDTA 0.05% every 3 days for passages.

## Image Data

- **number of images** : 232 with :
  - 216 PC : image_<*img_num*>_PC_z<*z_num*>.ome.tiff (where *num_img* from 158 to 165 and *z_num from 0 to 26*)

    - 8 Nucleus : image_<*img_num*>_Nucleus.ome.tiff (where *img_num* from 158 to 165)
    - 8 Mitochondria : image_<*img_num*>_Mitochondria.ome.tiff (where *img_num* from 158 to 165)
- **z planes :**
    - number : 1
    - PC z focus : 7, 15, 17, 18, 19 or 20 depending on the image
    - Nucleus z focus : 21, 22 or 25 depending on the image
    - Mitochondria z focus : 9, 10, 11 depending on the image
- **channels** :
    - nucleus :
        - marker : Draq5
        - excitation light : 628/40 nm
        - emission light : 676/29 nm
    - mitochondria :
        - marker : MitoTracker Green
        - excitation light : 475/34 nm
        - emission light : 536/40 nm

- **dimension order** : TCZYX
- **images size** : 1, 1, 1, 2044, 2048
- **physical size x** : 0.064381178211829 µm
- **physical size y** : 0.064381178211829 µm
- **physical size z** : 0.2 µm
- **type** : uint16
- **format** : ome.tiff

## Image Acquisition

- **imaging method** : PC, Fluorescence
- **imaging instrument** : Inverted Nikon Ti eclipse with NIS software and environmental control. z acquisition of 5µm with 0,2µm step size.
- **pixel size** : 65 nm
- **light source** : Lumencor Spectra , LED
- **detector model** : Hamamatsu orcaFlash 4.0 C11440-22C SN:000242
- **objective** :
    - **model** : Nikon Plan Apo Ph3 DM
    - **immersion** : Oil
    - **lens numerical aperture (NA)** : 1.4
    - **nominal magnification** : 100x
    - **working distance** : 0.130 mm
    - **refractive index** : 1.518

## Raw Image Notes

- **samples per pixel** : 1
- **z planes number** : 27
- **dimension order** : TCZYX
- **images size** : 1, 3, 27, 2044, 2048
- **channel order** : PC, Mito Tracker Green, DRAQ5
- **type** : uint16
- **format** : .nd2
- **comments** (any help to reproduce the experiment / acquisition) **:**The staining is intense and stable for several hours.

# Study 14

## Authors


- **Stéphanie BOSCH**, CNRS, Univ Toulouse 3, TRI-LITC, CBI- FR 3743, F-31062 Toulouse cedex 9, France
  stephanie.bosch@univ-tlse3.fr
- **Maëlle CARRAZ**, IRD-CNRS, Univ Toulouse 3, PharmaDev UMR 152 and MCD UMR 5077, F-31062 Toulouse cedex 9, France
  maelle.carraz@ird.fr


- **Role :** MC prepared samples (cells and labeling), SB made the microscopy acquisitions.

- **France BioImaging node :** Toulouse
- **France BioImaging platform :** Centre de Biologie Intégrative (CBI)

## Description

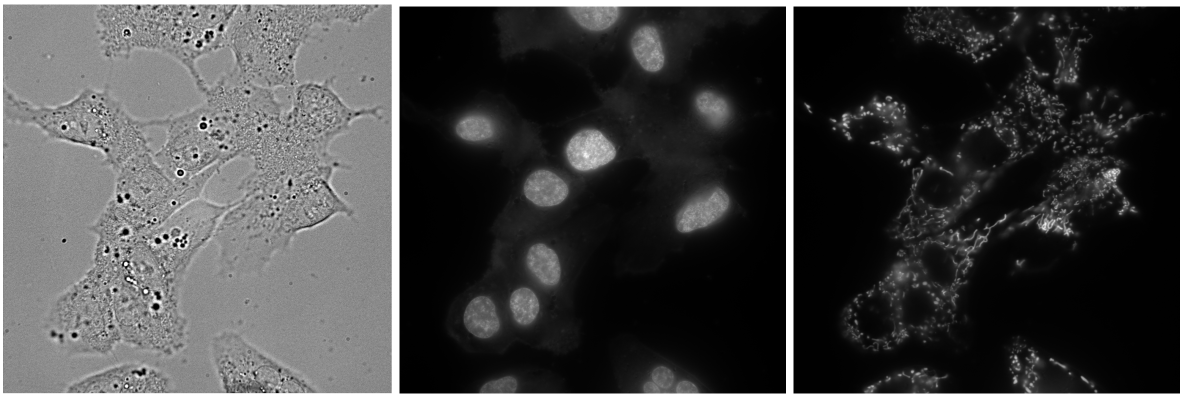

**a)** *image_171_BF_z18* **b)** *image_171_Nucleus* **c)** *image_171_Mitochondria*

**Fig.study 14:** *Example of images from Study 14.* ***a)*** *BF microscopy image of Hep3B human liver cancer cells.* ***b)*** *BF image equivalent but in fluorescence microscopy of Hoechst- labeled Hep3B human liver cells nuclei.* ***c)*** *BF image equivalent but in fluorescence microscopy of Mito Red- labeled Hep3B human liver cells mitochondria.*

## Study Component

- **name** : Human Liver Carcinoma (BF)
- **file list access** : json_file tsv_file

## Biosample

- **model** : human / homo sapiens
- **NCBI** : txid9606
- **biological entity** : Hep3B human liver carcinoma
- **ATCC catalog number** : Hep 3B2.1-7, HB-8064
- **description**: Hep3B is a cell line exhibiting epithelial morphology that was isolated from liver tissue derived from an 8-year-old, Black youth with liver cancer. This cell line contains an integrated hepatitis B virus genome.

## Specimen

- **sample preparation** : Cells were plated in glass bottom micro-dish (35 mm) ibidi® in DMEM complete medium at 250,000 cells per plate, for 24h. Medium was then replaced by opti-MEM (without serum nor phenol red). Nuclei were labeled with 0.5µL/mL Hoechst 33342, 10 min. Mito Red was then added at a final concentration of 10 nM during 30 min at 37°C to stain mitochondria.
- **growth protocol** : Hep3B cells are adherent human cells cultivated in complete DMEM medium containing 10% FBS, 1% L-glutamine, sodium pyruvate and non-essential amino acids. They are trypsinized with trypsin-EDTA 0.05% every 3 days for passages.

## Image Data

- **number of images** : 184 with :
  - 168 PC : image_<*img_num*>_PC_z<*z_num*>.ome.tiff (where *num_img* from 166 to 173 and *z_num from 0 to 20*)
  - 8 Nucleus : image_<*img_num*>_Nucleus.ome.tiff (where *img_num* from 166 to 173)
  - 8 Mitochondria : image_<*img_num*>_Mitochondria.ome.tiff (where *img_num* from 166 to 173)
- **z planes :**
  - number : 1
  - BF z focus : 1, 4, 5, 14 or 15 depending on the image
  - Nucleus z focus : 4 or 11 depending on the image
  - Mitochondria z focus : 0 or 7 depending on the image
- **channels** :
  - nucleus :
    - marker : Hoechst 33342
    - excitation light : 390/22 nm
    - emission light : 445/20 nm
  - mitochondria :
    - marker : Mito Red
    - excitation light : 575/33 nm
    - emission light : 641/75 nm

- **dimension order** : TCZYX
- **images size** : 1, 1, 1, 2044, 2048
- **physical size x** : 0.065144126664423 µm
- **physical size y** : 0.065144126664423 µm
- **physical size z** : 0.2 µm
- **type** : uint16
- **format** : ome.tiff

## Image Acquisition

- **imaging method** : BF, Fluorescence
- Inverted Nikon Ti eclipse with NIS software and environmental control. z acquisition of 5µm with 0,2µm step size.
- **pixel size** : 65 nm
- **light source** : Lumencor Spectra , LED
- **detector model** : Hamamatsu orcaFlash 4.0 C11440-22C SN:000242
- **objective** :
  - **model** : Nikon Plan Apo
  - **immersion** : Oil
  - **lens numerical aperture (NA)** : 1.4
  - **nominal magnification** : 100x
  - **working distance** : 0.130 mm
  - **refractive index** : 1.518

## Raw Image Notes

- **samples per pixel** : 1
- **z planes number** : 21
- **dimension order** : TCZYX
- **images size** : 1, 3, 21 2044, 2048
- **channel order** : BF, Mito Red, Hoechst
- **type** : uint16
- **format** : .nd2
- **comments** (any help to reproduce the experiment / acquisition) **:**The staining is intense and stable for several hours.

## Study 15

### Authors


- **Stéphanie BOSCH**, CNRS, Univ Toulouse 3, TRI-LITC, CBI- FR 3743, F-31062 Toulouse cedex 9, France
  stephanie.bosch@univ-tlse3.fr
- **Maëlle CARRAZ**, IRD-CNRS, Univ Toulouse 3, PharmaDev UMR 152 and MCD UMR 5077, F-31062 Toulouse cedex 9, France
  maelle.carraz@ird.fr


- **Role :** MC prepared samples (cells and labeling), SB made the microscopy acquisitions.

- **France BioImaging node :** Toulouse
- **France BioImaging platform :** Centre de Biologie Intégrative (CBI)

### Description

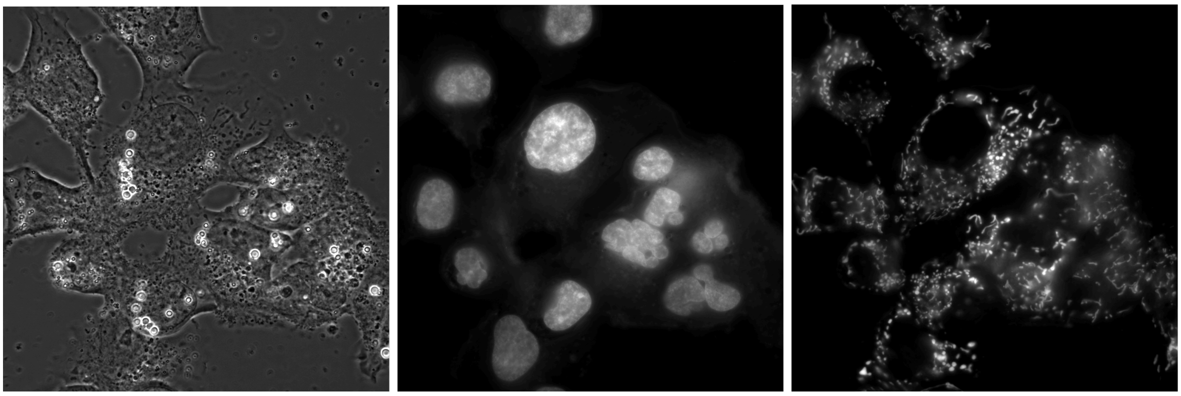

**a)** *image_174_PC_z17* **b)** *image_174_Nucleus* **c)** *image_174_Mitochondria*

**Fig.study 15:** *Example of images from Study 15.* ***a)*** *PC microscopy image of Hep3B human liver cancer cells.* ***b)*** *PC image equivalent but in fluorescence microscopy of Hoechst- labeled Hep3B human liver cells nuclei.* ***c)*** *PC image equivalent but in fluorescence microscopy of Mito Red-labeled Hep3B human liver cells mitochondria.*

### Study Component

- **name** : Human Liver Carcinoma (PC)
- **file list access** : [json_file](json_file) [tsv_file](tsv_file)

### Biosample

- **model** : human / homo sapiens
- **NCBI** : txid9606
- **biological entity** : Hep3B human liver carcinoma
- **ATCC catalog number** : Hep 3B2.1-7, HB-8064
- **description**: Hep3B is a cell line exhibiting epithelial morphology that was isolated from liver tissue derived from an 8-year-old, Black youth with liver cancer. This cell line contains an integrated hepatitis B virus genome.

### Specimen

- **sample preparation** : Cells were plated in glass bottom micro-dish (35 mm) ibidi® in DMEM complete medium at 250,000 cells per plate, for 24h. Medium was then replaced by opti-MEM (without serum nor phenol red). Nuclei were labeled with 0.5µL/mL Hoechst 33342, 10 min. Mito Red was then added at a final concentration of 10 nM during 30 min at 37°C to stain mitochondria.
- **growth protocol** : Hep3B cells are adherent human cells cultivated in complete DMEM medium containing 10% FBS, 1% L-glutamine, sodium pyruvate and non-essential amino acids. They are trypsinized with trypsin-EDTA 0.05% every 3 days for passages.

### Image Data

- **number of images** : 116 with :

    - 108 PC : image_<*img_num*>_PC_z<*z_num*>.ome.tiff (where *num_img* from 174 to 177 and z*_num from 0 to 26*)
    - 4 Nucleus : image_<*img_num*>_Nucleus.ome.tiff (where *img_num* from 174 to 177 )
    - 4 Mitochondria : image_<*img_num*>_Mitochondria.ome.tiff (where *img_num* from 174 to 177 )
- **z planes :**
    - number : 1
    - PC z focus : 15, 16, 17 or 19 depending on the image
    - Nucleus z focus : 15
    - Mitochondria z focus : 11
- **channels** :
    - nucleus :
        - marker : Hoechst 33342
        - excitation light : 390/22 nm
        - emission light : 445/20 nm
    - mitochondria :
        - marker : Mito Red
        - excitation light : 575/33 nm
        - emission light : 641/75 nm

- **dimension order** : TCZYX
- **images size** : 1, 1, 1, 2044, 2048
- **physical size x** : 0.064381178211829 µm
- **physical size y** : 0.064381178211829 µm
- **physical size z** : 0.2 µm
- **type** : uint16
- **format** : ome.tiff

## Image Acquisition

- **imaging method** : PC, Fluorescence
- **imaging instrument** : Inverted Nikon Ti eclipse with NIS software and environmental control. z acquisition of 5µm with 0,2µm step size.
- **pixel size** : 65 nm
- **light source** : Lumencor Spectra , LED
- **detector model** : Hamamatsu orcaFlash 4.0 C11440-22C SN:000242
- **objective** :
    - **model** : Nikon Plan Apo Ph3 DM
    - **immersion** : Oil
    - **lens numerical aperture (NA)** : 1.4
    - **nominal magnification** : 100x
    - **working distance** : 0.130 mm
    - **refractive index** : 1.518

## Raw Image Notes

- **samples per pixel** : 1
- **z planes number** : 27
- **dimension order** : TCZYX
- **images size** : 1, 3, 27, 2044, 2048
- **channel order** : PC, Mito Red, Hoechst
- **type** : uint16
- **format** : .nd2
- **comments** (any help to reproduce the experiment / acquisition) **:**The staining is intense and stable for several hours.

# Study 16

## Authors


- **Yves LUTZ**, Inserm, Light Microscopy Facility IGBMC UMR 7104 - Inserm U1258, France
  lutz@igbmc.fr
- **Bertrand VERNAY**, Inserm, Light Microscopy Facility IGBMC UMR 7104 - Inserm U1258, France
  vernayb@igbmc.fr
- **Erwan GANDON**, Univ Strasbourg, Light Microscopy Facility IGBMC UMR 7104 - Inserm U1258, France
  erwan.gandon@nikon.com


- **Role :** YL prepared the sample, EG made the acquisition , BV supervised the project

- **France BioImaging node :** Alsace
- **France BioImaging platform :** Institut de génétique et de biologie moléculaire et cellulaire (IGBMC)

## Description

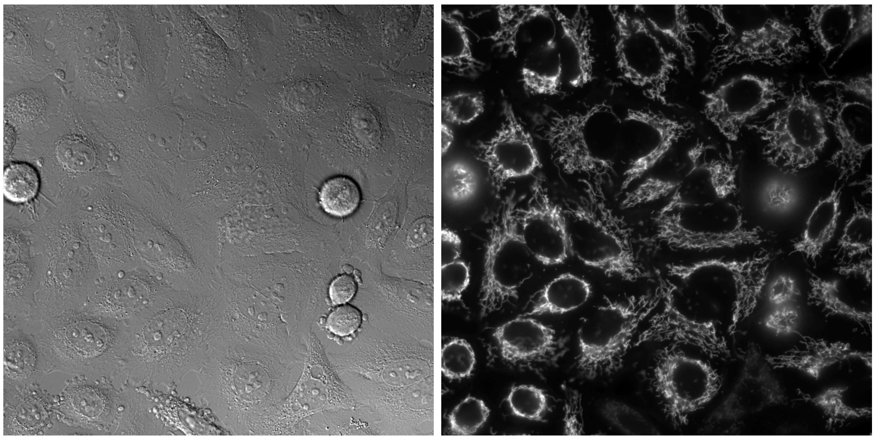

**a)** *image_178_DIC_z8* **b)** *image_178_Mitochondria*

**Fig.study 16:** *Example of images from Study 16.* ***a)*** *DIC microscopy image of* HeLa *human cells.* ***b)*** *DIC image equivalent but in fluorescence microscopy of MitoTracker-labeled* HeLa *human mitochondria.*

## Study Component

- **name** : HeLa Schaffner
- **file list access** : json_file tsv_file

## Biosample

- **model** : human / homo sapiens
- **NCBI** : txid9606
- **biological entity** : HeLa Cells
- **ATCC catalog number** : CCL-2
- **description**: HeLa Walter Schaffner

## Specimen

- **sample preparation** : Glass bottom (#1.5) petri. For DIC and fluorescence acquisition, plastic lead was replaced by a 40mm diameter glass coverslip.
- **growth protocol** : HeLa WS cells are adherent human cells cultured in DMEM 1g glucose/ml,10% FCS,40 µg/ml Gentamycin and trypsinized with trypsin 0.0375% for passages.

## Image Data

- **number of images** : 693 with :

    - 660 DIC : image_<*img_num*>_DIC_z<*z_num*>.ome.tiff (where *num_img* from 178 to 210 and *z_num from 0 to 19*)
    - 33 Mitochondria : image_<*img_num*>_Mitochondria.ome.tiff (where *img_num* from 178 to 210 )
- **z planes :**
    - number : 1
    - DIC z focus : 2, 4, 5, 6, 7, 8, 9, 10, 11 or 12 depending on the image
    - Mitochondria z focus : 4, 5, 6, 7, 8, 9, 10 or 11 depending on the image
- **channels** :
    - mitochondria :
        - marker : MitoTracker Red CMXRos M7512
        - excitation maximum : 578 nm
        - emission maximum : 641/75 nm
- **dimension order** : TCZYX
- **images size** : 1, 1, 1, 2044, 2048
- **physical size x** : 0.10317460317460317 µm
- **physical size y** : 0.10317460317460317 µm
- **physical size z** : 0.31 µm
- **type** : uint16
- **format** : ome.tiff

## Image Acquisition

- **imaging method** : DIC, Fluorescence
- **imaging instrument** : Inverted Zeiss Axio Observer stand, equipped with environmental control (37°C and CO2) controlled by ZEN software.
- **pixel size** : 103 nm
- **light source** : Colibri 7 (wavelength : Lime LED 567/100 nm + filter 590 nm (591/27) at 5% and Exposure Time 50 ms Emission filter: 618-756 nm), TL LED at 30% and Exposure Time 50 ms
- **detector model** : Hamamatsu ORCA-Flash4.0LT Digital Camera C11440-42U30 S. No. 101735
- **objective** :
    - **model** : ZeissPlan-Apochromat M27 1.4/63x DIC II
    - **immersion** : Oil
    - **lens numerical aperture (NA)** : 1.4
    - **nominal magnification** : 63x
    - **working distance** : 0.19 mm
    - **refractive index** : 1.518

## Raw Image Notes

- **samples per pixel** : 1
- **z planes number** : 20
- **dimension order** : TCZYX
- **images size** : 1, 2, 20, 2048, 2048
- **channel order** : DIC, MitoTracker Red
- **type** : uint16
- **format** : .czi

# Study 17

## Authors


- **Yves LUTZ**, Inserm, Light Microscopy Facility IGBMC UMR 7104 - Inserm U1258, France
  lutz@igbmc.fr
- **Bertrand VERNAY**, Inserm, Light Microscopy Facility IGBMC UMR 7104 - Inserm U1258, France
  vernayb@igbmc.fr
- **Erwan GANDON**, Univ Strasbourg, Light Microscopy Facility IGBMC UMR 7104 - Inserm U1258, France
  gandone@igbmc.fr


- **Role :** YL prepared the sample, EG made the acquisition , BV supervised the project

- **France BioImaging node :** Alsace
- **France BioImaging platform :** Institut de génétique et de biologie moléculaire et cellulaire (IGBMC)

## Description

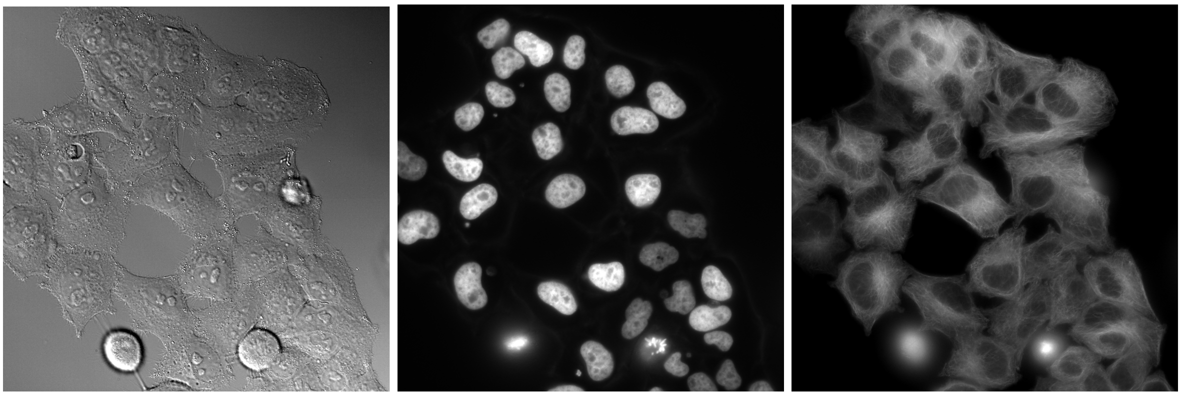

**a)** *image_225_DIC_z0* **b)** *image_225_Nucleus* **c)** *image_225_Tubulin*

**Fig.study 17:** *Example of images from Study 17.* ***a)*** *DIC microscopy image of HeLa human cells.* ***b)*** *DIC image equivalent but in fluorescence microscopy of H2B-mCherry-labeled HeLa human nuclei.* ***c)*** *DIC image equivalent but in fluorescence microscopy of GFP-Tubulin Hela human cells.*

## Study Component

- **name** : HeLa Vienna
- **file list access** : json_file tsv_file

## Biosample

- **model** : human / homo sapiens
- **NCBI** : txid9606
- **biological entity** : HeLa Cells mEGFP-alpha tubulin/ H2B-mCherry -
- **ATCC catalog number** : Not referenced
- **description**: from Dr. Daniel Gerlich IMBA Vienna HeLa Cells stably expressing mEGFP-alpha Tubulin/H2B-mCherry

## Specimen

- **sample preparation** : Cells were plated in petri glass bottom dish (#1,5/35 mm: CellVis D35-20-1.5H) in DMEM (4.5g glucose/ml,10% FCS,1% Penicillin/Streptomycin), for 2 days to reach 50% of confluency. Medium was then replaced by DMEM without phenol red containing MitoTracker Red CMXRos at a final concentration of 10 nM during 4.5 hours. For DIC and fluorescence acquisition, plastic lead was replaced by a 40mm diameter glass coverslip.
- **growth protocol** : HeLa mEGFP-αTubulin+H2B-mCherry cells are adherent human cells cultured in DMEM (4.5g glucose/ml,10% FCS,1% Penicillin/Streptomycin) and trypsinized with trypsin 0.0375% for passages.

## Image Data

- **number of images** : 902 with :
    - 820 DIC : image_<*img_num*>_DIC_z<*z_num*>.ome.tiff (where *num_img* from 211 to 251 and *z_num from 0 to 19*)
    - 41 Nucleus : image_<*img_num*>_Nucleus.ome.tiff (where *img_num* from 211 to 251 )
    - 41 Tubulin : image_<*img_num*>_Tubulin.ome.tiff (where *img_num* from 211 to 251)
- **z planes :**
    - number : 1
    - DIC z focus : from 0 to 12, 14, 17 or 19 depending on the image
    - Nucleus z focus : 4, 6, 7, 8, 10, 12, 13 or 18 depending on the image
    - Tubulin z focus : from 1 to 8, 10, 11, 12, 13, 14, 17 or 19 depending on the image
- **channels** :
    - nucleus :
        - marker : mCherry-H2B
        - excitation light : 587 nm
        - emission light : 610 nm
    - tubulin :
        - marker : GFP Tubulin
        - excitation light : 488 nm
        - emission light : 516 nm

- **dimension order** : TCZYX
- **images size** : 1, 1, 1, 2044, 2048
- **physical size x** : 0.10317460317460317 µm
- **physical size y** : 0.10317460317460317 µm
- **physical size z** : 0.26 µm
- **type** : uint16
- **format** : ome.tiff

## Image Acquisition

- **imaging method** : DIC, Fluorescence
- **imaging instrument** : Inverted Zeiss Axio Observer stand, equipped with environmental control (37°C and CO2) controlled by ZEN software.
- **pixel size** : 103 nm
- **light source** : Colibri 7 (wavelength : LED 567nm + filter 590 nm (591/27) at 5% and Exposure 100 ms Emission filter: 618-756 nm), Colibri 7 (wavelength : LED 475 nm (469/38) at 5% and Exposure 100 ms Emission filter: 500-530 nm), TL LED at 30% and Exposure Time 50 ms
- **detector model** : Hamamatsu ORCA-Flash4.0LT Digital Camera C11440-42U30 S. No. 101735
- **objective** :
    - **model** : Zeiss Plan-Apochromat M27 1.4/63x DIC II
    - **immersion** : Oil
    - **lens numerical aperture (NA)** : 1.4
    - **nominal magnification** : 63x
    - **working distance** : 0.19 mm
    - **refractive_index** : 1.518 (23°C)

## Raw Image Notes

- **samples per pixel** : 1
- **z planes number** : 20
- **dimension order** : TCZYX
- **images size** : 1, 3, 20, 2048, 2048
- **channel order** : GFP Tubulin, DIC, mCherry-H2B
- **type** : uint16
- **format** : .czi

# Study 18

## Authors

- **Virginie GEORGET**, 1) MRI, BioCampus, University of Montpellier (UM), CNRS, INSERM, Montpellier, France ; 2) CRBM, UM, CNRS, Montpellier, France. virginie.georget@mri.cnrs.fr
- **Juliette VAN DIJK**, CRBM, UM, CNRS, Montpellier France juliette.van-dijk@crbm.cnrs.fr

- **Role :** JVD prepared the sample, VG made the acquisition.

- **France BioImaging node :** Montpellier
- **France BioImaging platform :** Montpellier Ressources Imagerie (MRI), Centre de Recherche en Biologie cellulaire de Montpellier (CRBM)

## Description

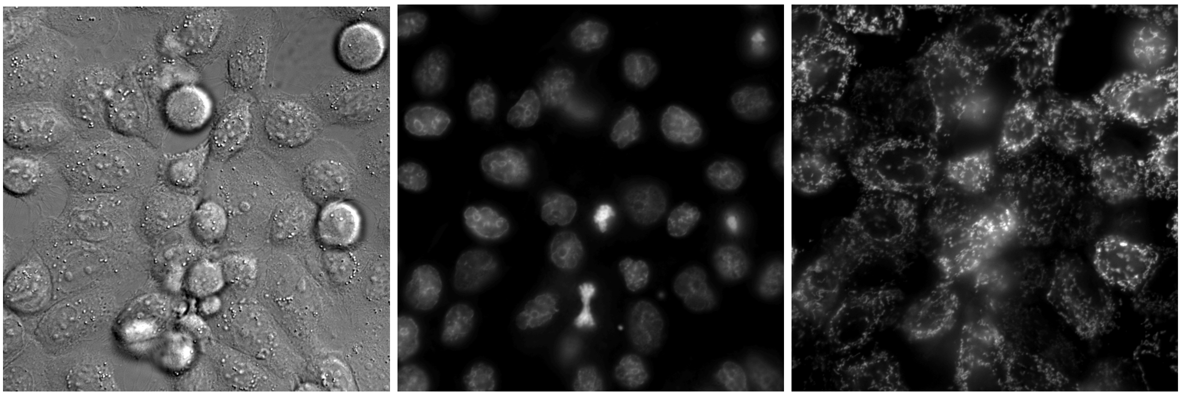

**a)** *image_270_DIC_z4* **b)** *image_270_Nucleus* **c)** *image_270_Mitochondria*

**Fig.study 18:** *Example of images from Study 18.* ***a)*** *DIC microscopy image of MDCK dog cells.* ***b)*** *DIC image equivalent but in fluorescence microscopy of Hoechst-labeled MDCK dog nuclei.* ***c)*** *DIC image equivalent but in fluorescence microscopy of MitoTracker-far-red-labeled MDCK dog mitochondria.*

## Study Component

- **name** : Cocker
- **file list access** : json_file tsv_file

## Biosample

- **model** : Canis lupus familiaris / dog
- **NCBI** : txid9615
- **biological entity** : MDCK expressing stably the MISP protein fused to Cherry.
- **ATCC catalog number** : Not referenced
- **description**: Cells isolated from normal kidney tissue. Normal, adult, female cocker spaniel.

## Specimen

- **sample preparation** : Cells were plated in glass bottom (#1,5) 24-well plate. Cells were pre-incubated in HBSS 1X buffer, then HBSS with far-red mitotracker (1/4000) for 20 minutes at 37°C for mitochondria staining. Medium was replaced by fluorobrite and cells were imaged a few hours later adding vital Hoechst (1/4000) a few minutes before.
- **growth protocol** : DMEM Glutamax 10% SVF (Sérum de Veau foetal) (FVS) + 1% PS (penicillin spectromycin).

## Image Data

- **number of images** : 253 with :
  - 207 DIC : image_<*n_img*>_DIC_z<*n_z*>.ome.tiff (where *n_img* from 252 to 274 and *n_z from 0 to 8*)
  - 23 Nucleus : image_<*n_img*>_Nucleus.ome.tiff (where *n_img* from 252 to 274)

    - 23 Mitochondria : image_<*n_img*>_*Mitochondria* .ome.tiff (where *n_img* from 252 to 274)
- **z planes :**
    - number : 1
    - DIC z focus : 4 or 5 depending on the image
    - Nucleus z focus : 5
    - Mitochondria z focus : 4
- **channels** :
    - nucleus :
        - marker : Hoechst
        - excitation light : 395/25 nm
        - emission light : 433/65 nm
    - mitochondria :
        - marker : Mitotracker Far Red
        - excitation light : 640/30 nm
        - emission light : 705/65 nm

- **dimension order** : TCZYX
- **images size** : 1, 1, 1, 1200, 1200
- **physical size x** : 0.109929394588707 µm
- **physical size y** : 0.109929394588707 µm
- **physical size z** : 3.0 µm
- **type** : uint16
- **format** : ome.tiff

## Image Acquisition

- **imaging method** : DIC, Fluorescence
- **imaging instrument** : Inverted Nikon Ti2 microscope with temperature and $CO_2$ controlled chamber, 100X/1.4 NA oil objective, Luminencor Led, CMOS back-illuminated Prime95B Photometrics camera. Motorized Z of the microscope for the Z-stack.
- **pixel size** : 110 nm
- **light source** : SpectraX multi-LED Lumencor
- **detector model** : Prime 95B A17F203017
- **objective** :
    - **model** : Luminencor Led
    - **immersion** : Oil
    - **lens numerical aperture (NA)** : 1.45
    - **nominal magnification** : 100x
    - **working distance** : 0.130 mm
    - **refractive index** : 1.515

## Raw Image Notes

- **samples per pixel** : 1
- **z planes number** : 13
- **dimension order** : TCZYX
- **images size** : 1, 3, 10, 1200, 1200
- **channel order** : DIC, Mitotracker Far Red, Hoechst
- **type** : uint16
- **format** : .nd2

# Study 19

## Authors


- **Virginie GEORGET**, 1) MRI, BioCampus, University of Montpellier (UM), CNRS, INSERM, Montpellier, France ; 2) CRBM, UM, CNRS, Montpellier, France.
  virginie.georget@mri.cnrs.fr
- **Juliette VAN DIJK**, CRBM, UM, CNRS, Montpellier France
  juliette.van-dijk@crbm.cnrs.fr


- **Role :** JVD prepared the sample, VG made the acquisition.

- **France BioImaging node :** Montpellier
- **France BioImaging platform :** Montpellier Ressources Imagerie (MRI), Centre de Recherche en Biologie cellulaire de Montpellier (CRBM)

## Description

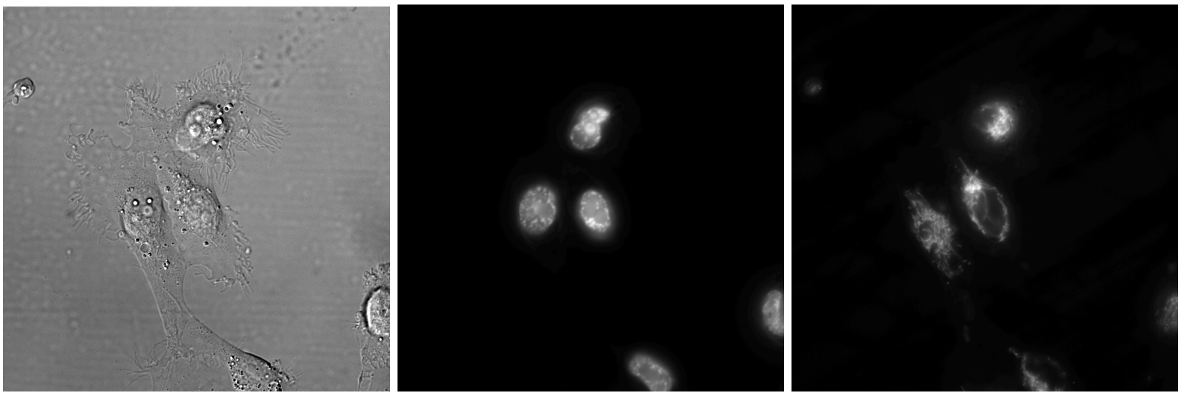

**a)** *image_290_DIC_z4* **b)** *image_290_Nucleus* **c)** *image_290_Mitochondria*

**Fig.study 19:** *Example of images from Study 19.* ***a)*** *DIC microscopy image of U2OS human cells.* ***b)*** *DIC image equivalent but in fluorescence microscopy of H2B-GFP-labeled U2OS human nuclei.* ***c)*** *DIC image equivalent but in fluorescence microscopy of MitoTracker-far-red-labeled U2OS human mitochondria.*

## Study Component

- **name** : U2OS H2BGFP
- **file list access** : json_file tsv_file

## Biosample

- **model** : human / homo sapiens
- **NCBI** : txid9606
- **biological entity** : U2OS stably expressing histone H2B fused to EGFP.
- **ATCC catalog number** : Not Referenced
- **description**: Cells not really healthy. Cells thawed the week before. Not dense.

## Specimen

- **sample preparation** : Cells were plated in glass bottom (#1,5) fluorodish in DMEM. Cells were pre-incubated in HBSS 1X buffer, then HBSS with far-red mitotracker (1/4000) for 20 minutes at 37°C for mitochondria staining. Medium was replaced by fluorobrite and cells were imaged a few hours later.
- **growth protocol** : DMEM Glutamax 10% SVF (Sérum de Veau foetal) (FBS) + 1% PS (penicillin spectromycin).

## Image Data

- **number of images** : 253 with :
  - 207 DIC : image_<*n_img*>_DIC_z<*n_z*>.ome.tiff (where *n_img* from 275 to 297 and *n_z from 0 to 8*)
  - 23 Nucleus : image_<*n_img*>_Nucleus.ome.tiff (where *n_img* from 275 to 297)

    - 23 Mitochondria : image_<*n_img*>_Mitochondria.ome.tiff (where *n_img* from 275 to 297)
- **z planes :**
    - number : 1
    - DIC z focus : 3, 4, 5, 6 or 7 depending on the image
    - Nucleus z focus : 4, 5 or 6 depending on the image
    - Mitochondria z focus : 4 or 5 depending on the image
- **channels** :
    - nucleus :
        - marker : H2B-GFP
        - excitation light : 470/25 nm
        - emission light : 515/30 nm
    - mitochondria :
        - marker : Mitotracker Far Red
        - excitation light : 640/30 nm
        - emission light : 705/65 nm

- **dimension order** : TCZYX
- **images size** : 1, 1, 1, 1200, 1200
- **physical size x** : 0.109929394588707 µm
- **physical size y** : 0.109929394588707 µm
- **physical size z** : 3.0 µm
- **type** : uint16
- **format** : ome.tiff

## Image Acquisition

- **imaging method** : DIC, Fluorescence
- **imaging instrument** : Inverted Nikon Ti2 microscope with temperature and CO2 controlled chamber, CMOS back-illuminated Prime95B Photometrics camera. Motorized Z of the microscope for the Z-stack.
- **pixel size** : 110 nm
- **light source** : SpectraX multi-LED Lumencor
- **detector model** : Prime 95B A17F203017
- **objective** :
    - **model** : Luminencor Led
    - **immersion** : Oil
    - **lens numerical aperture (NA)** : 1.45
    - **nominal magnification** : 100x
    - **working distance** : 0.130 mm
    - **refractive_index** : 1.515

## Raw Image Notes

- **samples per pixel** : 1
- **z planes number** : 9
- **dimension order** : TCZYX
- **images size** : 1, 3, 9, 1200, 1200
- **channel order** : DIC, Mitotracker Far Red, H2B-GFP
- **type** : uint16
- **format** : .nd2

# Study 20

## Authors


- **Virginie GEORGET**, 1) MRI, BioCampus, University of Montpellier (UM), CNRS, INSERM, Montpellier, France ; 2) CRBM, UM, CNRS, Montpellier, France.
  virginie.georget@mri.cnrs.fr
- **Juliette VAN DIJK**, CRBM, UM, CNRS, Montpellier France
  juliette.van-dijk@crbm.cnrs.fr


- **Role :** JVD prepared the sample, VG made the acquisition.

- **France BioImaging node :** Montpellier
- **France BioImaging platform :** Montpellier Ressources Imagerie (MRI), Centre de Recherche en Biologie cellulaire de Montpellier (CRBM)

## Description

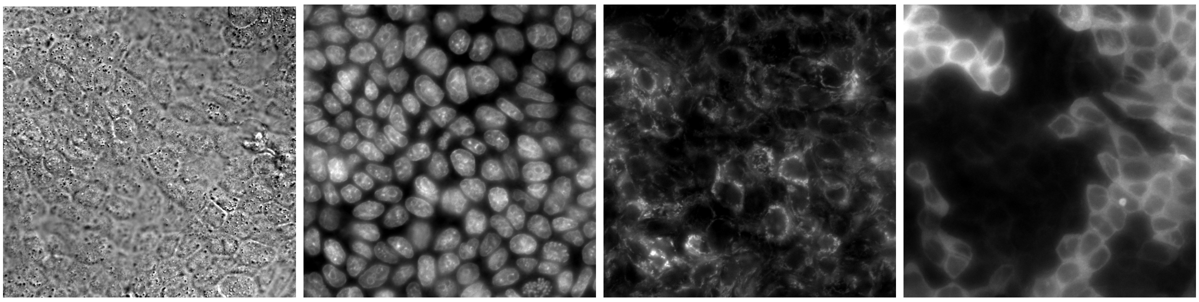

**a)** *image_313_DIC_z6* **b)** *image_313_Nucleus* **c)** *image_313_Mitochondria* **d)** *image_313_Tubulin*

**Fig.study 20:** *Example of images from Study 20.* ***a)*** *DIC microscopy image of MDCK dog cells.* ***b)*** *DIC image equivalent but in fluorescence microscopy of Hoechst-labeled MDCK dog nuclei.* ***c)*** *DIC image equivalent but in fluorescence microscopy of MitoTracker-far-red-labeled MDCK dog mitochondria.* ***d)*** *DIC image equivalent but in fluorescence microscopy of* EB3-Cherry-*labeled MDCK dog tubulin.*

## Study Component

- **name** : Cocker 3
- **file list access** : json_file tsv_file

## Biosample

- **model** : dog / Canis lupus familiaris
- **NCBI** : txid9615
- **biological entity** : MDCK expressing stably the MISP protein fused to Cherry.
- **ATCC catalog number** : Not Referenced
- **description**: Cells isolated from normal kidney tissue. Normal, adult, female cocker spaniel.

## Specimen

- **sample preparation** : Cells were plated in glass bottom (#1,5) 24-well plate. Cells were pre-incubated in HBSS 1X buffer, then HBSS with far-red mitotracker (1/4000) for 20 minutes at 37°C for mitochondria staining. Medium was replaced by fluorobrite and cells were imaged a few hours later adding vital Hoechst (1/4000) a few minutes before.
- **growth protocol** : DMEM Glutamax 10% SVF (Sérum de Veau foetal) (FBS) + 1% PS (penicillin spectromycin).

## Image Data

- **number of images** : 276 with :
  - 207 DIC : image_<*n_img*>_DIC_z<*n_z*>.ome.tiff (where *n_img* from 298 to 320 and *n_z from 0 to 8*)
  - 23 Nucleus : image_<*n_img*>_Nucleus.ome.tiff (where *n_img* from 298 to 320)
  - 23 Mitochondria : image_<*n_img*>_Mitochondria.ome.tiff (where *n_img* from 275 to 297)
  - 23 Tubulin : image_<*n_img*>_Tubulin.ome.tiff (where *n_img* from 298 to 320)

- **z planes :**
    - number : 1
    - DIC z focus : 3, 4 or 5 depending on the image
    - Nucleus z focus : 3, 4 or 5 depending on the image
    - Mitochondria z focus : 2, 3, 4, 5, 6 or 7 depending on the image
    - Tubulin z focus : 3, 4, 5 or 6 depending on the image
- **channels** :
    - nucleus :
        - marker : Hoechst in DAPI
        - excitation light : 395/25 nm
        - emission light : 433/65 nm
    - mitochondria :
        - marker : Mitotracker Far Red
        - excitation light : 640/30 nm
        - emission light : 705/65 nm
    - tubulin :
        - marker : EB3-Cherry in red channel
        - excitation light : 575/25 nm
        - emission light : 632/60 nm

- **dimension order** : TCZYX
- **images size** : 1, 1, 1, 1200, 1200
- **physical size x** : 0.109929394588707 µm
- **physical size y** : 0.109929394588707 µm
- **physical size z** : 3.0 µm
- **type** : uint16
- **format** : ome.tiff

## Image Acquisition

- **imaging method** : DIC, Fluorescence
- **imaging instrument** : Inverted Nikon Ti2 microscope with temperature and $CO_2$ controlled chamber, CMOS back-illuminated Prime95B Photometrics camera. Motorized Z of the microscope for the Z-stack.
- **pixel size** : 110 nm
- **light source** : SpectraX multi-LED Lumencor
- **detector model** : Prime 95B A17F203017
- **objective** :
    - **model** : Luminencor Led
    - **immersion** : Oil
    - **lens numerical aperture (NA)** : 1.45
    - **nominal magnification** : 100x
    - **working distance** : 0.130 mm
    - **refractive index** : 1.515

## Raw Image Notes

- **samples per pixel** : 1
- **z planes number** : 9
- **dimension order** : TCZYX
- **images size** : 1, 4, 9, 1200, 1200
- **channel order** : DIC, EB3-Cherry, Mitotracker Far Red, Hoechst
- **type** : uint16
- **format** : .nd2

# Study 21

## Authors


- **Virginie GEORGET**, 1) MRI, BioCampus, University of Montpellier (UM), CNRS, INSERM, Montpellier, France ; 2) CRBM, UM, CNRS, Montpellier, France.
virginie.georget@mri.cnrs.fr
- **Juliette VAN DIJK**, CRBM, UM, CNRS, Montpellier France
juliette.van-dijk@crbm.cnrs.fr


- **Role :** JVD prepared the sample, VG made the acquisition.

- **France BioImaging node :** Montpellier
- **France BioImaging platform :** Montpellier Ressources Imagerie (MRI), Centre de Recherche en Biologie cellulaire de Montpellier (CRBM)

## Description

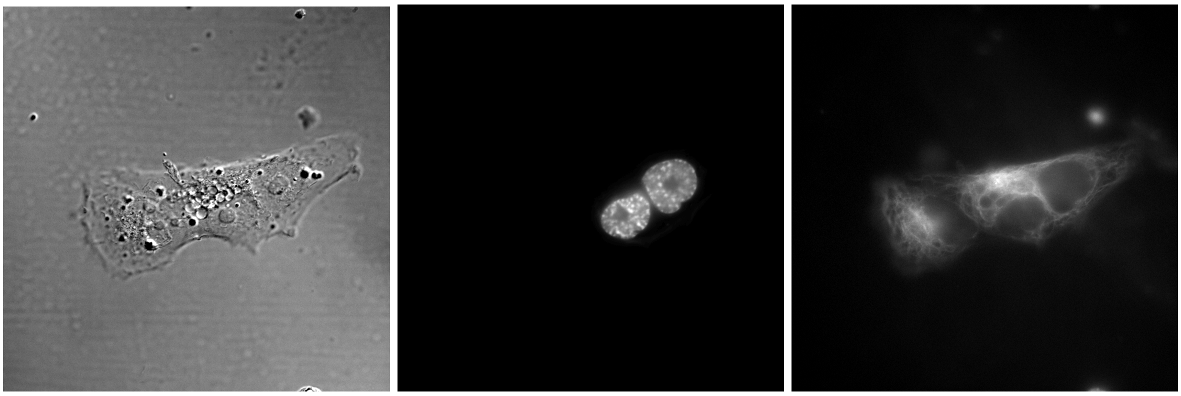

**a)** *image_342_DIC_z5* **b)** *image_342_Nucleus* **c)** *image_342_Tubulin*

**Fig.study 21:** *Example of images from Study 21.* ***a)*** *DIC microscopy image of U2OS human cells.* ***b)*** *DIC image equivalent but in fluorescence microscopy of H2B-GFP-labeled U2OS human nuclei.* ***c)*** *DIC image equivalent but in fluorescence microscopy of SIR-tubulin-labeled U2OS human tubulin.*

## Study Component

- **name** : U2OS H2BGFP
- **file list access** : json_file tsv_file

## Biosample

- **model** : human / homo sapiens
- **NCBI** : txid9606
- **biological entity** : U2OS stably expressing histone H2B fused to EGFP.
- **ATCC catalog number** : Not Referenced
- **description**: Cells not really healthy. Cells thawed the week before. Not dense.

## Specimen

- **sample preparation** : Cells were plated in glass bottom (#1,5) fluorodish in DMEM. Microtubules were stained with SiR Tubulin (1/2000) + Verapamil (1/2000) in fluorobrite for 5 hours at 37°C.
- **growth protocol** : DMEM Glutamax 10% SVF (Sérum de Veau foetal) (FBS)

## Image Data

- **number of images** : 253 with :
  - 207 DIC : image_<*n_img*>_DIC_z<*n_z*>.ome.tiff (where *n_img* from 321 to 343 and *n_z from 0 to 8*)
  - 23 Nucleus : image_<*n_img*>_Nucleus.ome.tiff (where *n_img* from 321 to 343)
  - 23 Tubulin : image_<*n_img*>_Tubulin.ome.tiff (where *n_img* from 321 to 343)
- **z planes :**
  - number : 1

    - DIC z focus : 0, 3, 4, 5, 6, 7 or 8 depending on the image
    - Nucleus z focus : 1, 4, 5, 6, 7 or 8 depending on the image
    - Tubulin z focus : 0, 4, 5 or 6 depending on the image
- **channels** :
    - nucleus :
        - marker : H2B-GFP
        - excitation light : 470/25 nm
        - emission light : 515/30 nm
    - tubulin :
        - marker : SIR-Tubulin
        - excitation light : 640/30 nm
        - emission light : 705/65 nm

- **dimension order** : TCZYX
- **images size** : 1, 1, 1, 1200, 1200
- **physical size x** : 0.109929394588707 µm
- **physical size y** : 0.109929394588707 µm
- **physical size z** : 3.0 µm
- **type** : uint16
- **format** : ome.tiff

## Image Acquisition

- **imaging method** : DIC, Fluorescence
- **imaging instrument** : Inverted Nikon Ti2 microscope with temperature and $CO_2$ controlled chamber, CMOS back-illuminated Prime95B Photometrics camera. Motorized Z of the microscope for the Z-stack.
- **pixel size** : 110 nm
- **light source** : SpectraX multi-LED Lumencor
- **detector model** : Prime 95B A17F203017
- **objective** :
    - **model** : Luminencor Led
    - **immersion** : Oil
    - **lens numerical aperture (NA)** : 1.45
    - **nominal magnification** : 100x
    - **working distance** : 0.130 mm
    - **refractive index** : 1.515

## Raw Image Notes

- **samples per pixel** : 1
- **z planes number** : 9
- **dimension order** : TCZYX
- **images size** : 1, 3, 9, 1200, 1200
- **channel order** : DIC, H2B-GFP, SIR-Tubulin
- **type** : uint16
- **format** : .nd2

# Study 22

## Authors


- **Virginie GEORGET**, 1) MRI, BioCampus, University of Montpellier (UM), CNRS, INSERM, Montpellier, France ; 2) CRBM, UM, CNRS, Montpellier, France. virginie.georget@mri.cnrs.fr
- **Juliette VAN DIJK**, CRBM, UM, CNRS, Montpellier France juliette.van-dijk@crbm.cnrs.fr


- **Role :** JVD prepared the sample, VG made the acquisition.

- **France BioImaging node :** Montpellier
- **France BioImaging platform :** Montpellier Ressources Imagerie (MRI), Centre de Recherche en Biologie cellulaire de Montpellier (CRBM)

## Description

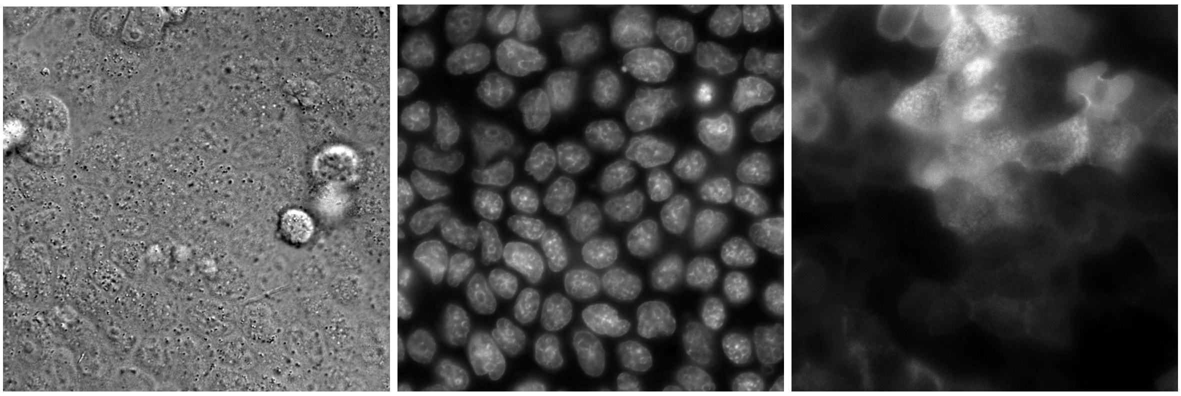

**a)** *image_358_DIC_z3* **b)** *image_358_Nucleus* **c)** *image_358_Mitochondria*

**Fig.study 22:** *Example of images from Study 22.* ***a)*** *DIC microscopy image of MDCK dog cells.* ***b)*** *DIC image equivalent but in fluorescence microscopy of Hoechst-labeled MDCK dog nuclei.* ***c)*** *DIC image equivalent but in fluorescence microscopy of MISP-Cherry-labeled MDCK dog mitochondria.*

## Study Component

- **name** : Cocker 2
- **file list access** : json_file tsv_file

## Biosample

- **model** : dog / Canis lupus familiaris
- **NCBI** : txid9615
- **biological entity** : MDCK expressing stably the MISP protein fused to Cherry.
- **ATCC catalog number** : Not Referenced
- **description**: Cells isolated from normal kidney tissue. Normal, adult, female cocker spaniel.

## Specimen

- **sample preparation** : Cells were plated in glass bottom (#1,5) 24-well plate. Cells were pre-incubated in HBSS 1X buffer, then HBSS with far-red mitotracker (1/4000) for 20 minutes at 37°C for mitochondria staining. Medium was replaced by fluorobrite and cells were imaged a few hours later adding vital Hoechst (1/4000) a few minutes before.
- **growth protocol** : DMEM Glutamax 10% SVF (Sérum de Veau foetal) (FVS) + 1% PS (penicillin spectromycin).

## Image Data

- **number of images** : 253 with :
  - 207 DIC : image_<*n_img*>_DIC_z<*n_z*>.ome.tiff (where *n_img* from 344 to 366 and *n_z from 0 to 8*)
  - 23 Nucleus : image_<*n_img*>_Nucleus.ome.tiff (where *n_img* from 344 to 366)
  - 23 Mitochondria : image_<*n_img*>_Mitochondria.ome.tiff (where *n_img* from 344 to 366)

- **z planes :**
  - number : 1
  - DIC z focus : 4 or 5 depending on the image
  - Nucleus z focus : 4 or 5 depending on the image
  - Mitochondria z focus : 4, 5 or 6 depending on the image
- **channels** :
  - nucleus :
    - marker : Hoechst in DAPI
    - excitation light : 395/25 nm
    - emission light : 433/65 nm
  - mitochondria :
    - marker : MISP-Cherry in red channel
    - excitation light : 575/25 nm
    - emission light : 632/60 nm

- **dimension order** : TCZYX
- **images size** : 1, 1, 1, 1200, 1200
- **physical size x** : 0.109929394588707 µm
- **physical size y** : 0.109929394588707 µm
- **physical size z** : 3.0 µm
- **type** : uint16
- **format** : ome.tiff

## Image Acquisition

- **imaging method** : DIC, Fluorescence
- **imaging instrument** : Inverted Nikon Ti2 microscope with temperature and $CO_2$ controlled chamber, CMOS back-illuminated Prime95B Photometrics camera. Motorized Z of the microscope for the Z-stack.
- **pixel size** : 110 nm
- **light source** : SpectraX multi-LED Lumencor
- **detector model** : Prime 95B A17F203017
- **objective** :
  - **model** : Luminencor Led
  - **immersion** : Oil
  - **lens numerical aperture (NA)** : 1.45
  - **nominal magnification** : 100x
  - **working distance** : 0.130 mm
  - **refractive index** : 1.515

## Raw Image Notes

- **samples per pixel** : 1
- **z planes number** : 13
- **dimension order** : TCZYX
- **images size** : 1, 3, 13, 1200, 1200
- **channel order** : DIC, MISP-Cherry, H2B-GFP
- **type** : uint16
- **format** : .nd2

# Study 23

## Authors

- **Virginie GEORGET**, 1) MRI, BioCampus, University of Montpellier (UM), CNRS, INSERM, Montpellier, France ; 2) CRBM, UM, CNRS, Montpellier, France. virginie.georget@mri.cnrs.fr
- **Juliette VAN DIJK**, CRBM, UM, CNRS, Montpellier France juliette.van-dijk@crbm.cnrs.fr

- **Role :** JVD prepared the sample, VG made the acquisition.

- **France BioImaging node :** Montpellier
- **France BioImaging platform :** Montpellier Ressources Imagerie (MRI), Centre de Recherche en Biologie cellulaire de Montpellier (CRBM)

## Description

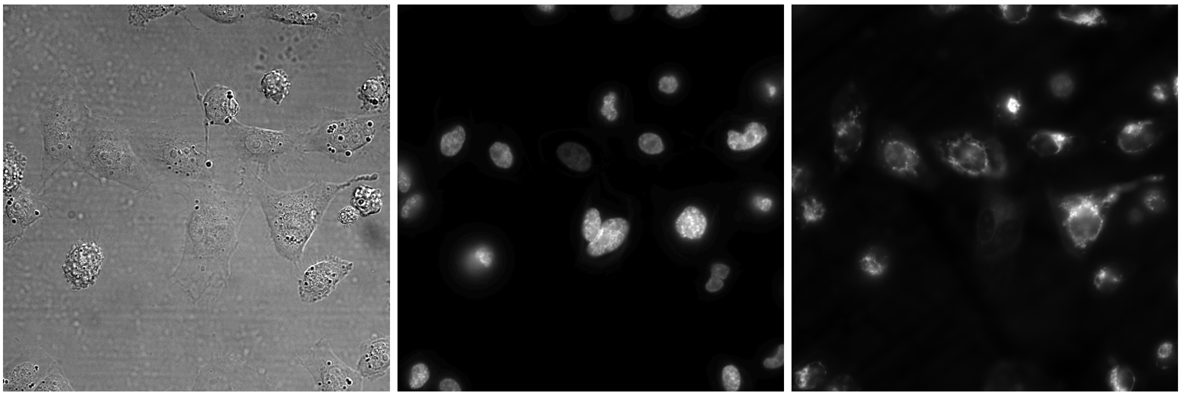

**a)** *image_388_DIC_z5* **b)** *image_388_Nucleus* **c)** *image_388_Mitochondria*

**Fig.study 23:** *Example of images from Study 23.* ***a)*** *DIC microscopy image of U2OS human cells.* ***b)*** *DIC image equivalent but in fluorescence microscopy of H2B-GFP-labeled U2OS human nuclei.* ***c)*** *DIC image equivalent but in fluorescence microscopy of MitoTracker-far-red-labeled U2OS human mitochondria.*

## Study Component

- **name** : U2OS H2BGFP 2
- **file list access** : json_file tsv_file

## Biosample

- **model** : human / homo sapiens
- **NCBI** : txid9606
- **biological entity** : U2OS stably expressing histone H2B fused to EGFP.
- **ATCC catalog number** : Not Referenced
- **description**: Cells not really healthy. Cells thawed the week before. Not dense.

## Specimen

- **sample preparation** : Cells were plated in glass bottom (#1,5) fluorodish in DMEM. Cells were pre-incubated in HBSS 1X buffer, then HBSS with far-red mitotracker (1/4000) for 20 minutes at 37°C for mitochondria staining. Medium was replaced by fluorobrite and cells were imaged a few hours later.
- **growth protocol** : DMEM Glutamax 10% SVF (Sérum de Veau foetal)

## Image Data

- **number of images** : 253 with :
  - 207 DIC : image_<*n_img*>_DIC_z<*n_z*>.ome.tiff (where *n_img* from 367 to 389 and *n_z from 0 to 8*)
  - 23 Nucleus : image_<*n_img*>_Nucleus.ome.tiff (where *n_img* from 367 to 389)
  - 23 Mitochondria : image_<*n_img*>_Mitochondria.ome.tiff (where *n_img* from 367 to 389)
- **z planes :**

    - number : 1
    - DIC z focus : 0, 1, 2, 8 depending on the image
    - Nucleus z focus : 4 or 5 depending on the image
    - Mitochondria z focus : 4
- **channels** :
    - nucleus :
        - marker : H2B-GFP
        - excitation light : 470/25 nm
        - emission light : 515/30 nm
    - mitochondria :
        - marker : Mitotracker Far Red
        - excitation light : 640/30 nm
        - emission light : 705/65 nm

- **dimension order** : TCZYX
- **images size** : 1, 1, 1, 1200, 1200
- **physical size x** : 0.182636731499817 µm
- **physical size y** : 0.182636731499817 µm
- **physical size z** : 3.0 µm
- **type** : uint16
- **format** : ome.tiff

## Image Acquisition

- **imaging method** : DIC, Fluorescence
- **imaging instrument** : Inverted Nikon Ti2 microscope with temperature and $CO_2$ controlled chamber, CMOS back-illuminated Prime95B Photometrics camera. Motorized Z of the microscope for the Z-stack.
- **pixel size** : 182 nm
- **light source** : SpectraX multi-LED Lumencor
- **detector model** : Prime 95B A17F203017
- **objective** :
    - **model** : Luminencor Led
    - **immersion** : Oil
    - **lens numerical aperture (NA)** : 1.4
    - **nominal magnification** :  60x
    - **working distance** : 0.130 mm
    - **refractive index** : 1.515

## Raw Image Notes

- **samples per pixel** : 1
- **z planes number** : 9
- **dimension order** : TCZYX
- **images size** : 1, 3, 9, 1200, 1200
- **channel order** : DIC, H2B-GFP, Mitotracker Far Red
- **type** : uint16
- **format** : .nd2

# Study 24

## Authors


- **Virginie GEORGET**, 1) MRI, BioCampus, University of Montpellier (UM), CNRS, INSERM, Montpellier, France ; 2) CRBM, UM, CNRS, Montpellier, France. virginie.georget@mri.cnrs.fr
- **Juliette VAN DIJK**, CRBM, UM, CNRS, Montpellier France juliette.van-dijk@crbm.cnrs.fr


- **Role :** JVD prepared the sample, VG made the acquisition.

- **France BioImaging node :** Montpellier
- **France BioImaging platform :** Montpellier Ressources Imagerie (MRI), Centre de Recherche en Biologie cellulaire de Montpellier (CRBM)

## Description

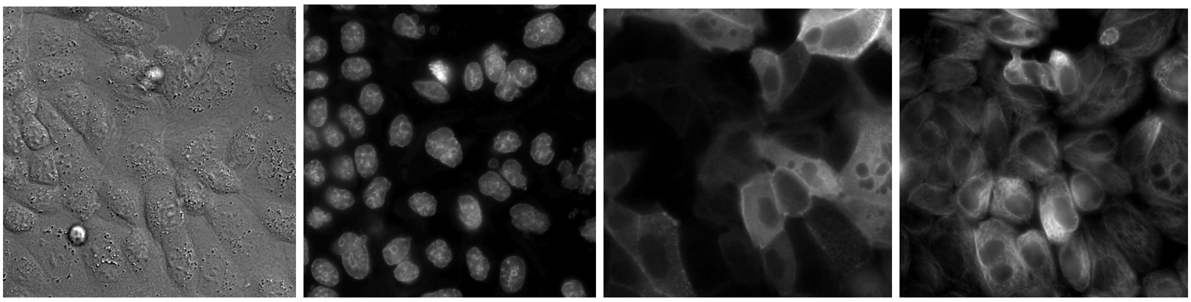

**a)** *image_399_DIC_z4* **b)** *image_399_Nucleus* **c)** *image_399_Mitochondria* **d)** *image_399_Tubulin*

**Fig.study 24:** *Example of images from Study 24.* ***a)*** *DIC microscopy image of MDCK dog cells.* ***b)*** *DIC image equivalent but in fluorescence microscopy of Hoechst-labeled MDCK dog nuclei.* ***c)*** *DIC image equivalent but in fluorescence microscopy of MitoTracker-far-red-labeled MDCK dog mitochondria.* ***d)*** *DIC image equivalent but in fluorescence microscopy of* EB3-Cherry-*labeled MDCK dog tubulin.*

## Study Component

- **name** : Cocker 4
- **file list access** : json_file tsv_file

## Biosample

- **model** : dog / Canis lupus familiaris
- **NCBI** : txid9615
- **biological entity** : MDCK expressing stably the MISP protein fused to Cherry.
- **ATCC catalog number** : Not Referenced
- **description**: Cells isolated from normal kidney tissue. Normal, adult, female cocker spaniel.

## Specimen

- **sample preparation** : Cells were plated in glass bottom (#1,5) 24-well plate. Microtubules were stained with SiR Tubulin (1/2000) + Verapamil (1/2000) in fluorobrite for 5 hours at 37°C and cells were imaged adding vital Hoechst (1/4000) a few minutes before.
- **growth protocol** : DMEM Glutamax 10% SVF (Sérum de Veau foetal) (FVS) + 1% PS (penicillin spectromycin).

## Image Data

- **number of images** : 276 with :
  - 207 DIC : image_<*n_img*>_DIC_z<*n_z*>.ome.tiff (where *n_img* from 390 to 412 and *n_z from 0 to 8*)
  - 23 Nucleus : image_<*n_img*>_Nucleus.ome.tiff (where *n_img* from 390 to 412)
  - 23 Mitochondria : image_<*n_img*>_Mitochondria.ome.tiff (where *n_img* from 390 to 412)
  - 23 Tubulin : image_<*n_img*>_Tubulin.ome.tiff (where *n_img* from 390 to 412)
- **z planes :**
  - number : 1

    - DIC z focus : 4 or 5 depending on the image
    - Nucleus z focus : 4
    - Mitochondria z focus : 4, 5, 6 or 7 depending on the image
    - Tubulin z focus : 4, 5, 6 or 7 depending on the image
- **channels** :
    - nucleus :
        - marker : Hoechst in DAPI
        - excitation light : 395/25 nm
        - emission light : 433/65 nm
    - mitochondria :
        - marker : MISP-Cherry in red
        - excitation light : 575/25 nm
        - emission light : 632/60 nm
    - tubulin :
        - marker : SIR tubulin in FarRed
        - excitation light : 640/30 nm
        - emission light : 705/65 nm

- **dimension order** : TCZYX
- **images size** : 1, 1, 1, 1200, 1200
- **physical size x** : 0.109929394588707 µm
- **physical size y** : 0.109929394588707 µm
- **physical size z** : 3.0 µm
- **type** : uint16
- **format** : ome.tiff

## Image Acquisition

- **imaging method** : DIC, Fluorescence
- **imaging instrument** : Inverted Nikon Ti2 microscope with temperature and CO2 controlled chamber, CMOS back-illuminated Prime95B Photometrics camera. Motorized Z of the microscope for the Z-stack.
- **pixel size** : 110 nm
- **light source** : SpectraX multi-LED Lumencor
- **detector model** : Prime 95B A17F203017
- **objective** :
    - **model** : Luminencor Led
    - **immersion** : Oil
    - **lens numerical aperture (NA)** : 1.45
    - **nominal magnification** : 100x
    - **working distance** : 0.130 mm
    - **refractive index** : 1.515

## Raw Image Notes

- **samples per pixel** : 1
- **z planes number** : 9
- **dimension order** : TCZYX
- **images size** : 1, 4, 9, 1200, 1200
- **channel order** : DIC, MISP-Cherry, SIR tubulin, H2B-GFP
- **type** : uint16
- **format** : .nd2

# Study 25

## Authors


- **Oriane POURCELOT**, IGH, UM, CNRS, Montpellier, France. oriane.pourcelot@igh.cnrs.fr
- **Julio MATEOS-LANGERAK**, MRI, BioCampus, University of Montpellier (UM), CNRS, INSERM, Montpellier, France. IGH, UM, CNRS, Montpellier, France. julio.mateos-langerak@igh.cnrs.fr


- **Role :** OP prepared the sample and JML made the acquisition

- **France BioImaging node :** Montpellier
- **France BioImaging platform :** Institut de Génétique Humaine (IGH), Montpellier Ressources Imagerie (MRI)

## Description

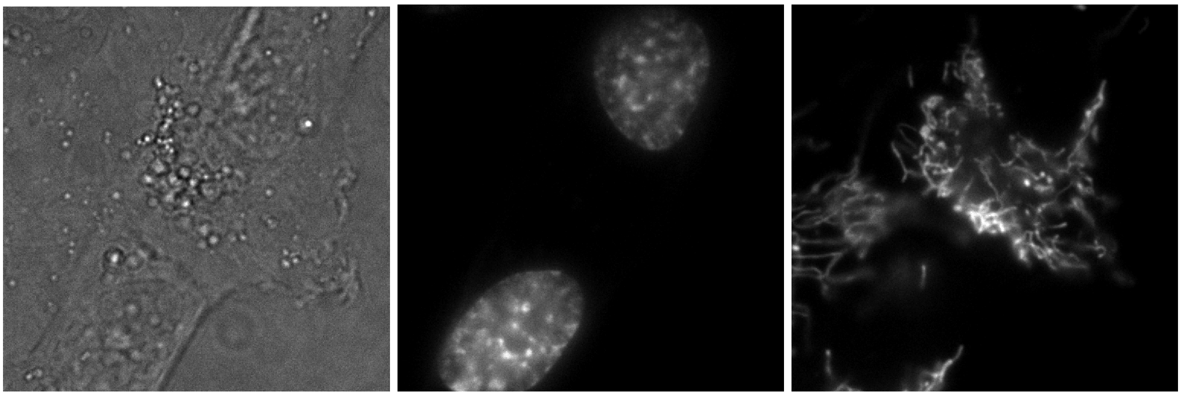

**a)** *image_999_BF_z9* **b)** *image_999_Nucleus* **c)** *image_999_Mitochondria*

**Fig.study 25:** *Example of images from Study 25.* ***a)*** *BF microscopy image of HeLa human cells.* ***b)*** *BF image equivalent but in fluorescence microscopy of Hoechst-labeled human HeLa HeLa nuclei.* ***c)*** *BF image equivalent but in fluorescence microscopy of MitoTracker-labeled human HeLa HeLa mitochondria.*

## Study Component

- **name** : HeLa IGH
- **file list access** : json_file tsv_file

## Biosample

- **model** : human / homo sapiens
- **NCBI** : txid9606
- **biological entity** : HeLa stably expressing RAB6A fused with EGFP
- **ATCC catalog number** : CCL-2
- **description**: HeLa cells are the first immortal human cells to be grown in culture and the basis for countless significant scientific discoveries. They were isolated in 1,951 from a cervical carcinoma derived from a 31-year-old patient.

## Specimen

- **sample preparation** : Cells were plated in 35 mm glass bottom dish #1,5 in DMEM. Mitochondria were stained with MitoTracker Deep Red (1/4,000) in HBSS for 30 min at 37°C and the DNA was stained with Hoechst (20 µg/ml) in PBS for 30 min at 37°C.
- **growth protocol** : cell culture medium DMEM + 10% FBS + 1% pen/strep.

## Image Data

- **number of images** : 29,647 with :
  - 27,069 BF : image_<*img_num*>_BF_z<*z_num*>.ome.tiff (where *num_img* from 413 to 1,701 and *z_num from 0 to 33*)
  - 1,289 Nucleus : image_<*img_num*>_Nucleus.ome.tiff (where *img_num* from 413 to 1,701)

        - 1,289 Mitochondria : image_<*img_num*>_Mitochondria.ome.tiff (where *img_num* from 413 to 1,701)
- **z planes :**
    - number : 1
    - BF z focus : from 0 to 20 depending on the image
    - Nucleus z focus : from 0 to 20 depending on the image
    - Mitochondria z focus : from 0 to 20 depending on the image
- **channels** :
    - nucleus :
        - marker : Hoechst
        - excitation light :405 nm
        - emission light : 436/31 nm
    - mitochondria :
        - marker : MitoTracker far red
        - excitation light : 642 nm
        - emission light : 683/40 nm

- **dimension order** : TCZYX
- **images size** : 1, 1, 1, 512, 512
- **physical size x** : 0.0799999982118606 µm
- **physical size y** : 0.0799999982118606 µm
- **physical size z** : 1.0 µm
- **type** : uint16
- **format** : ome.tiff

## Image Acquisition

- **imaging method** : BF, Fluorescence
- **imaging instrument** : Deltavision OMX-V4, temperature and gas controlled.
- **pixel size** : 79 nm
- **light source** : Vortran Stradus (wavelength : 405 nm), unknown (wavelength : 642 nm)
- **detector model** : EMCCD Evolve 512B photometric camera
- **objective** :
    - **model** : Olympus 1-U2B836
    - **immersion** : Oil
    - **lens numerical aperture (NA)** : 1.4
    - **nominal magnification** : 100x
    - **working distance** : 0.12 mm
    - **refractive index** : 1.518

## Raw Image Notes

- **samples per pixel** : 1
- **z planes number** : 21
- **dimension order** : TCZYX
- **images size** : 1, 3, 21, 512, 512
- **channel order** : MitoTracker far red, BF, Hoechst
- **type** : uint16
- **format** : .dv

# Study 26

## Authors


- **Oriane POURCELOT**, CNRS, IGH, UMR9002, Montpellier, France
  oriane.pourcelot@igh.cnrs.fr
- **Julio MATEOS-LANGERAK**, MRI, BioCampus, University of Montpellier (UM), CNRS, INSERM, Montpellier, France. IGH, UM, CNRS, Montpellier, France.
  julio.mateos-langerak@igh.cnrs.fr


- **Role :** OP prepared the sample and JML made the acquisition

- **France BioImaging node :** Montpellier
- **France BioImaging platform :** Institut de Génétique Humaine (IGH), Montpellier Ressources Imagerie (MRI)

## Description

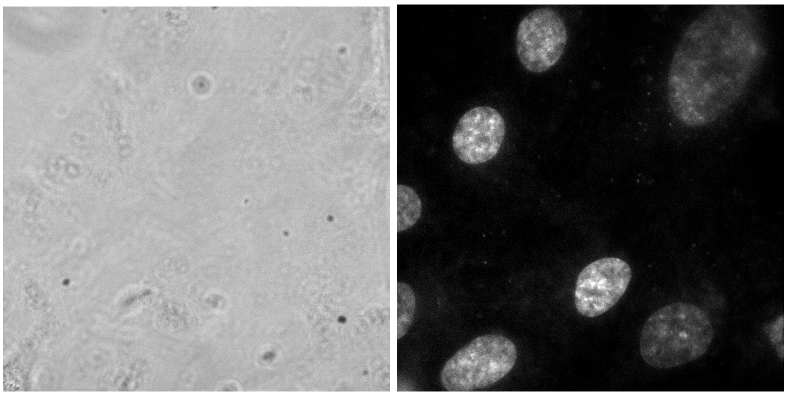

**a)** *image_1718_BF_z19* **b)** *image_1718_Nucleus*

**Fig.study 26:** *Example of images from Study 26.* ***a)*** *BF microscopy image of HeLa human cells.* ***b)*** *BF image equivalent but in fluorescence microscopy of Hoechst-labeled human HeLa nuclei.*

## Study Component

- **name** : HeLa IGH 2
- **file list access** : json_file tsv_file

## Biosample

- **model** : human / homo sapiens
- **NCBI** : txid9606
- **biological entity** : HeLa stably expressing RAB6A fused with EGFP
- **ATCC catalog number** : CCL-2
- **description**: HeLa cells are the first immortal human cells to be grown in culture and the basis for countless significant scientific discoveries. They were isolated in 1951 from a cervical carcinoma derived from a 31-year-old patient.

## Specimen

- **sample preparation** : Cells were plated in 35mm glass bottom dish #1,5 in DMEM. The DNA was dyed with SiR DNA in far red (1 µM) in PBS for 1h at 37°C.
- **growth protocol** : Cell culture medium DMEM + 10% FBS + 1% pen/strep.

## Image Data

- **number of images** : 13,501 with :
  - 12,914 BF : image_<*img_num*>_BF_z<*z_num*>.ome.tiff (where *num_img* from 1,702 to 2,288 and *z_num from 0 to 21*)
  - 587 Nucleus : image_<*img_num*>_Nucleus.ome.tiff (where *img_num* from 1,702 to 2,288)
- **z planes :**
  - number : 1

    - BF z focus : from 10 to 20, 0, 1 or 9 depending on the image
    - Nucleus z focus : from 7 to 21 depending on the image
- **channels** :
    - nucleus :
        - marker : SiR DNA-647
        - excitation light : 647 nm
        - emission light : 685/40 nm
        - emission light :

- **dimension order** : TCZYX
- **images size** : 1, 1, 1 980, 1016
- **physical size x** : 0.102 µm
- **physical size y** : 0.102 µm
- **physical size z** : 1.0 µm
- **type** : uint16
- **format** : ome.tiff

## Image Acquisition

- **imaging method** : BF, Fluorescence
- **imaging instrument** : Home-made microscope, OMX-t. Temperature and gas controlled.
- **pixel size** : 102 nm
- **light source** : Coherent OBIS 200nW (wavelength : 647 nm)
- **detector model** : Andor ZYLA-4.2-CL10-W (for SIR-DNA), Andor ZYLA-4.2P-CL10-W (for BF)
- **objective** :
    - **model** : Olympus 60 UPLSAPO 60xO
    - **immersion** : Oil
    - **lens numerical aperture (NA)** : 1.42
    - **nominal magnification** : 60x
    - **working distance** : 0.15 mm
    - **refractive index** : 1.518

## Raw Image Notes

- **samples per pixel** : 1
- **z planes number** : 22
- **dimension order** : TCZYX
- **images size** : 1, 2, 22, 980, 1016
- **channel order** : BF, SiR DNA-647
- **type** : uint16
- **format** : ome.tif

# Study 27

## Authors


- **Oriane POURCELOT**, CNRS, IGH, UMR9002, Montpellier, France
  oriane.pourcelot@igh.cnrs.fr


- **Role :** OP prepared the sample and JML made the acquisition

- **France BioImaging node :** Montpellier
- **France BioImaging platform :** Institut de Génétique Humaine (IGH)

## Description

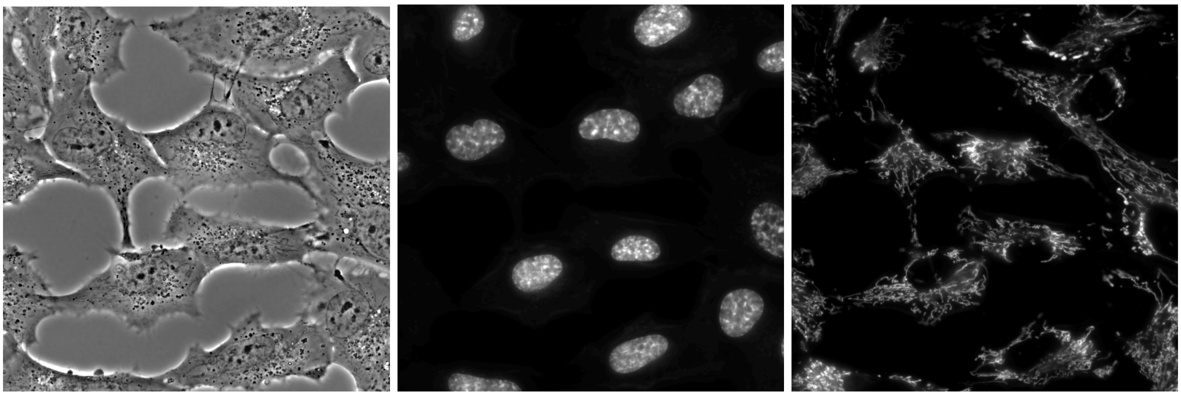

**a)** *image_2289_PC_z17* **b)** *image_2289_Nucleus* **c)** *image_2289_Mitochondria*

**Fig.study 27:** *Example of images from Study 27.* ***a)*** *PC microscopy image of HeLa human cells.* ***b)*** *PC image equivalent but in fluorescence microscopy of Hoechst-labeled human HeLa nuclei.* ***c)*** *PC image equivalent but in fluorescence microscopy of MitoTracker-labeled human HeLa mitochondria.*

## Study Component

- **name** : HeLa IGH 3
- **file list access** : json_file tsv_file

## Biosample

- **model** : human / homo sapiens
- **NCBI** : txid9606
- **biological entity** : HeLa stably expressing RAB6A fused with EGFP
- **ATCC catalog number** : CCL-2
- **description**: HeLa cells are the first immortal human cells to be grown in culture and the basis for countless significant scientific discoveries. They were isolated in 1951 from a cervical carcinoma derived from a 31-year-old patient.

## Specimen

- **sample preparation** : Cells were plated on 25x25 coverslips in DMEM. Mitochondria were stained with MitoTracker Deep Red (1/4000) in HBSS for 30min at 37°C and the DNA was stained with Hoechst (20µg/ml) in PBS for 30min at 37°C.
- **growth protocol** : Cell culture medium DMEM + 10% FBS + 1% pen/strep.

## Image Data

- **number of images** : 6,269
  - 5,875 PC : image_<*img_num*>*_PC_z*<*z_num*>.ome.tiff (where *num_img* from 2,289 to 2,485 and *z_num from 0 to 33*)
  - 197 Nucleus : image_<*img_num*>_Nucleus.ome.tiff (where *img_num* from 2,289 to 2,485)
  - 197 Mitochondria : image_<*img_num*>_Mitochondria.ome.tiff (where *img_num* from 2,289 to 2,485)
- **z planes :**
  - number : 1
  - PC z focus : 0, 1, 2, from 6 to 23, 25 or 29 depending on the image

    - Nucleus z focus : 0, 1, 2, 3, from 6 to 29 or 32 depending on the image
    - Mitochondria z focus : 0, 1, 2, from 6 to 23, 25 or 29  depending on the image
- **channels** :
    - nucleus :
        - marker : Hoechst (in DAPI)
        - excitation light : 360/40 nm
        - emission light : 470/40 nm
    - mitochondria :
        - marker : MitoTracker far red
        - excitation light : 620/60 nm
        - emission light : 700/75 nm

- **dimension order** : TCZYX
- **images size** : 1, 1, 1 980, 1016
- **physical size x** : 0.102 µm
- **physical size y** : 0.102 µm
- **physical size z** : 1.0 µm
- **type** : uint16
- **format** : ome.tiff

## Image Acquisition

- **imaging method** : PC, Fluorescence
- **imaging instrument** : Leica THUNDER
- **pixel size** : 65 nm
- **light source** : Fluo LED SOLA (DAPI Ex360/40, FT400, Em470/40), Fluo LED SOLA (Cy5 Ex620/60, FT660, Em700/75)
- **detector model** : Camera Leica K8 sCMOS - 4.2MP
- **objective** :
    - **model** : Leica 100X/1.4 Oil HC PL APO DIC Ph
    - **immersion** : Oil
    - **lens numerical aperture (NA)** : 1.4
    - **nominal magnification** : 100x
    - **working distance** : 0.09 mm
    - **refractive index** : 1.515

## Raw Image Notes

- **samples per pixel** : 1
- **z planes number** : 24 to 33
- **dimension order** : TCZYX
- **images size** : 1, 3, 24-33, 980, 1016
- **channel order** : MitoTracker far red, BF, Hoechst
- **type** : uint16
- **format** : .lif

# Study 28

## Authors


- **Jean-Bernard FICHE**, CBS, CNRS UMR 5048, UM, INSERM U 1054, Montpellier, France fiche@cbs.cnrs.fr
- **David LLERES**, CBS, CNRS UMR 5048, UM, INSERM U 1054, Montpellier, France david.lleres@cbs.cnrs.fr
- **Christine DOUCET**, CBS, CNRS, UMR 5048, UM, INSERM U 1054, Montpellier, France doucet@cbs.cnrs.fr


- **Role :** CD and DL prepared the samples, JBF and DL acquired the data.

- **France Bio-Imaging node :** Montpellier
- **France Bio-Imaging platform :** Centre de Biologie Structurale (CBS)

## Description

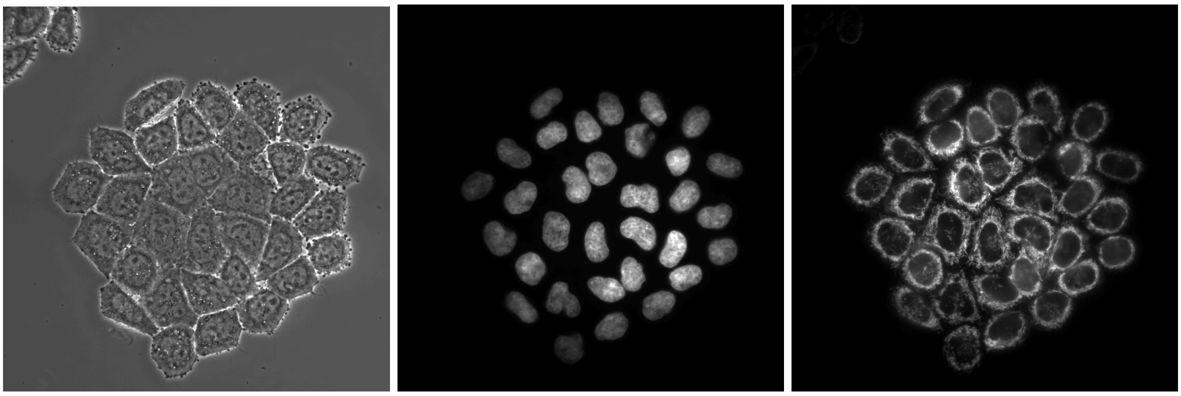

**a)** *image_2491_PC_z10* **b)** *image_2491_Nucleus* **c)** *image_2491_Mitochondria*

**Fig.study 28:** *Example of images from Study 28.* ***a)*** *PC microscopy image of C4-2B human prostate cells.* ***b)*** *PC image equivalent but in fluorescence microscopy of Hoechst-labeled C4-2B human prostate nuclei.* ***c)*** *PC image equivalent but in fluorescence microscopy of MitoTracker-labeled C4-2B human prostate mitochondria.*

## Study Component

- **name** : Scan Hela H2B GFP
- **file list access** : json_file tsv_file

## Biosample

- **model** : human / homo sapiens
- **NCBI** : txid9606
- **biological entity** : HeLa cells expressing stably histone H2B fused to EGFP
- **ATCC catalog number** : ATCC CRL-3315A
- **description**: HeLa cells are derived from Human papillomavirus-related cervical adenocarcinoma. This cell line is expressing stably histone H2B fused to EGFP. (see Lleres et al, JCB, 2009 as reference).

## Specimen

- **sample preparation** : cells were plated in glass coverslips. Labeling 15 min before imaging with Mitotracker red for mitochondria.
- **growth protocol** : n.a.n

## Image Data

- **number of images** : 344 with :
  - 320 PC : image_<*img_num*>_BF_z<*z_num*>.ome.tiff (where *num_img* from 2,486 to 2,501 and *z_num from 0 to 19*)
  - 8 Nucleus : image_<*img_num*>_Nucleus.ome.tiff (where *img_num* from 2,486 to 2,501)
  - 16 Mitochondria : image_<*img_num*>_Mitochondria.ome.tiff (where *img_num* from 2,486 to 2,501)

- **z planes :**
  - number : 1
  - PC z focus : from 0 to 19 depending on the image
  - Nucleus z focus : 7, 9, 10, 11, 12, 14 depending on the image
  - Mitochondria z focus : from 2 to 10 depending on the image
- **channels** :
  - nucleus :
    - marker : H2B-GFP (marquage histone H2b)
    - excitation wavelength : 488 nm
    - emission wavelength : 525/50 nm
  - mitochondria :
    - marker : Cyto-red
    - excitation light : 561 nm
    - emission light : 600/40 nm

- **dimension order** : TCZYX
- **images size** : 1, 1, 1, 2048, 2048
- **physical size x** : 0.106 µm
- **physical size y** : 0.106 µm
- **physical size z** : 0.4 µm
- **type** : uint16
- **format** : ome.tiff

## Image Acquisition

- **imaging method** : PC, Fluorescence
- **imaging instrument** : Home-made setup equipped with a ORCA Flash 4 sCMOS (Hamamatsu) and a 60x Olympus phase contrast objective.
- **pixel size** : 106 nm
- **light source** : OBIS 488 nm (Coherent, max power 100 mW), Sapphire 561 nm (Coherent, max power 150 mW)
- **detector model** : Hamamatsu C11440-22C SN:000242
- **objective** :
  - **model** : Olympus UP plan FL N
  - **immersion** : Oil
  - **lens numerical aperture (NA)** : 1.25
  - **nominal magnification** : 60x
  - **working distance** : 0.17 mm
  - **refractive index** : 1.515

## Raw Image Notes

- **samples per pixel** : 1
- **z planes number** : 20
- **dimension order** : TCZYX
- **images size** : 1, 1, 20, 2048, 2048
- **channel order** : PC, Cyto-red, H2B-GFP
- **type** : uint16
- **format** : .tif

# Study 29

## Authors


- **Valentin ASEI-CESCHINO**, CNRS, MRI, IGH, CRBM, France
  valentin.asei-ceschino@igh.cnrs.fr


- **Role :** VAC prepared the sample and made the acquisition.

- **France BioImaging node :** Montpellier
- **France BioImaging platform :** Montpellier Ressources Imagerie (MRI), Institut de Génétique Humaine (IGH), Centre de Recherche en Biologie cellulaire de Montpellier (CRBM)

## Description

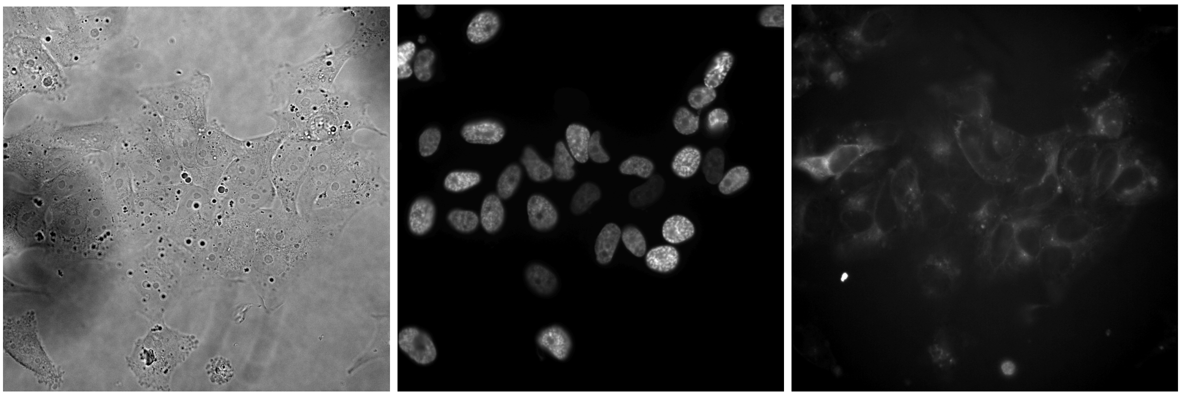

**a)** *image_2516_PC_z1* **b)** *image_2516_Nucleus* **c)** *image_2516_Tubulin*

**Fig.study 29:** *Example of images from Study 29.* ***a)*** *PC microscopy image of U2OS human cells.* ***b)*** *PC image equivalent but in fluorescence microscopy of H2B-GFP-labeled U2OS human nuclei.* ***c)*** *PC image equivalent but in fluorescence microscopy of SiR-tubulin-labeled U2OS human tubulin.*

## Study Component

- **name** : U2OS IGH 2
- **file list access** : json_file tsv_file

## Biosample

- **model** : human / homo sapiens
- **NCBI** : txid9606
- **biological entity** : U2OS stably expressing histone H2B fused to EGFP.
- **ATCC catalog number** : Not Referenced
- **description**: Cells not really healthy. Cells thawed the week before. Not dense.

## Specimen

- **sample preparation** : Cells were plated in glass bottom (#1,5) fluorodish in DMEM. Microtubules were stained with SiR Tubulin (1/2000) + Verapamil (1/2000) in fluorobrite for 5 hours at 37°C.
- **growth protocol** : DMEM Glutamax 10% SVF (Sérum de Veau foetal)

## Image Data

- **number of images** : 801 with :
  - 705 PC : image_<*n_img*>_PC_z<*n_z*>.ome.tiff (where *n_img* from 2,502 to 2,549 and *n_z from 0 to 20*)
  - 48 Nucleus : image_<*n_img*>_Nucleus.ome.tiff (where *n_img* from 2,502 to 2,549)
  - 48 Tubulin : image_<*n_img*>_Tubulin.ome.tiff (where *n_img* from 2,502 to 2,549)
- **z planes :**
  - number : 1
  - PC z focus : 0, 3, 4, 7, 10 or from 12 to 19 depending on the image
  - Nucleus z focus : 0, 3, 4, 8 or from 10 to 19 depending on the image
  - Tubulin z focus : from 0 to 11, 15, 16, 17, 18 or 19 depending on the image

- **channels** :
    - nucleus :
        - marker : H2B-GFP
        - excitation wavelength : 475/40 nm
        - emission wavelength : 530/50 nm
    - tubulin :
        - marker : SIR-Tubulin
        - excitation light : 640/30 nm
        - emission light : 690/50 nm
- **dimension order** : TCZYX
- **images size** : 1, 1, 1, 2048, 2048
- **physical size x** : 0.10317460317460317 µm
- **physical size y** : 0.10317460317460317 µm
- **physical size z** : 0.26 µm
- **type** : uint16
- **format** : ome.tiff

## Image Acquisition

- **imaging method** : PC, Fluorescence
- **imaging instrument** : Inverted Zeiss Axio Observer 7, with temperature 37°C CO2 chamber. Zeiss Apo Plan. ORCA-Flash4 Hamamatsu monochrome camera( pixels, 6.5µm pixel size).
- **pixel size** : 110 nm
- **light source** : X-Cite 120LED
- **detector model** : OrcaFlash4LT HDCamC11440-42U
- **objective** :
    - **model** : LED XCite 120LED Plan-Apochromat
    - **immersion** : Oil
    - **lens numerical aperture (NA)** : 1.4
    - **nominal magnification** : 63x
    - **working distance** : 0.193 mm
    - **refractive_index** : 1.518

## Raw Image Notes

- **samples per pixel** : 1
- **z planes number** : 20
- **dimension order** : TCZYX
- **images size** : 13, 3, 20, 2048, 2048
- **channel order** : PC, H2B-GFP, SIR-Tubulin
- **type** : uint16
- **format** : .czi

# Study 30

## Authors


- **Valentin ASEI-CESCHINO**, CNRS, MRI, IGH, CRBM, France
  valentin.asei-ceschino@igh.cnrs.fr


- **Role :** VAC prepared the sample and made the acquisition.

- **France BioImaging node :** Montpellier
- **France BioImaging platform :** Montpellier Ressources Imagerie (MRI), Institut de Génétique Humaine (IGH), Centre de Recherche en Biologie cellulaire de Montpellier (CRBM)

## Description

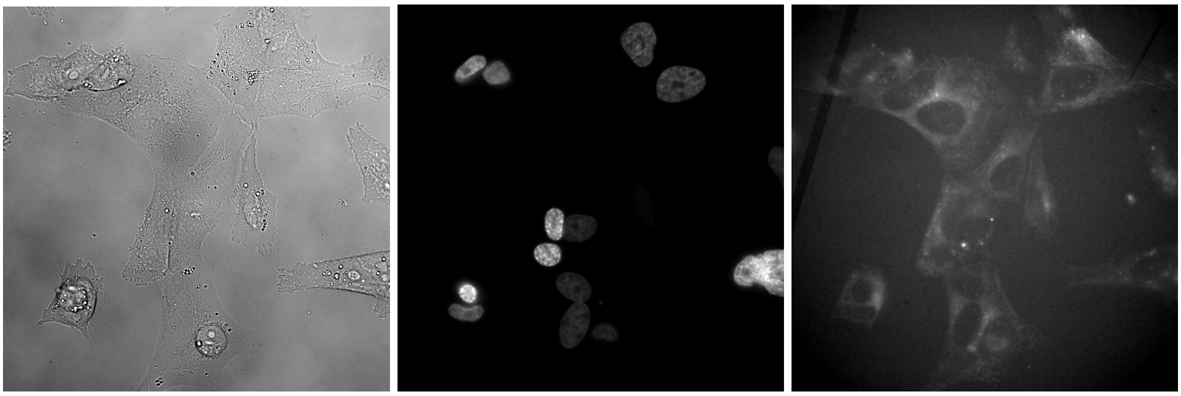

**a)** *image_2573_BF_z13* **b)** *image_2573_Nucleus* **c)** *image_2573_Tubulin*

**Fig.study 30:** *Example of images from Study 30.* ***a)*** *BF microscopy image of U2OS human cells.* ***b)*** *BF image equivalent but in fluorescence microscopy of H2B-GFP-labeled U2OS human nuclei.* ***c)*** *BF image equivalent but in fluorescence microscopy of SiR-tubulin-labeled U2OS human tubulin.*

## Study Component

- **name** : U2OS IGH (BF)
- **file list access** : json_file tsv_file

## Biosample

- **model** : human / homo sapiens
- **NCBI** : txid9606
- **biological entity** : U2OS stably expressing histone H2B fused to EGFP.
- **ATCC catalog number** : Not Referenced
- **description**: Cells not really healthy. Cells thawed the week before. Not dense.

## Specimen

- **sample preparation** : Cells were plated in glass bottom (#1,5) fluorodish in DMEM. Microtubules were stained with SiR Tubulin (1/2000) + Verapamil (1/2000) in fluorobrite for 5 hours at 37°C.
- **growth protocol** : DMEM Glutamax 10% SVF (Sérum de Veau foetal)

## Image Data

- **number of images** : 336 with :
  - 288 BF : image_<*n_img*>_BF_z<*n_z*>.ome.tiff (where *n_img* from 2,550 to 2,573 and *n_z from 0 to* 20
  - 24 Nucleus : image_<*n_img*>_Nucleus.ome.tiff (where *n_img* from 2,550 to 2,573)
  - 24 Tubulin : image_<*n_img*>_Tubulin.ome.tiff (where *n_img* from 2,550 to 2,573)
- **z planes :**
  - number : 1
  - BF z focus : 0, 2, 4, 16, 17, 18 or 19 depending on the image
  - Nucleus z focus : 0, 2, 3, 4, 13, 15, 16 or 19 depending on the image
  - Tubulin z focus : from 1 to 7, 10, 11, 15 or 19 depending on the image

- **channels** :
    - nucleus :
        - marker : H2B-GFP
        - excitation wavelength : 475/40 nm
        - emission wavelength : 530/50 nm
    - tubulin :
        - marker : SIR-Tubulin
        - excitation light : 640/30 nm
        - emission light : 690/50 nm

- **dimension order** : TCZYX
- **images size** : 1, 1, 1, 2048, 2048
- **physical size x** : 0.10317460317460317 µm
- **physical size y** : 0.10317460317460317 µm
- **physical size z** : 0.26 µm
- **type** : uint16
- **format** : ome.tiff

## Image Acquisition

- **imaging method** : PC, Fluorescence
- **imaging instrument** : Inverted Zeiss Axio Observer 7, with temperature 37°C $CO_2$ chamber. Zeiss Apo Plan. ORCA-Flash4 Hamamatsu monochrome camera( pixels, 6.5µm pixel size).
- **pixel size** : 110 nm
- **light source** : X-Cite 120LED
- **detector model** : OrcaFlash4LT HDCamC11440-42U
- **objective** :
    - **model** : LED XCite 120LED Plan-Apochromat
    - **immersion** : Oil
    - **lens numerical aperture (NA)** : 1.4
    - **nominal magnification** : 63x
    - **working distance** : 0.193 mm
    - **refractive_index** : 1.518

## Raw Image Notes

- **samples per pixel** : 1
- **z planes number** : from 5 to 20
- **dimension order** : TCZYX
- **images size** : 13, 3, 5 to 20, 2048, 2048
- **channel order** : BF, H2B-GFP, SIR-Tubulin
- **type** : uint16
- **format** : .czi
