# Supplementary material for: 2D Multimodal Image Collection for Fluorescence Prediction from Transmitted Light Microscopy
Source: Sci Data. 2026 Mar 24;13:743. doi: 10.1038/s41597-026-07004-w (PMC13184337; doi:10.1038/s41597-026-07004-w)
Supplement: Supplementary file 2 — Supplementary Table 1 [file 41597_2026_7004_MOESM2_ESM.docx]

| Database | | | |
| --- | --- | --- | --- |
| **Study**  total study number: **30** | **Acquisition sets** total image set number:  **2,574** | **Images** total image number:56,984 | |
|  |  | **Transmitted Light** total image number: **52,382** | **Fluorescence** total image number: **4,780** |
| **S1**  129 images | 43 acquisition sets  (from image 0 to 42) | 43 BF | 43 Nucleus 43 Mitochondria |
| **S2**  144 images | 10 acquisition sets  (from image 43 to 52) | 125 DIC | 10 Nucleus 10 Mitochondria |
| **S3**  125 images | 8 acquisition sets  (from image 53 to 60) | 103 BF | 8 Nucleus 7 Mitochondria 7 Actin |
| **S4**  408 images | 31 acquisition sets  (from image 61 to 91) | 315 DIC | 31 Nucleus 31 Mitochondria 31 Tubulin |
| **S5**  140 images | 10 acquisition sets  (from image 92 to 101) | 120 PC | 10 Nucleus 10 Actin |
| **S6**  130 images | 10 acquisition sets  (from image 102 to 111) | 110 PC | 10 Nucleus 10 Actin |
| **S7**  150 images | 10 acquisition sets  (from image 112 to 121) | 130 DIC | 10 Nucleus 10 Tubulin |
| **S8**  168 images | 8 acquisition sets  (from image 122 to 129) | 152 BF | 8 Nucleus 8 Mitochondria |
| **S9**  168 images | 8 acquisition sets  (from image 130 to 137) | 152 BF | 8 Nucleus 8 Mitochondria |
| **S10**  232 images | 8 acquisition sets  (from image 138 to 145) | 216 BF | 8 Nucleus 8 Mitochondria |
| **S11**  116 images | 4 acquisition sets  (from image 146 to 149) | 108 BF | 4 Nucleus 4 Mitochondria |
| **S12**  232 images | 8 acquisition sets  (from image 150 to 157) | 216 PC | 8 Nucleus 8 Mitochondria |
| **S13**  232 images | 8 acquisition sets  (from image 158 to 165) | 216 PC | 8 Nucleus 8 Mitochondria |
| **S14**  184 images | 8 acquisition sets  (from image 166 to 173) | 168 PC | 8 Nucleus 8 Mitochondria |
| **S15**  116 images | 4 acquisition sets  (from image 174 to 177) | 108 PC | 4 Nucleus 4 Mitochondria |
| **S16**  693 images | 33 acquisition sets  (from image 178 to 210) | 660 DIC | 33 Mitochondria |
| **S17**  902 images | 41 acquisition sets  (from image 211 to 251) | 820 DIC | 41 Nucleus 41 Tubulin |
| **S18**  253 images | 23 acquisition sets  (from image 252 to 274) | 207 DIC | 23 Nucleus 23 Mitochondria |
| **S19**  253 images | 23 acquisition sets  (from image 275 to 297) | 207 DIC | 23 Nucleus 23 Mitochondria |
| **S20**  276 images | 23 acquisition sets  (from image 298 to 320) | 207 DIC | 23 Nucleus 23 Mitochondria 23 Tubulin |
| **S21**  253 images | 23 acquisition sets  (from image 321 to 343) | 207 DIC | 23 Nucleus 23 Tubulin |
| **S22**  253 images | 23 acquisition sets  (from image 344 to 366) | 207 DIC | 23 Nucleus 23 Mitochondria |
| **S23**  253 images | 23 acquisition sets  (from image 367 to 389) | 207 DIC | 23 Nucleus 23 Mitochondria |
| **S24**  276 images | 23 acquisition sets  (from image 390 to 412) | 207 DIC | 23 Nucleus 23 Mitochondria 23 Tubulin |
| **S25**  29,647 images | 1,289 acquisition sets  (from image 413 to 1,701) | 27,069 BF | 1,289 Nucleus 1,289 Mitochondria |
| **S26**  13,501 images | 587 acquisition sets  (from image 1,702 to 2,288) | 12,914 BF | 587 Nucleus |
| **S27**  6,269 images | 197 acquisition sets  (from image 2,289 to 2,485) | 5,875 PC | 197 Nucleus 197 Mitochondria |
| **S28**  344 images | 16 acquisition sets  (from image 2,486 to 2,501) | 320 PC | 8 Nucleus 16 Mitochondria |
| **S29**  801 images | 48 acquisition sets  (from image 2,502 to 2,549) | 705 PC | 48 Nucleus 48 Tubulin |
| **S30**  336 images | 24 acquisition sets  (from image 2,550 to 2,573) | 705 PC | 24 Nucleus 24 Tubulin |

***Table 1 | Overview of the Light My Cells database collection.***

*Summary of the 30 studies included in the Light My Cells database, reporting for each study the total number of images, acquisition sets, transmitted-light modalities (Bright Field – BF, Phase Contrast – PC, Differential Interference Contrast – DIC), and associated fluorescence channels. An acquisition set corresponds to a single biological field of view and groups all transmitted-light and fluorescence images acquired for that scene. Global totals for studies, acquisition sets, and images are reported at the top of the table.*
